# The Effects of Cranberry Consumption on Glycemic and Lipid Profiles in Humans: A Systematic Review and Meta-Analysis of Randomized Controlled Trials

**DOI:** 10.3390/nu16060782

**Published:** 2024-03-09

**Authors:** Xiangrui Li, Wenqing Chen, Jiayue Xia, Da Pan, Guiju Sun

**Affiliations:** Key Laboratory of Environmental Medicine and Engineering of Ministry of Education, Department of Nutrition and Food Hygiene, School of Public Health, Southeast University, Nanjing 210009, China

**Keywords:** cranberry, blood lipid, blood glucose, metabolism, meta-analysis

## Abstract

This study aims to update the evidence and clarify whether cranberry possesses lipid-lowering and hypoglycemic properties in humans. PubMed, Web of Science, and Scopus were searched to identify relevant articles published up to December 2023. In total, 3145 publications were reviewed and 16 of them were included for qualitative synthesis and meta-analysis. Stata 15.0 and Review Manager 5.4 were applied for statistical analyses. The results revealed a significant decrease in the total cholesterol to high-density lipoprotein cholesterol ratio (TC/HDL-C) (MD = −0.24; 95% CI: −0.45, −0.04; *p*_effect_ = 0.02) and homeostasis model assessment of insulin resistance (HOMA-IR) (MD = −0.59; 95% CI: −1.05, −0.14; *p*_effect_ = 0.01) with cranberry consumption. However, it did not influence total cholesterol (TC), high-density lipoprotein cholesterol (HDL-C), low-density lipoprotein cholesterol (LDL-C), triglyceride (TG), fasting blood glucose (FBG), glycated hemoglobin (HbA1c), and fasting insulin. In subgroup analysis, cranberry consumption in dried form (capsules, powder, and tablets) was found to significantly decrease the fasting insulin level (three studies, one hundred sixty-five participants, MD = −2.16; 95% CI: −4.24, −0.07; *p*_effect_ = 0.04), while intervention duration, health conditions, and dosage of polyphenols and anthocyanins had no impact on blood lipid and glycemic parameters. In summary, cranberry might have potential benefits in regulating lipid and glucose profiles.

## 1. Introduction

The cranberry is one of the common berries with several varieties, including *Vaccinium macrocarpon*, *Vaccinium oxycoccus*, and *Vaccinium microcarpum,* all of which are rich in various nutrients [1,2]. Cranberries are primarily composed of water and carbohydrates, which make them a low-calorie food source. Cranberries are also rich in dietary fiber, minerals, and vitamins, including potassium, magnesium, calcium, vitamin C, and vitamin E. Moreover, cranberries are recognized as one of the important dietary sources of bioactive compounds, including phenolic acids, flavonoids, anthocyanins, and tannins, endowed with antibacterial and anti-inflammatory properties [2]. These bioactive compounds have garnered considerable interest from researchers and have been extensively studied in recent years due to their various health benefits. The role of cranberry in preventing urinary tract infections in both adults and children has been widely acknowledged [3]. Some researchers now believe that cranberry can protect against cardiovascular diseases, reduce the risk of various cancers and metabolic diseases, and even prevent tooth decay, periodontitis, and *Helicobacter pylori* [2,4,5].

Cardiovascular disease (CVD) is a non-communicable disease that seriously threatens human health with characteristics of high disability and a high mortality rate, causing a global burden [6]. Individuals with CVD may have changes in insulin sensitivity, glucose tolerance, lipid profiles, and oxidative stress biomarkers [6]. Polyphenols in cranberry have beneficial properties in the modulation of glucose and lipid metabolism, which has been confirmed in a variety of in vitro experiments and animal models [7,8,9]. Niesen et al. isolated the active compound of cranberry and found that polyphenols extracted from cranberry inhibited lipid accumulation and promoted lipolysis, which had a positive regulatory effect on lipid metabolism [7]. Peixoto et al. fed rats a high-fat diet and cranberry extract and evidenced that cranberry extracts improve lipid profiles and protect the liver and adipose tissue from oxidative damage [8]. A study from Hong Kong also displayed that cranberry juice consumption improved total cholesterol (TC), triacylglycerols (TG), and high-density lipoprotein cholesterol (HDL-C) in ovariectomized rats [9].

However, the antioxidant activity of dietary anthocyanins in vivo and in vitro as well as the metabolic effect on blood glucose and lipid levels in human beings might be different; the benefits mentioned above about the consumption of cranberry currently remain controversial in clinical trials. Research from Taiwan displayed that daily consumption of cranberry extracts significantly decreased blood TC, low-density lipoprotein cholesterol (LDL-C), and the TC/HDL-C ratio in type 2 diabetes [10]. A US study found cranberries reduce blood TG, fasting blood glucose (FBG), and homeostatic model assessment of insulin resistance (HOMA-IR) levels [11]. However, some other studies did not consider cranberries to have a significant impact on the biomarkers of blood lipid status [12,13].

In the last few years, new research has increasingly been carried out on the relationship between cranberry and lipid and glucose metabolism [14,15]. To update the evidence and conclude the inconsistent results of clinical trials, this systematic review and meta-analysis was designed to clarify whether cranberries have lipid-lowering and hypoglycemic properties and to investigate the beneficial effects of cranberries on different subgroups.

## 2. Materials and Methods

This meta-analysis and systematic review was conducted following the Preferred Reporting Items for Systematic Reviews and Meta-analyses (PRISMA) and registered as ID CRD42024509248.

### 2.1. Search Strategy

A literature search was performed in electronic databases including PubMed, Web of Science, and Scopus to recognize relative articles by using the following search terms: (Metabolic OR Metabolism OR lipoprotein OR cholesterol OR triglyceride OR lipid profile OR TG OR TC OR LDL OR VLDL OR blood lipid OR APO OR apolipoprotein) AND (cranberry OR *Vaccinium macrocarpon* OR *Vaccinium microcarpum* OR *Vaccinium oxycoccus*). The database was searched up to December 2023. Only literature published in English was included. The relevant articles’ reference lists were also checked to avoid omissions. Detailed search strategies are shown in Appendix A.

### 2.2. Study Inclusion and Exclusion Criteria

Two authors independently screened and evaluated the literature according to the title and abstract. The study inclusion criteria were as follows: (1) randomized controlled trials (RCTs) with either parallel or cross-over design; (2) participants aged over 18 years old; (3) the experimental group performed cranberry supplementation as their intervention; (4) the results contained blood lipid or glucose related indicators, including TC, TG, HDL-C, LDL-C, FBG, fasting insulin, and glycosylated hemoglobin A1C (HbA1c), HOMA-IR. Studies were excluded if they included the follows: (1) in vitro studies, animal studies, non-interventional studies, or non-randomized controlled trials; (2) duplicate databases; (3) incomplete research or did not provide sufficient data related to lipid profiles or blood glucose indexes; (4) supplemented cranberries in combination with other non-polyphenolic antioxidants such as Omega-3 and L-citrulline; (5) has an intervention time less than two weeks.

### 2.3. Data Extraction

For each relevant study, the first author’s name, year of publication, country, study design, sample size (number of participants in the intervention and control groups), subjects’ character (gender and age), intervention brand and form, cranberry and phenolic content, study duration, and outcomes related to blood lipid and glucose were extracted and collected by authors. Units of blood lipid profiles (TC, TG, HDL-C, and LDL-C) and fasting glucose are unified as mmol per liter (mmol/L). Conversion equations were shown as follows: for TC, HDL-C, and LDL-C, 1 mg/dL was equaled to 0.0258 mmol/L; for TG, 1 mg/dL was converted to 0.0113 mmol/L [16]; and for fasting blood glucose, 1 mg/dL was converted to 0.0555 mmol/L [17]. For the parallel study, the endpoint and baseline for both the control and intervention groups were extracted. In contrast, for the crossover trials, the endpoint and baseline of the control period and the endpoint and baseline of the intervention period were calculated separately. In addition, the last endpoint values were extracted if there were several endpoints.

### 2.4. Quality Assessment of Meta-Analysis

Cochrane’s risk-of-bias tool for randomized trials is a standard tool used to evaluate the quality of RCTs [18]. Two authors individually assessed each included study using the following seven main aspects of the Cochrane risk-of-bias tool one by one: (1) random sequence generation (selection bias); (2) allocation concealment (selection bias); (3) blinding of participants and personnel (performance bias); (4) blinding of outcome assessment (detection bias); (5) incomplete outcome data (attrition bias); (6) selective reporting (reporting bias); (7) other bias. Based on the evaluation criterion of Cochrane’s recommendations, the overall risk-of-bias judgment of each aspect was classified as “high risk”, “unclear risk”, and “low risk”. All the inconsistencies and uncertainties were discussed with a third researcher.

### 2.5. Statistical Analyses

In this meta-analysis, summarized effect sizes were represented using the mean difference (MD) with 95% confidence intervals. The mean value was calculated using the endpoint value minus the baseline value for each indicator. Changes in standard deviation (SD) were tested using the following equations: SD_change_ = (SD^2^_baseline_ + SD^2^_endpoint_ − 2R × SD_baseline_ × SD_endpoint_)^1/2^, correlation coefficient R = 0.5 [19]. When data were represented using the standard error (SE) with 95% CI, or medium or percentiles, they were all appropriately converted [20,21,22]. In addition, the heterogeneity of the results in the different studies was detected using the χ^2^ test. Random-effect or fixed-effect models were applied based on the degree of heterogeneity. Moreover, funnel plots and Egger’s test were conducted to investigate publication bias if there were more than ten studies [23]. Subgroup analyses were implemented for indicators with more than five articles to distinguish the sources of heterogeneity, including dosage form, intervention duration, health conditions, cranberry content, and the dosage of phenolic substances. Sensitivity analysis was performed by changing the inclusion and exclusion criteria to exclude high-risk studies and to identify the stability and reliability of research results. Statistical analyses were completed using Stata 15.0 and Review Manager version 5.4 (Cochrane Collaboration software). *p* < 0.05 in this analysis was statistically significant.

## 3. Results

### 3.1. Search Results

**Figure 1:**
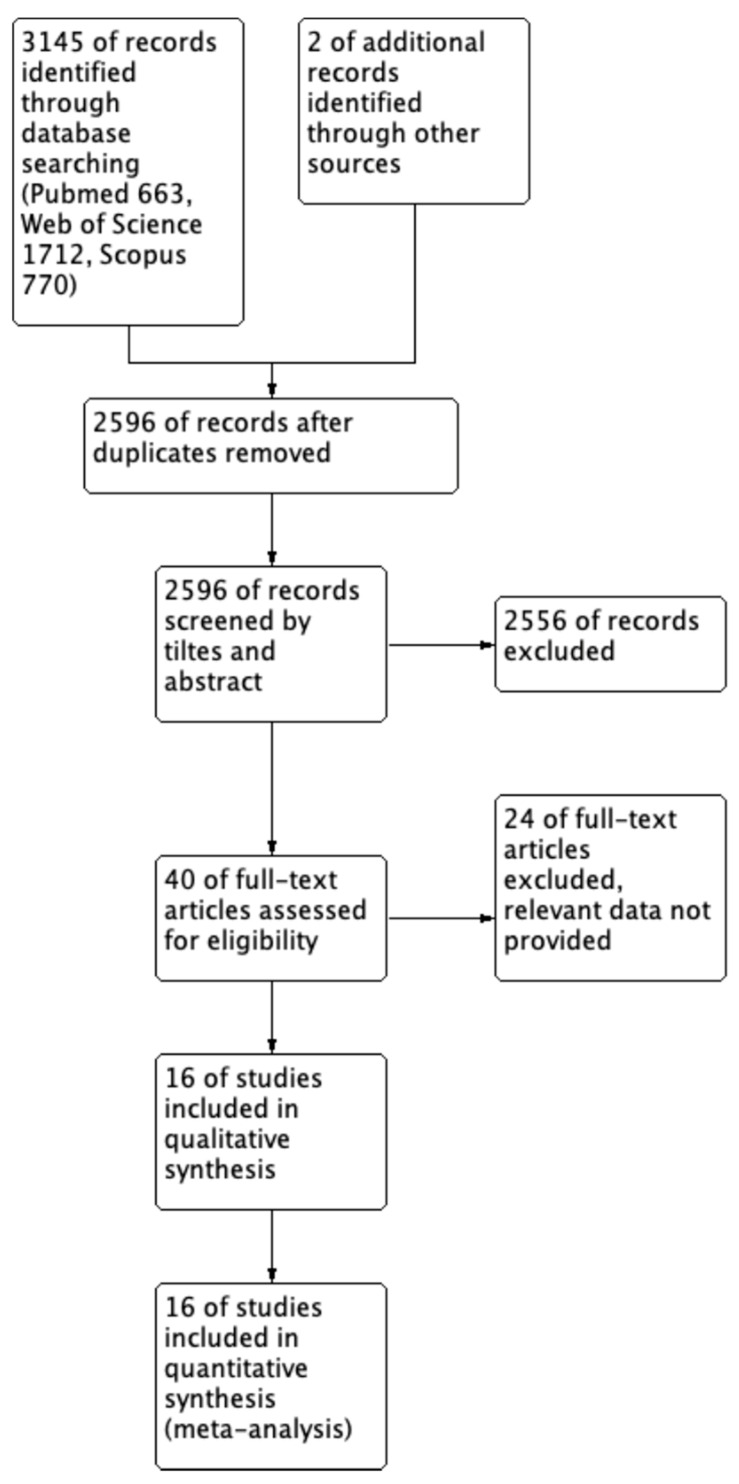
Flowchart.

A total of 3145 studies were explored based on the search terms (PubMed = 663, Web of Science = 1712, and Scopus = 770). Two additional records were obtained from references of previous studies. A total of 2596 records were screened by title and abstracts after removing duplicate research. Then, 2556 studies were excluded because of in vitro or animal studies and irrelevant outcomes. Finally, 40 full-text studies assessed the above criteria preliminary, and 16 of them were included for qualitative synthesis and meta-analysis [10,11,12,13,14,15,24,25,26,27,28,29,30,31,32,33]. The flow chart for the literature search is shown in Figure 1.

### 3.2. Study Characteristics

Table 1 lists the characteristics and main results of sixteen included studies published from 2006 to 2023 [10,11,12,13,14,15,24,25,26,27,28,29,30,31,32,33] and nine published in the past five years [14,15,27,28,29,30,31,32,33]. In total, 708 participants aged 18 to 80 were systematically reviewed in this analysis. Seven studies were from North America (United States and Canada) [11,24,25,26,27,30,31], five from Asia (Iran and Taiwan) [10,13,28,29], and four from Europe (United Kingdom and Germany) [12,15,32,33]. Only two of them were placebo-controlled crossover studies [25,31], whereas others were placebo-controlled, parallel design trials. Five studies conducted experimental investigations of healthy populations [11,12,15,32]; other studies selected patients with overweight or obesity, diabetes, CVD, metabolic syndrome, or non-alcoholic fatty liver (NAFLD). Cranberry supplements were provided in different forms (juice [11,12,13,15,24,25,26,27,28,30,31], capsules [10,14], powder [32,33], or tablets [29]) with various doses and the intervention length ranged from 2 weeks to 6 months. The cranberry dosage varied among studies with capsules, tablets ranging from 144 to 1500 mg per day, powder 9 g per day, and cranberry juice ranging from 240 to 750 mL per day [10,11,12,14,15,24,25,26,27,28,29,30,31,32,33]. Regarding the intervention dosage of phenolic contents, the content of polyphenols in the included studies ranged from 158 to 2250 mg, and anthocyanins varied from 2.2 to 552 mg [10,11,14,15,24,25,26,27,28,29,30,31,32,33]. Four studies did not provide detailed amounts of total polyphenols [10,13,14,29], and five studies did not mention anthocyanin content [10,13,14,27,29]. The polyphenol and anthocyanin doses of the cranberry capsules used by Lee et al. were obtained and calculated from a Spanish study using identical cranberry capsules from the same company [10,34]. Shirazi et al. and Hormoznejad et al. studied cranberry powder, equivalent to 13 g and 26 g of dried cranberries, respectively [14,29]. Studies that used the same brand of cranberry powder were not found, so the total polyphenol and anthocyanin contents of the cranberry powder in these two studies were not available. Shidfar et al. did not provide a specific cranberry juice brand or concentration, and the doses failed to be estimated [13]. In addition, Paquette et al. only mentioned the polyphenol content without mentioning the anthocyanin content [27]. Since no other studies were conducted using the same beverage, the anthocyanin content could not be estimated.

### 3.3. Quality of the Studies and Publication Bias

**Figure 2:**
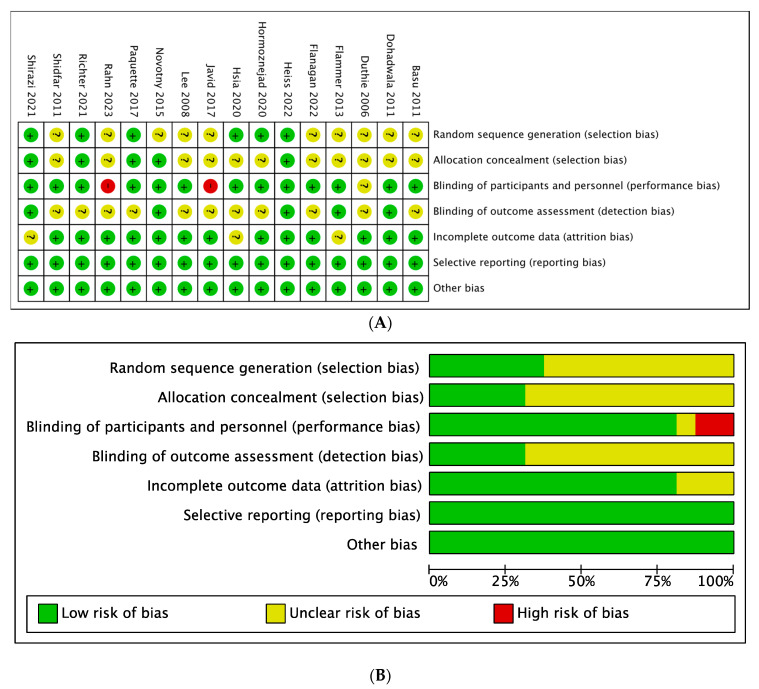
Risk of bias summary for included studies. (**A**) Bias risk summary. “+” = low risk of bias, “?” = unclear risk of bias, and “-” = high risk of bias. (**B**) Bias risk graph. Green = low risk of bias, yellow = unclear risk of bias, and red = high risk of bias [10,11,12,13,14,15,24,25,26,27,28,29,30,31,32,33].

Each trial in the review was assessed for risk of bias using the Cochrane risk-of-bias tool (Figure 2A,B). Eleven studies did not provide a detailed description of the method used to generate the assignment sequence. In twelve of them, the method of hiding the allocation sequence was insufficient to determine whether the allocation of the intervention was visible before or during the inclusion process. Javid et al. and Rahn et al. implemented their trials without the blinding method, which had high risks of performance bias [15,28]. In addition, Duthie et al. did not provide enough information relevant to the effectiveness of blinding participants [12]. Regarding detection bias, whether outcome evaluators were blinded in twelve studies was unclear. Three reports lacked detailed information on lost subjects [14,26,30]. All sixteen trials were at low risk of reporting bias and other relevant biases. In terms of publication bias, funnel plots and Egger’s test were utilized to assess publication bias for aspects that had at least 10 studies (Supplementary Figures S1–S5 and Table 2) [23]. Potential publication bias was detected in terms of HDL-C according to Egger’s test.

### 3.4. The Effects of Cranberry Supplementation on Blood Lipid Profiles

**Figure 3:**
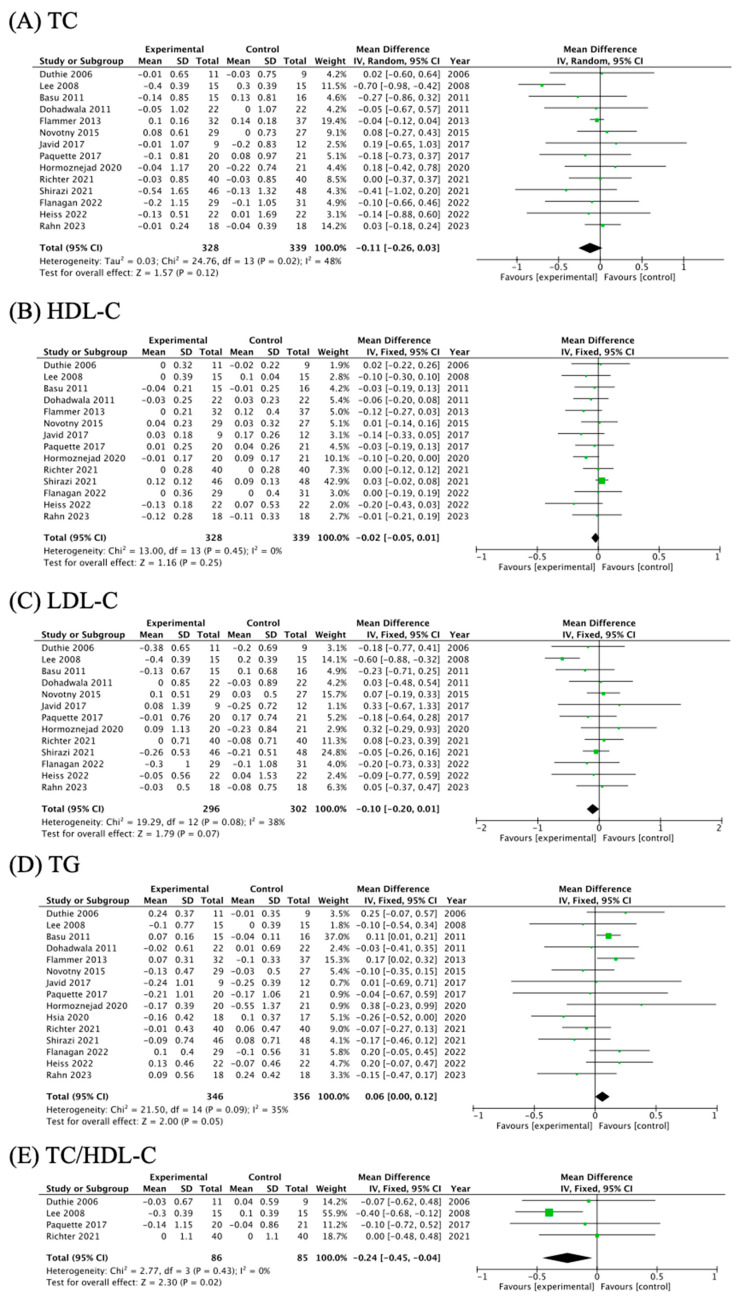
The effects of cranberry supplementation on lipids profiles: TC: total cholesterol; LDL-C: low-density lipoprotein cholesterol; HDL-C: high-density lipoprotein cholesterol; TG: triglyceride [10,11,12,14,15,24,25,26,27,28,29,31,32,33].

The summary of results from meta-analyses is listed in Table 2. Forest plots of the impact of cranberry supplementation on blood lipid profiles are presented in Figure 3A–E. The pooled results of the meta-analysis showed that cranberry had a significant effect on TC/HDL-C (MD = −0.24; 95% CI: −0.45, −0.04; *p*_effect_ = 0.02) without significant heterogeneity between studies (I^2^ = 0%, *p*_heterogeneity_ = 0.43). However, no significant decrease or increase was observed between cranberry supplementation and other blood lipid profiles: TC (MD = −0.11; 95% CI: −0.26, 0.03; *p*_effect_ = 0.12), HDL-C (MD = −0.02; 95% CI: −0.05, 0.01; *p*_effect_ = 0.25), LDL-C (MD = −0.10; 95% CI: −0.20, 0.01; *p*_effect_ = 0.07), and TG (MD = 0.06; 95% CI: 0.00, 0.12; *p*_effect_ = 0.05).

### 3.5. The Effects of Cranberry Supplementation on Glycemic Parameters

**Figure 4:**
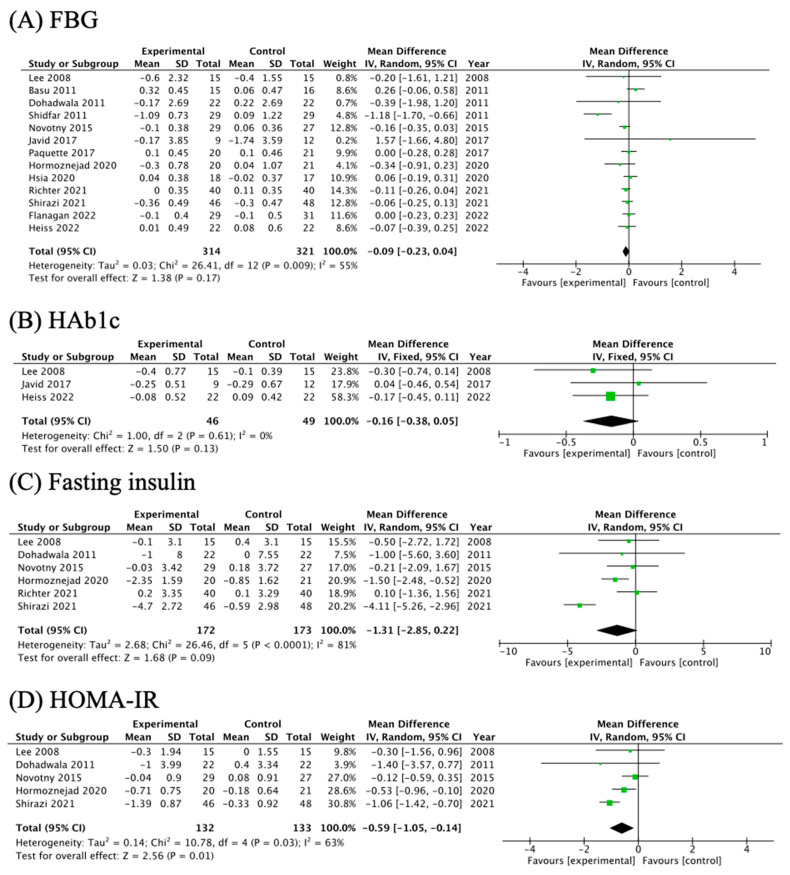
The effects of cranberry supplementation on glycemic related indicators: FBG: fasting blood glucose; HbA1c: glycosylated hemoglobin A1C; HOMA-IR: homeostatic model assessment of insulin resistance [10,11,13,14,24,25,27,28,29,30,31,32,33].

In terms of glycemic indicators, HOMA-IR was investigated in five studies, and the pooled estimates suggested that cranberry significantly reduced HOMA-IR (MD = −0.59; 95% CI: −1.05, −0.14; *p*_effect_ = 0.01; Figure 4D), whereas cranberry supplementation did not show significant alterations to FBG (MD = −0.09; 95% CI: −0.23, 0.04; *p*_effect_ = 0.17), HbA1c (MD = −0.16; 95% CI: −0.38, 0.05; *p*_effect_ = 0.13), and fasting insulin (MD = −1.31; 95% CI: −2.85, 0.22; *p*_effect_ = 0.09) (Figure 4A–C).

### 3.6. Subgroup Analysis and Sensitivity Analysis

The included studies were subgroups analyzed by dosage form, intervention duration, health conditions, cranberry content, and dosage of phenolic substances. The results are provided in Supplementary Figures S6–S35. TC/HDL-C, HbA1c, and HOMA-IR were not determined by subgroups due to limited articles. Subgroup analysis stratified by the dosage form indicated that cranberry administrated in dried form, including capsules, powder, and tablets, produced a significant reduction in fasting insulin (MD = −2.16; 95% CI: −4.24, −0.07; *p*_effect_ = 0.04). In contrast, TC, HDL-C, LDL-C, and FBG were not observed to have significant outcomes in subgroups.

For sensitivity analysis, two studies with high-risk aspects of bias were excluded after altering the inclusion and exclusion criteria. No significant changes were found in the results, which suggests the results are stable.

## 4. Discussion

In this meta-analysis, the latest RCTs in humans were included and pooled for analysis. A significant reduction in the TC and HDL ratio and HOMA-IR were observed in cranberry consumption groups. In contrast, cranberry efficacy on single blood lipid and glycemic biomarkers (TC, LDL-C, HDL-C, TG, FBG, HbA1c, and fasting insulin) did not reach significance in pooled effect size. In a subset analysis of dosage form, cranberry administered in tablets, powder, or capsules significantly declined insulin, while no significant differences were discovered in other subgroup analyses.

The TC/HDL-C ratio is a comprehensive indicator. TC is an atherogenic marker, and HDL-C is an antiatherogenic lipid parameter. An increased number of studies now suggest that the ratio between them can jointly provide more information than an isolated indicator [35,36]. As of recently, the TC/HDL-C ratio is regarded as an essential risk factor and a more potent predictor of CVD incidence than TC, HDL-C, and LDL-C [36,37]. Studies demonstrated that a high TC/HDL-C ratio, especially more than 5, is considered to indicate an elevated risk of CVD, probably because of an imbalance in the proportion of cholesterol lipoproteins [36,37]. Cranberry consumption significantly reducing this ratio might suggest that cranberry potentially benefits lipid modulation. The possible mechanism involved was that cranberries were rich in polyphenols, especially anthocyanins, phenolic acids, and flavonoids [2]. Anthocyanins were considered to have the potential ability to prevent hepatocellular lipid accumulation and lipogenesis by reducing sterol-regulated element binding protein 1c (Srebp1c), promoting lipolysis by activating peroxisomes proliferator-activated receptor (PPAR) in hepatocytes, and reducing oxidative stress [38]. In this meta-analysis, cranberry intake did not significantly drop single lipid markers such as TC, HDL-C, LDL-C, and TG. Two studies indicated a significant decrease in blood LDL-C after consuming cranberries after 12 weeks of intervention [10,32]. Previous studies also found that cranberry intake resulted in a reduction in plasma oxidized LDL, a biomarker of oxidative stress, and elevation in antioxidant capacity [24,39]. Regarding blood HDL-C, a previous meta-analysis revealed HDL-C concentration increased significantly in the group under 50 years of age [40]. Also, a study conducted by Ruel et al. discovered a significant increase in plasma HDL-C concentration in male subjects after 4 weeks of taking cranberry juice [41]. They supposed that cranberry might have the ability to reduce the clearance of HDL-C particles and quercetin, a bioactive compound present in cranberry, and activate the expression of the HDL-C-associated enzyme paraoxonase-1 [41]. In contrast, a study included in this analysis from Dohadwala et al. observed a reduction in blood HDL-C [25], and a study conducted in non-alcoholic fatty liver patients found an elevation in blood HDL-C of the placebo group [29]; no significant beneficial effects on HDL-C were detected in other included studies.

In this research, several indices measured glycemia, including FBG, HbA1c, fasting insulin, and HOMA-IR. HOMA-IR significantly declined in intervention groups compared with placebo groups, while other parameters showed no significant differences. HOMA-IR is also a correlation value and can be calculated using fasting insulin and FBG [42]. HOMA-IR is a practical way to assess insulin resistance levels related to the risk of CVD and type 2 diabetes [42]. Three included research studies demonstrated similar findings on HOMA-IR [11,14,29]. Apart from the influence on HOMA-IR, Novotny et al. also discovered that individuals with higher baseline FBG had a more significant reduction after drinking cranberry juice [11]. Two Iran studies found that cranberry could affect insulin [14,29]. The glycemic regulation mechanism of cranberry was possibly attributed to the polyphenol component mentioned by many animal studies [43,44,45]. Anthocyanins can rapidly degrade glucagon-like peptide (GLP-1) and gastric inhibitory peptide (GIP), promote the function of insulin secretion of islet beta cells, and then improve HOMA-IR [46]. Anthocyanins can also inhibit the activity of intestinal digestive enzymes and help control blood sugar by slowing the digestion and absorption of carbohydrates [47]. There was also an acute study conducted on postprandial blood glucose of obese people with type 2 diabetes, and it found that the postprandial rise in blood glucose was significantly lower in the cranberry group than in the control group [48].

The intervention form of cranberry was a considerable factor. After stratification by dosage form, a significant reduction in insulin was observed in cranberry given in dried form (capsules, powder, and tablets) compared with juice form. In addition, the dried form of cranberries did not show a significant decline in TC and LDL-C, but the results displayed a downward trend. Five included studies used capsules, tablets, or powder as an intervention pathway [10,14,29,32,33]. In these five studies, Hormoznejad et al. and Shirazi et al. both found that insulin and HOMA-IR declined in the intervention groups [14,29]. In addition, Lee et al. and Flanagan et al. recognized cranberry had a significant effect on lipid profiles [10,32]. The possible cause why the dried form had a more significant effect than the juice form was that they juice form had more free sugars, such as mono- and di-saccharides, and more energy than capsules or powder, which might slightly affect metabolism [31,49]. Eleven included studies used a liquid form and the free sugar contents were various. Most drinks of these studies contained 6 to 10 g of added sugar per day, while the interventional beverages in a study by Rahn et al. provided 51.5 g of fructose and glucose [15]. Meanwhile, this study did not conclude any benefit of cranberry on blood sugar and lipids. Moreover, cranberry powder, tablets, or capsules have some advantages over fruit juice in implementation. The effectiveness of cranberry juice in a non-study population may depend on the amount and timing of cranberry intake, but price, calories, and taste may reduce adherence in this population [50]. One study analyzed cranberry powder, with the total polyphenol compounds ranging from approximately 600 to 6000 mg per kg dry matter depending largely on the composition of the extracted juice and drying techniques [51]. It was reported that 145 mg of cranberry extract was almost equal to 240 mL of 27% pure cranberry juice [52]. The dried form of cranberry provides an additional way to supplement cranberries without considering the seasonality of the fruit, which is more advantageous for storage, feasibility, and acceptability.

The duration of the intervention varied across 16 studies, ranging from 2 weeks to 6 months. In this analysis, cranberry consumption of more than 8 weeks did not generate significant differences in any of the lipid and blood glucose indicators compared with the short-term intervention. However, some possibilities of decline were observed in the fields of TC, FBG, and fasting insulin in long-term intervention (≥8 weeks) groups. Currently, there is a lack of consistent conclusion on the duration of intervention, and no study has proven that there was any linear relationship between the duration of intervention and blood lipid and glucose levels. In a previous meta-analysis of blueberry and cranberry consumption, a significant difference was found in glycated hemoglobin levels between different intervention times (stratified by 8 weeks) [53]. Another study on cranberry and freeze-dried berries found elevated HDL during a 4–6-week intake, but no similar benefit was seen after less than four weeks of intervention [54]. In contrast, some studies considered the bioavailability of polyphenols to be low [55]. For example, the maximum concentration of anthocyanins in plasma was detected between 1 to 3 h and rapidly removed from the blood [55]. However, some of the data on polyphenol bioavailability only consider the presence of intact polyphenols in the blood [56]. In humans, these polyphenols may be catabolized into metabolites and synergistic effects between these polyphenol metabolites may explain the health-promoting properties [57,58]. The biological activities of these metabolites remain to be investigated.

Among the different health status groups, there were no significant differences regarding tested parameters. However, patients with metabolic diseases showed a decreasing trend in TC, LDL-C, and fasting insulin values. Patients with metabolic diseases mostly have abnormal glucose metabolism and dyslipidemia compared to a healthy population. Polyphenols improve glucose homeostasis and lipid profiles via multiple mechanisms of action in the liver, fat cells, and pancreatic beta cells, as mentioned before, as well as by mediating the gut microbiota [46,59]. Current in vivo studies were not entirely consistent. Previous reviews found berry consumption reduced fasting blood glucose and HbA1c and improved insulin resistance in diabetes [53,60]. In terms of lipid profiles, a meta-analysis from Wilken et al. concluded that LDL-C decreased and risk factors of metabolic syndrome improved due to berry intake [54]. Regarding NAFLD, anthocyanins can regulate the glycemic and lipid metabolism of this population [61]. Nevertheless, Pourmasoumi et al. considered there to be no significant benefits to diabetes after cranberry interventions [40].

It is worth considering that the dosage of polyphenols and anthocyanin may be potential factors in regulating blood lipids and glycemic indicators. Subgroup analyses were conducted based on the dosage of polyphenols and anthocyanin, yet no significant reduction was observed within subgroups for each parameter. Four included studies lacked specific information on their polyphenol dosage and five studies did not mention their anthocyanin amount. Therefore, some of the data were estimated from other literature using the same products. The content of polyphenols in the included studies ranged from 158 to 2250 mg, and anthocyanins varied from 2.2 to 552 mg [10,11,12,14,15,24,25,26,27,28,29,30,31,32,33]. The dosages of cranberry interventions in animal experiments were much higher than that in human experiments. Anhe et al. gave high-fat/high-sucrose-fed mice 200 mg/kg of cranberry extract (37.4% total polyphenols and 3.3% anthocyanins) by gavage and found a significant reduction in hepatic TG levels [44]. Currently, very few studies provide specific recommendations for cranberry supplementation regarding lipid and glucose regulation in humans. There was also little evidence of a dose–response relationship between cranberry and lipid and glucose metabolism. Paquette et al. mentioned polyphenol doses below 800 mg may have metabolic benefits [27]. Another study found that, although the effect of cranberry on blood lipids was not significant, meta-regression showed a correlation between lipids and the cranberry supplement dose [40]. And, the authors suggested higher doses of cranberry may be beneficial for blood lipid regulation [40]. In addition to anthocyanins, there are also proanthocyanins and phenolic acids in cranberries [2]. However, since most studies did not mention the content of each kind of polyphenol, this study did not conduct the subgroup analysis based on proanthocyanins and phenolic acids. As a polyphenol-rich food, cranberry contains complex polyphenol components, so it seems difficult to attribute metabolic benefits to a particular polyphenol [62]. More and more studies have found that polyphenols are decomposed into different metabolites during metabolism [57]. There were synergistic effects between these different forms of metabolites, which had beneficial effects on lipid and glucose metabolism [56]. Microbial metabolism deserves special consideration because some polyphenol metabolites are formed through the action of gut microbiota [56,62]. Moreover, high variation in blood and urinary metabolite levels was observed in humans [63]. Sensitivity and bioavailability to polyphenols varied in individuals, and one of the reasons could be explained by the differences in the gut microbiome [64]. In terms of safety, no adverse events or side effects were reported based on the dosage of cranberry in the included studies. Anthocyanins are relatively safe as a natural pigment and are non-toxic [65]. Nonetheless, further research is required to determine the safety of long-term consumption of effective doses.

Dosage form, duration, health status, cranberry dosage, dietary intake, and physical activity of the subjects during the intervention could also influence lipid and glucose metabolism. Nonetheless, they were not strictly controlled in each study, and dietary requirements were different from each other. A future perspective may investigate the effects of cranberry on blood lipids while controlling participants’ diet intake to reduce interference.

In addition to the efficacy of cranberry on the variables mentioned above, several studies have reported additional metabolism-related markers influenced by cranberry. These studies have demonstrated that cranberry had a positive impact on C-reaction protein (CRP) [11,30], blood pressure [11,31], body weight [66], waist circumference [66], and BMI [66]. Novotny et al. assumed that consuming 480 mL of a low-caloric cranberry beverage helped to lower CRP and diastolic blood pressure (DBP) [11]. Another study concluded similarly, in terms of CRP, that a reduction in oxidative stress levels occurred in people consuming cranberry with elevated CRP concentrations [30], and the possible mechanism was that quercetin in cranberry had abilities to suppress CRP expression in liver cells [67]. One study extracted data from the National Health and Nutrition Examination Survey (NHANES) 2005–2008 and found that cranberry consumers had significantly lower weight, BMI, and waist circumferences than non-cranberry consumers [66]. However, more high-quality RCTs are required to confirm these perspectives.

This meta-analysis had several strengths. This study updated the latest research evidence based on a comprehensive literature search, and 16 studies were included, which was relatively sufficient. The study conducted an integrated and objective evaluation of the findings of existing research on the effects of cranberry supplementation on blood lipid and glucose. It improved the statistical efficiency of the original results. The literature included all RCTs with relatively high quality. Only two of them were at risk of blinding; other studies were not at high risk in any aspect. Nevertheless, the meta-analysis had some limitations that should be noted. According to Egger’s test, potential publication bias was detected in terms of HDL-C. The study had potential heterogeneity on the combined effect size of HOMA-IR, which could affect the quality of the conclusion. Although we tried to find sources of heterogeneity in subgroup analyses, heterogeneity in some subgroups was not changed. Despite cranberry supplementation, dietary intake varied in different studies, and it cannot be determined whether other nutritional factors had an impact on lipid profiles and blood glucose indexes.

## 5. Conclusions

This meta-analysis demonstrated that cranberry consumption improves blood TC and HDL-C ratio and HOMA-IR levels. The results should be interpreted with caution due to only a few RCTs containing these two parameters. The current evidence on the efficacy of cranberry supplementation regarding other blood lipid profiles and glycemic indicator regulation is still limited. Cranberries in dried form (tablets, capsules, and powder) might have a lowering effect on fasting insulin, and it is reasonable to conduct more research on this form of supplementation in the future. Overall, high-quality RCTs are required to solidify our findings and provide convincing evidence on cranberry supplement dosages, even the dosage of specific polyphenols. The research process needs to standardize and quantify the cranberry supplement dose while controlling factors such as dietary intake and physical activities.

## Figures and Tables

**Table 1:** Study characteristics of included studies.

Authors, Years	Country	Subjects	Type of Study	Sample Size (I/C)	Age	Intervention Group			Control Group		Intervention Time	Outcomes
						Brands	Forms	Phenolic Content	Forms	Phenolic Content		
Duthie et al., 2006 [12]	UK	healthy female	parallel	20 (11/9)	18–40	Ocean Spray, Middleborough, MA, USA	750 mL/day cranberry juice	total polyphenols: 850 mg; anthocyanins: 2.2 mg; catechins 22 mg	750 mL placebo drink	0	2 weeks	no changes
Lee et al., 2008 [10]	Taiwan	type 2 diabetes	parallel	30 (15/15)	63–68	Triarco Industries Inc., Cranbury, NJ, USA	3 capsules of cranberry extracts (500 mg/capsule)	total polyphenols: 328.5 mg *; anthocyanins: 0.08 mg *; flavonoids 3.2 mg *	3 placebo capsules	not mentioned	12 weeks	TC, LDL-C, TC/HDL-C↓
Dohadwala et al., 2011 [25]	USA	stable coronary artery disease	crossover	22 (22/22)	50–72	Ocean Spray, Middleborough, MA, USA	480 mL cranberry juice, double-strength (54% juice)	total polyphenols: 835 mg; anthocyanins: 94 mg	480 mL placebo drink	0	4 weeks	HDL-C↓
Basu et al., 2011 [24]	USA	female with metabolic syndrome	parallel	31 (15/16)	44–60	Ocean Spray, Middleborough, MA, USA	480 mL cranberry juice	total polyphenols: 458 mg; anthocyanins: 24.8 mg	480 mL placebo juice	0	8 weeks	no changes
Shidfar et al., 2011 [13]	Iran	type 2 diabetic male	parallel	58 (29/29)	45–64	Not mentioned	240 mL cranberry juice	unavailable	240 mL placebo drink	0	12 weeks	no changes
Flammer et al., 2013 [26]	USA	peripheral endothelial dysfunction and cardiovascular risk factors	parallel	69 (32/37)	27–67	Ocean Spray, Middleborough, MA, USA	460 mL cranberry juice cocktail, double-strength (54% juice)	total polyphenols: 800 mg; anthocyanins: 70 mg	460 mL placebo juice	0	4 months	no changes
Novotny et al., 2015 [11]	USA	healthy	parallel	56 (29/27)	25–65	Ocean Spray, Middleborough, MA, USA	480 mL cranberry juice	total polyphenols: 346 mg; anthocyanins: 20.6 mg; proanthocyanins: 236 mg	480 mL placebo juice	total polyphenols: 124 mg; anthocyanins: 0	8 weeks	TG, FBG, HOMA-IR↓
Javid et al., 2017 [28]	Iran	diabetes and periodontal disease	parallel	31 (9/12)	35–67	Takdaneh Industry & Cultivate Company, Tehran, Iran	400 mL cranberry juice	total polyphenols: 390 mg; anthocyanins: 16 mg; proanthocyanins: 214 mg	none	none	8 weeks	no changes
Paquette et al., 2017 [27]	Canada	overweight/obese with insulin resistant	parallel	41 (20/21)	40–70	Nutra-Canada, Champlain, QC, Canada	120 mL strawberry and cranberry polyphenols (SCP) beverage	total polyphenols: 333 mg; anthocyanins: unavailable	120 mL SCP-free beverage	total polyphenols: around 10 mg	6 weeks	no changes
Hsia et al., 2020 [30]	USA	obese with elevated fasting glucose/impaired glucose tolerance	parallel	35 (18/17)	31–63	Ocean Spray, Middleborough, MA, USA	450 mL low-calorie cranberry beverage	total polyphenols: 158 mg; anthocyanins: 6.75 mg; proanthocyanins: 143 mg	450 mL placebo juice	0	8 weeks	TG↓ when CRP > 4 mg/L
Hormoznejad et al., 2020 [29]	Iran	non-alcoholic fatty liver	parallel	41 (20/21)	30–55	Shari Company, Tehran, Iran	2 Cranberry tablets (equal to 26 g dried cranberry fruit)	total polyphenols and anthocyanins: unavailable proanthocyanins: 72 mg	Placebo tablets	0	12 weeks	fasting insulin↓, HOMA-IR↓ in cranberry group; HDL-C↑ in placebo group
Richter et al., 2021 [31]	USA	overweight/obese and elevated brachial blood pressure	crossover	40 (40/40)	30–65	Ocean Spray, Middleborough, MA, USA	500 mL cranberry juice	total polyphenols: 320 mg; anthocyanins: 4.5 mg; phenolic acids: 68 mg; flavonols: 17 mg	500 mL placebo drink	0	8–12 weeks	no changes
Shirazi et al., 2021 [14]	Iran	patients with non-alcoholic fatty liver	parallel	94 (46/48)	32–55	Shari Company, Tehran, Iran	144 mg cranberry capsule (equal to 13 g dried cranberry fruit)	total polyphenols and anthocyanins: unavailable	placebo capsule	0	6 months	TC, TG, insulin, HOMA-IR↓
Heiss et al., 2022 [33]	UK	healthy male	parallel	44 (22/22)	18–45	Cranberry Institute, Carver, MA, USA	9 g cranberry powder	total polyphenols: 525 mg; anthocyanins: 54 mg; proanthocyanidins: 374.2 mg; phenolic acids: 17 mg	placebo powder	0	1 month	No changes
Flanagan et al., 2022 [32]	UK	healthy	parallel	60 (29/31)	50–80	Cranberry Institute, Carver, MA, USA	9 g cranberry powder	total polyphenols: 588 mg; anthocyanins: 59 mg	placebo powder	0	12 weeks	LDL-C↓
Rahn et al., 2023 [15]	Germany	healthy male	parallel	36 (18/18)	22–27	Eckes-Granini Group GmbH, Nieder-Olm, Germany	750 mL drinks (51% chokeberry, cranberry, and pomegranate)	total polyphenols: 2250 mg; anthocyanins: 552 mg	750 mL placebo drink	0	8 weeks	TG↑ in placebo group

Abbreviations: TG: triacylglycerol; TC: total cholesterol; LDL-C: low-density lipoprotein cholesterol; HDL-C: high-density lipoprotein cholesterol; HOMA-IR: homeostatic model of insulin resistance. * Dosage not provided in original article; data were obtained or transferred from related articles. “↑” stand for increase; “↓” stand for decrease.

**Table 2:** The meta-analysis of all indicators.

Indicators	Studies Numbers	I^2^	*p*_heterogeneity_	MD	95% CI	Z Values	*p*_effect_	Egger’s Test
TC	14	48%	0.02	−0.11	(−0.26, 0.03)	1.57	0.12	−0.59, 0.567
HDL	14	0%	0.45	−0.02	(−0.05, 0.01)	1.16	0.25	−2.87, 0.015
LDL	13	38%	0.08	−0.10	(−0.20, 0.01)	1.79	0.07	0.46, 0.657
TG	15	35%	0.09	0.06	(0.00, 0.12)	2.00	0.05	−0.90, 0.385
TC/HDL-C	4	0%	0.43	−0.24	(−0.45, −0.04)	2.30	0.02	/
FBG	13	55%	0.009	−0.09	(−0.23, 0.04)	1.38	0.17	−0.45, 0.660
HbA1c	3	0%	0.61	−0.16	(−0.38, 0.05)	1.50	0.13	/
Insulin	6	81%	<0.001	−1.31	(−2.85, 0.22)	1.68	0.09	/
HOMA-IR	5	63%	0.03	−0.59	(−1.05, −0.14)	2.56	0.01	/

Abbreviations: MD: mean difference; TC: total cholesterol; TG: triglyceride; HDL: high-density lipoprotein; LDL: low-density lipoprotein; FBG: fasting blood glucose; HbA1c: glycated hemoglobin; HOMA-IR: homeostasis model assessment of insulin resistance. *p*_heterogeneity_ for heterogeneity test. *p*_effect_ for combined effect.

## Data Availability

Data are contained within the article and Supplementary Materials.

## Supplementary Materials

The following supporting information can be downloaded at: https://www.mdpi.com/article/10.3390/nu16060782/s1, Figure S1: Funnel plot to evaluate the publication bias for TC. Figure S2: Funnel plot to evaluate the publication bias for HDL-C. Figure S3: Funnel plot to evaluate the publication bias for LDL-C. Figure S4: Funnel plot to evaluate the publication bias for TG. Figure S5: Funnel plot to evaluate the publication bias for FBG. Figure S6: Subgroup analysis of the effect of dosage form on TC. Figure S7: Subgroup analysis of the effect of dosage form on HDL-C. Figure S8: Subgroup analysis of the effect of dosage form on LDL-C. Figure S9: Subgroup analysis of the effect of dosage form on TG. Figure S10: Subgroup analysis of the effect of dosage form on FBG. Figure S11: Subgroup analysis of the effect of dosage form on fasting insulin. Figure S12: Subgroup analysis of the effect of intervention duration on TC. Figure S13: Subgroup analysis of the effect of intervention duration on HDL-C. Figure S14: Subgroup analysis of the effect of intervention duration on LDL-C. Figure S15: Subgroup analysis of the effect of intervention duration on TG. Figure S16: Subgroup analysis of the effect of intervention duration on FBG. Figure S17: Subgroup analysis of the effect of intervention duration on fasting insulin. Figure S18: Subgroup analysis of the effect of health conditions on TC. Figure S19: Subgroup analysis of the effect of health conditions on HDL-C. Figure S20: Subgroup analysis of the effect of health conditions on LDL-C. Figure S21: Subgroup analysis of the effect of health conditions on TG. Figure S22: Subgroup analysis of the effect of health conditions on FBG. Figure S23: Subgroup analysis of the effect of health conditions on fasting insulin. Figure S24: Subgroup analysis of the effect of the dosage of total polyphenols on TC. Figure S25: Subgroup analysis of the effect of the dosage of total polyphenols on HDL-C. Figure S26: Subgroup analysis of the effect of the dosage of total polyphenols on LDL-C. Figure S27: Subgroup analysis of the effect of the dosage of total polyphenols on TG. Figure S28: Subgroup analysis of the effect of the dosage of total polyphenols on FBG. Figure S29: Subgroup analysis of the effect of the dosage of total polyphenols on fasting insulin. Figure S30: Subgroup analysis of the effect of the dosage of anthocyanins on TC. Figure S31: Subgroup analysis of the effect of the dosage of anthocyanins on HDL-C. Figure S32: Subgroup analysis of the effect of the dosage of anthocyanins on LDL-C. Figure S33: Subgroup analysis of the effect of the dosage of anthocyanins on TG. Figure S34: Subgroup analysis of the effect of the dosage of anthocyanins on FBG. Figure S35: Subgroup analysis of the effect of the dosage of anthocyanins on fasting insulin.

## Appendix A

PubMed: (Metabolic OR Metabolism OR lipoprotein OR cholesterol OR triglyceride OR lipid profile OR TG OR TC OR LDL OR VLDL OR blood lipid OR APO OR apolipoprotein) AND (cranberry OR Vaccinium macrocarpon OR Vaccinium microcarpum OR Vaccinium oxycoccus).

Web of Science: TS = (Metabolic OR Metabolism OR lipoprotein OR cholesterol OR triglyceride OR lipid profile OR TG OR TC OR LDL OR VLDL OR blood lipid OR APO OR apolipoprotein) AND TS = (cranberry OR Vaccinium macrocarpon OR Vaccinium microcarpum OR Vaccinium oxycoccus).

Scopus: (TITLE-ABS-KEY (metabolic OR metabolism OR lipoproteins OR cholesterol OR triglyceride OR lipid OR tg OR tc OR ldl OR vldl OR apo OR apolipoprotein) AND TITLE-ABS-KEY (cranberry OR (vaccinium AND macrocarpon) OR (vaccinium AND microcarpum) OR (vaccinium AND oxycoccus))).
