# Supplementary material for: The Effects of Cranberry Consumption on Glycemic and Lipid Profiles in Humans: A Systematic Review and Meta-Analysis of Randomized Controlled Trials
Source: Nutrients. 2024 Mar 9;16(6):782. doi: 10.3390/nu16060782 (PMC10974925; doi:10.3390/nu16060782)

Study or Subgroup	Experimental Mean	Experimental SD	Experimental Total	Control Mean	Control SD	Control Total	Weight	Mean Difference IV, Random, 95% CI	Year
**2.1.1 dried form (capsule, powder, tablets**									
Lee 2008	−0.4	0.39	15	0.3	0.39	15	11.3%	−0.70 [−0.98, −0.42]	2008
Hormoznejad 2020	−0.04	1.17	20	−0.22	0.74	21	4.4%	0.18 [−0.42, 0.78]	2020
Shirazi 2021	−0.54	1.65	46	−0.13	0.32	48	6.0%	−0.41 [−0.90, 0.08]	2021
Flanagan 2022	−0.2	1.15	29	−0.1	1.05	31	4.9%	−0.10 [−0.66, 0.46]	2022
Heiss 2022	−0.13	0.51	22	0.01	1.69	22	3.1%	−0.14 [−0.88, 0.60]	2022
**Subtotal (95% CI)**			**132**			**137**	**29.7%**	**−0.30 [−0.64, 0.05]**	
Heterogeneity: $Tau^2 = 0.09$; $Chi^2 = 9.47$, df = 4 (P = 0.05); $I^2 = 58\%$									
Test for overall effect: Z = 1.70 (P = 0.09)									
**2.1.2 juice form**									
Duthie 2006	−0.01	0.65	11	−0.03	0.75	9	4.1%	0.02 [−0.60, 0.64]	2006
Basu 2011	−0.14	0.85	15	0.13	0.81	16	4.6%	−0.27 [−0.86, 0.32]	2011
Dohadwala 2011	−0.05	1.02	22	0	1.07	22	4.2%	−0.05 [−0.67, 0.57]	2011
Flammer 2013	0.1	0.16	32	0.14	0.18	37	18.7%	−0.04 [−0.12, 0.04]	2013
Novotny 2015	0.08	0.61	29	0	0.73	27	8.9%	0.08 [−0.27, 0.43]	2015
Javid 2017	−0.01	1.07	9	−0.2	0.83	12	2.5%	0.19 [−0.65, 1.03]	2017
Paquette 2017	−0.1	0.81	20	0.08	0.97	21	5.1%	−0.18 [−0.73, 0.37]	2017
Richter 2021	−0.03	0.85	40	−0.03	0.85	40	8.4%	0.00 [−0.37, 0.37]	2021
Rahn 2023	−0.01	0.24	18	−0.04	0.39	18	13.8%	0.03 [−0.18, 0.24]	2023
**Subtotal (95% CI)**			**196**			**202**	**70.3%**	**−0.03 [−0.10, 0.04]**	
Heterogeneity: $Tau^2 = 0.00$; $Chi^2 = 1.99$, df = 8 (P = 0.98); $I^2 = 0\%$									
Test for overall effect: Z = 0.83 (P = 0.40)									
**Total (95% CI)**			**328**			**339**	**100.0%**	**−0.12 [−0.26, 0.02]**	
Heterogeneity: $Tau^2 = 0.03$; $Chi^2 = 25.43$, df = 13 (P = 0.02); $I^2 = 49\%$									
Test for overall effect: Z = 1.64 (P = 0.10)									
Test for subgroup differences: $Chi^2 = 2.24$, df = 1 (P = 0.13), $I^2 = 55.4\%$									

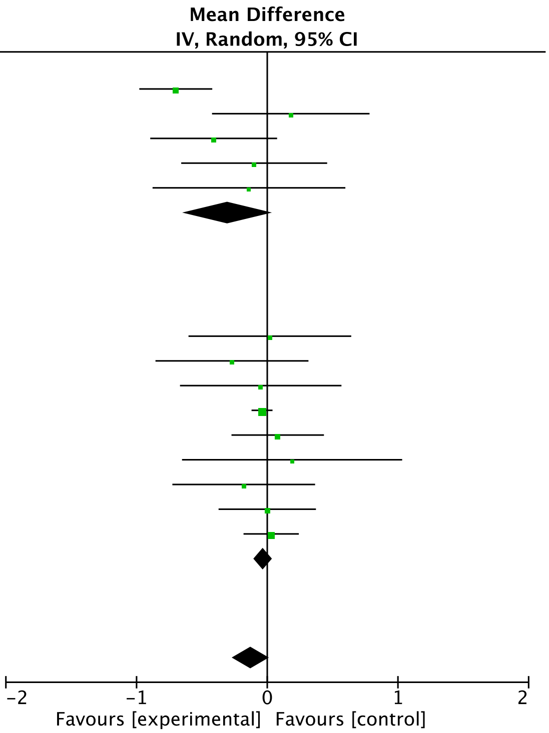

Supplement: Supplementary file 1 [file nutrients-16-00782-s001.zip › Supplementary Figure 6. Subgroup analysis of the effect of dosage form on TC.pdf]
