# Supplementary material for: The Effects of Cranberry Consumption on Glycemic and Lipid Profiles in Humans: A Systematic Review and Meta-Analysis of Randomized Controlled Trials
Source: Nutrients. 2024 Mar 9;16(6):782. doi: 10.3390/nu16060782 (PMC10974925; doi:10.3390/nu16060782)

Study or Subgroup	Experimental Mean	Experimental SD	Experimental Total	Control Mean	Control SD	Control Total	Weight	Mean Difference IV, Random, 95% CI	Year
**2.3.1 dried form (capsules, powder, tablets)**									
Lee 2008	−0.4	0.39	15	0.2	0.39	15	12.4%	−0.60 [−0.88, −0.32]	2008
Hormoznejad 2020	0.09	1.13	20	−0.23	0.84	21	4.5%	0.32 [−0.29, 0.93]	2020
Shirazi 2021	−0.26	0.53	46	−0.21	0.51	48	15.4%	−0.05 [−0.26, 0.16]	2021
Flanagan 2022	−0.3	1	29	−0.1	1.08	31	5.7%	−0.20 [−0.73, 0.33]	2022
Heiss 2022	−0.05	0.56	22	0.04	1.53	22	3.8%	−0.09 [−0.77, 0.59]	2022
**Subtotal (95% CI)**			**132**			**137**	**41.8%**	**−0.17 [−0.49, 0.15]**	
Heterogeneity: Tau^2 = 0.08; Chi^2 = 12.72, df = 4 (P = 0.01); I^2 = 69%									
Test for overall effect: Z = 1.04 (P = 0.30)									
**2.3.2 juice form**									
Duthie 2006	−0.38	0.65	11	−0.2	0.69	9	4.8%	−0.18 [−0.77, 0.41]	2006
Basu 2011	−0.13	0.67	15	0.1	0.68	16	6.6%	−0.23 [−0.71, 0.25]	2011
Dohadwala 2011	0	0.85	22	−0.03	0.89	22	5.9%	0.03 [−0.48, 0.54]	2011
Novotny 2015	0.1	0.51	29	0.03	0.5	27	13.0%	0.07 [−0.19, 0.33]	2015
Javid 2017	0.08	1.39	9	−0.25	0.72	12	1.9%	0.33 [−0.67, 1.33]	2017
Paquette 2017	−0.01	0.76	20	0.17	0.74	21	6.9%	−0.18 [−0.64, 0.28]	2017
Richter 2021	0	0.71	40	−0.08	0.71	40	11.1%	0.08 [−0.23, 0.39]	2021
Rahn 2023	−0.03	0.5	18	−0.08	0.75	18	7.9%	0.05 [−0.37, 0.47]	2023
**Subtotal (95% CI)**			**164**			**165**	**58.2%**	**0.00 [−0.14, 0.15]**	
Heterogeneity: Tau^2 = 0.00; Chi^2 = 2.85, df = 7 (P = 0.90); I^2 = 0%									
Test for overall effect: Z = 0.05 (P = 0.96)									
**Total (95% CI)**			**296**			**302**	**100.0%**	**−0.09 [−0.23, 0.06]**	
Heterogeneity: Tau^2 = 0.02; Chi^2 = 19.29, df = 12 (P = 0.08); I^2 = 38%									
Test for overall effect: Z = 1.19 (P = 0.23)									
Test for subgroup differences: Chi^2 = 0.94, df = 1 (P = 0.33), I^2 = 0%									

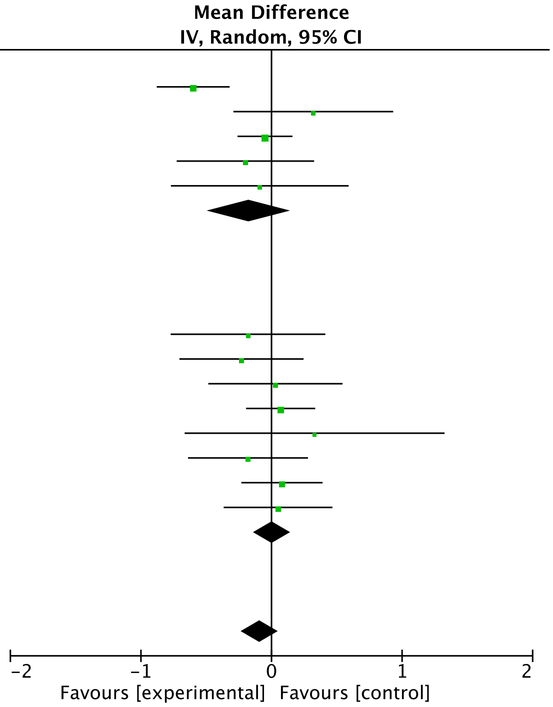

Supplement: Supplementary file 1 [file nutrients-16-00782-s001.zip › Supplementary Figure 8. Subgroup analysis of the effect of dosage form on LDL-C.pdf]
