# Supplementary material for: The Effects of Cranberry Consumption on Glycemic and Lipid Profiles in Humans: A Systematic Review and Meta-Analysis of Randomized Controlled Trials
Source: Nutrients. 2024 Mar 9;16(6):782. doi: 10.3390/nu16060782 (PMC10974925; doi:10.3390/nu16060782)
Supplement: Supplementary file 1 [file nutrients-16-00782-s001.zip › Supplementary materials-pdf version.pdf]

Supplementary materials

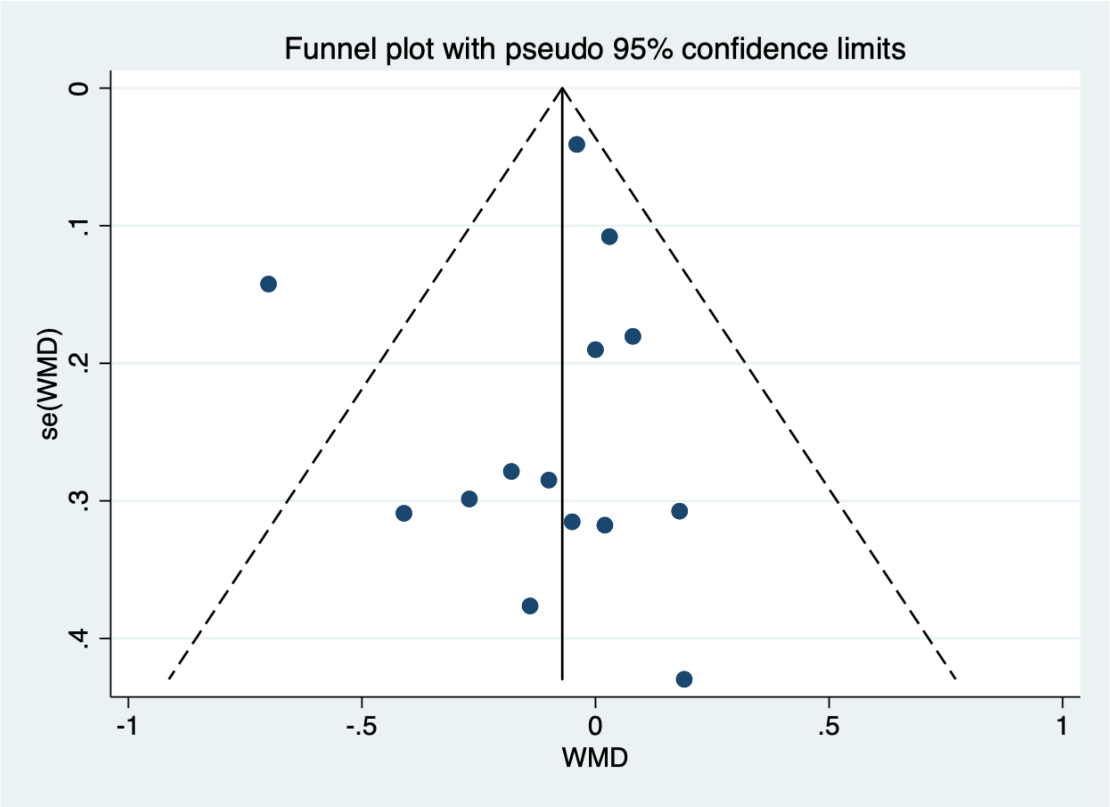

Supplementary Figure 1. Funnel plot to evaluate the publication bias for TC.

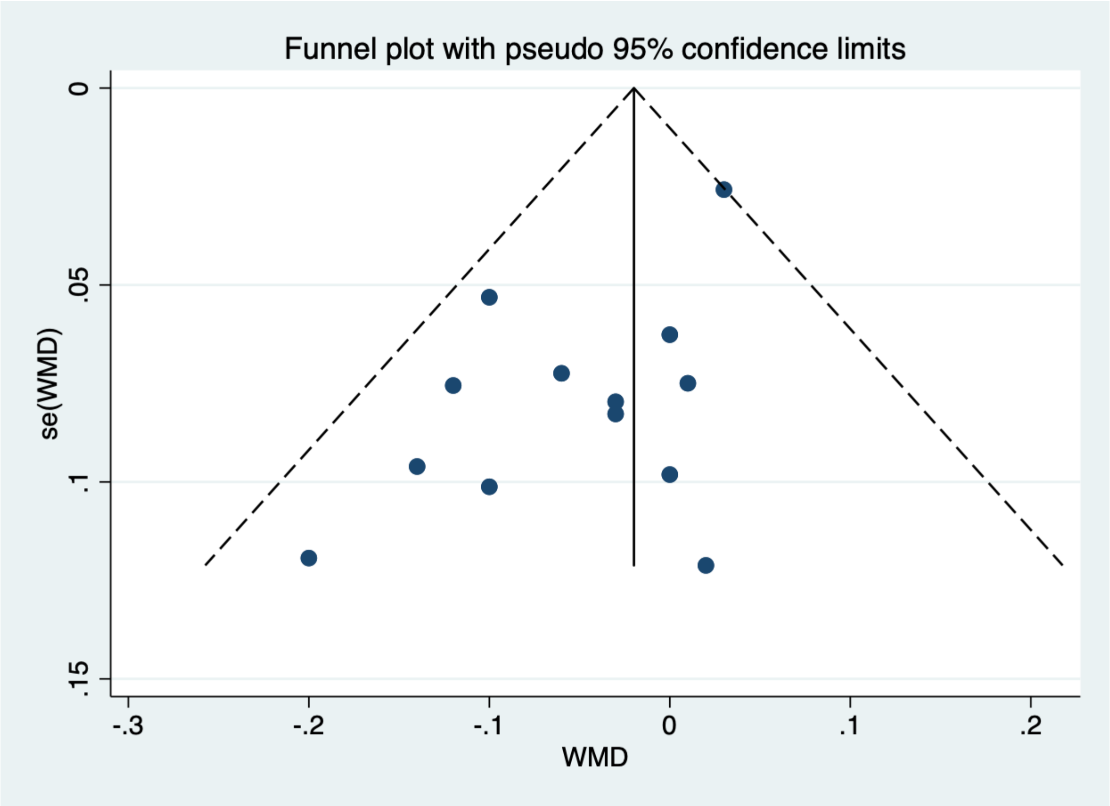

Supplementary Figure 2. Funnel plot to evaluate the publication bias for HDL-C.

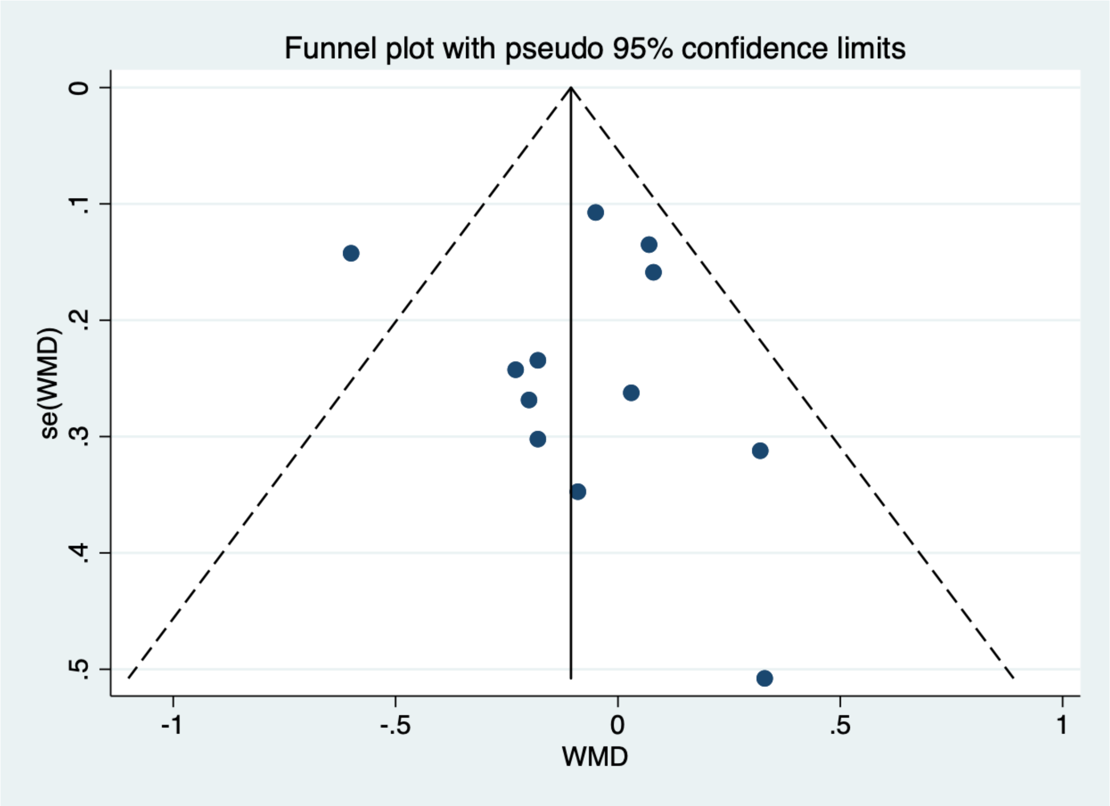

Supplementary Figure 3. Funnel plot to evaluate the publication bias for LDL-C.

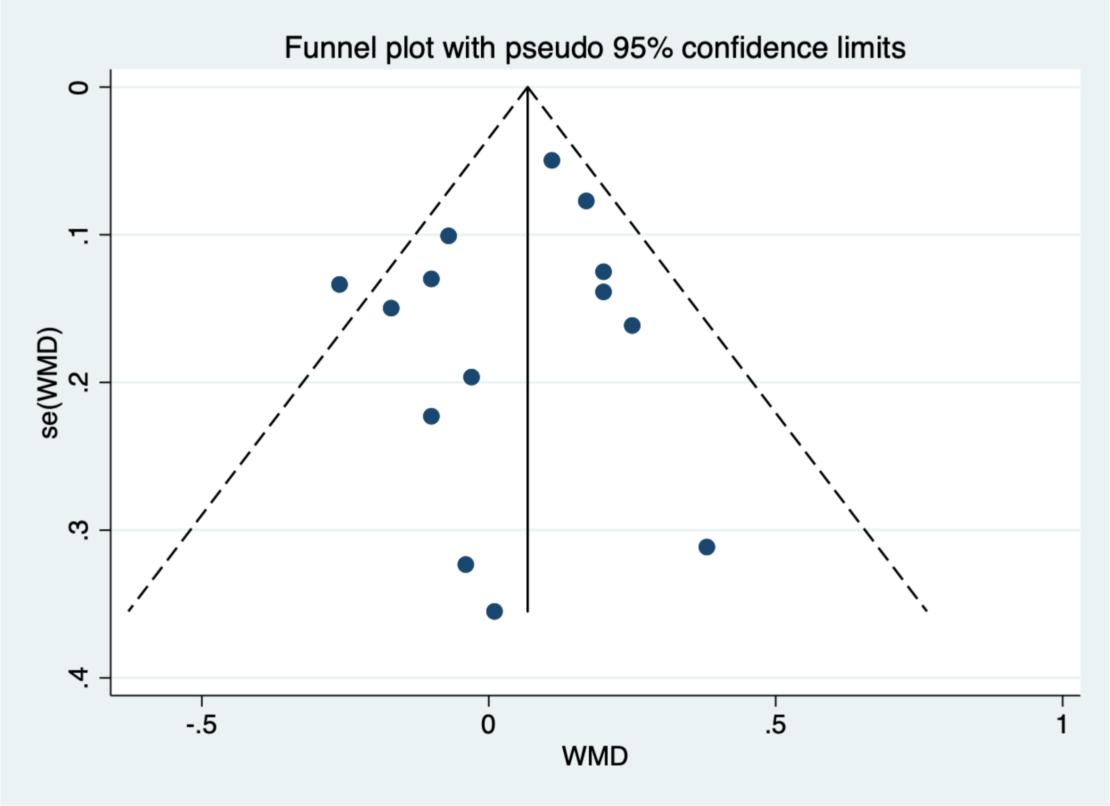

Supplementary Figure 4. Funnel plot to evaluate the publication bias for TG.

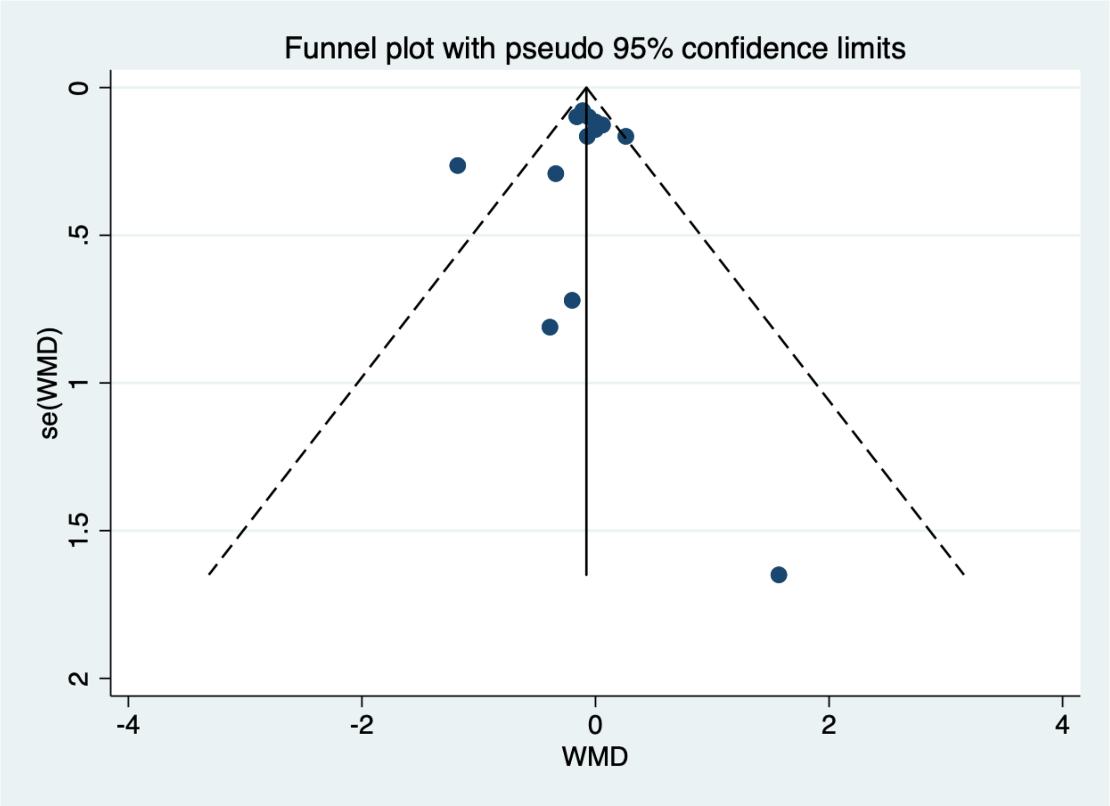

Supplementary Figure 5. Funnel plot to evaluate the publication bias for FBG.

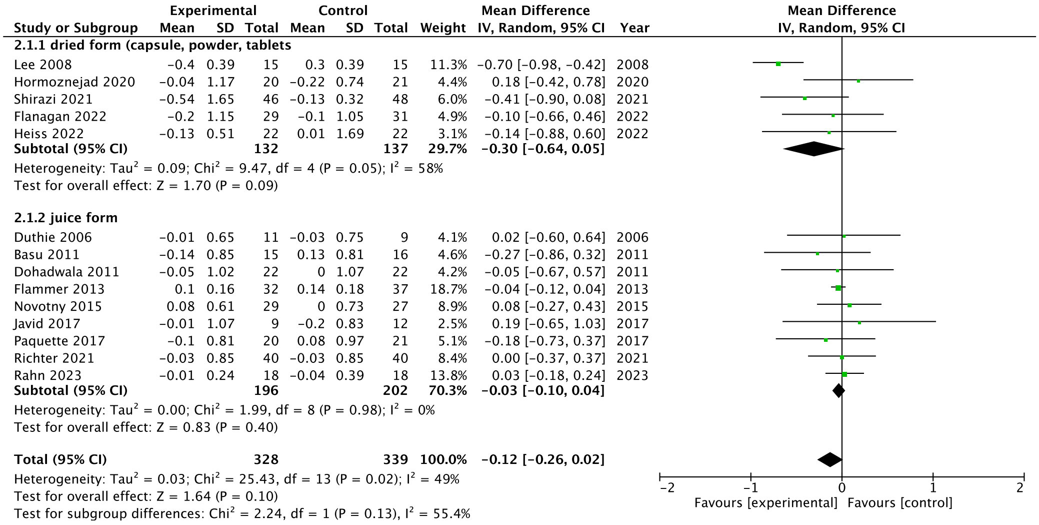

Supplementary Figure 6. Subgroup analysis of the effect of dosage form on TC.

Study or Subgroup	Experimental Mean	SD	Total	Control Mean	SD	Total	Weight	Mean Difference IV, Random, 95% CI	Year
**2.2.1 dried form (capsules, powder, tablets)**									
Lee 2008	0	0.39	15	0.1	0.04	15	2.8%	−0.10 [−0.30, 0.10]	2008
Hormoznejad 2020	−0.01	0.17	20	0.09	0.17	21	10.1%	−0.10 [−0.20, 0.00]	2020
Shirazi 2021	0.12	0.12	46	0.09	0.13	48	42.9%	0.03 [−0.02, 0.08]	2021
Flanagan 2022	0	0.36	29	0	0.4	31	3.0%	0.00 [−0.19, 0.19]	2022
Heiss 2022	−0.13	0.18	22	0.07	0.53	22	2.0%	−0.20 [−0.43, 0.03]	2022
**Subtotal (95% CI)**			**132**			**137**	**60.8%**	**−0.05 [−0.14, 0.04]**	
Heterogeneity: $Tau^2 = 0.00$; $Chi^2 = 8.59$, df = 4 (P = 0.07); $I^2 = 53\%$									
Test for overall effect: Z = 1.11 (P = 0.27)									
**2.2.2 juice form**									
Duthie 2006	0	0.32	11	−0.02	0.22	9	1.9%	0.02 [−0.22, 0.26]	2006
Dohadwala 2011	−0.03	0.25	22	0.03	0.23	22	5.4%	−0.06 [−0.20, 0.08]	2011
Basu 2011	−0.04	0.21	15	−0.01	0.25	16	4.2%	−0.03 [−0.19, 0.13]	2011
Flammer 2013	0	0.21	32	0.12	0.4	37	5.0%	−0.12 [−0.27, 0.03]	2013
Novotny 2015	0.04	0.23	29	0.03	0.32	27	5.1%	0.01 [−0.14, 0.16]	2015
Paquette 2017	0.01	0.25	20	0.04	0.26	21	4.5%	−0.03 [−0.19, 0.13]	2017
Javid 2017	0.03	0.18	9	0.17	0.26	12	3.1%	−0.14 [−0.33, 0.05]	2017
Richter 2021	0	0.28	40	0	0.28	40	7.3%	0.00 [−0.12, 0.12]	2021
Rahn 2023	−0.12	0.28	18	−0.11	0.33	18	2.7%	−0.01 [−0.21, 0.19]	2023
**Subtotal (95% CI)**			**196**			**202**	**39.2%**	**−0.04 [−0.09, 0.01]**	
Heterogeneity: $Tau^2 = 0.00$; $Chi^2 = 3.50$, df = 8 (P = 0.90); $I^2 = 0\%$									
Test for overall effect: Z = 1.47 (P = 0.14)									
**Total (95% CI)**			**328**			**339**	**100.0%**	**−0.02 [−0.05, 0.01]**	
Heterogeneity: $Tau^2 = 0.00$; $Chi^2 = 13.00$, df = 13 (P = 0.45); $I^2 = 0\%$									
Test for overall effect: Z = 1.16 (P = 0.25)									
Test for subgroup differences: $Chi^2 = 0.03$, df = 1 (P = 0.86), $I^2 = 0\%$									

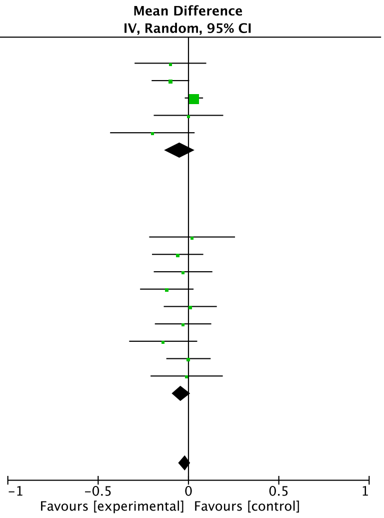

Supplementary Figure 7. Subgroup analysis of the effect of dosage form on HDL-C.

Study or Subgroup	Experimental Mean	SD	Total	Control Mean	SD	Total	Weight	Mean Difference IV, Random, 95% CI	Year
**2.3.1 dried form (capsules, powder, tablets)**									
Lee 2008	−0.4	0.39	15	0.2	0.39	15	12.4%	−0.60 [−0.88, −0.32]	2008
Hormoznejad 2020	0.09	1.13	20	−0.23	0.84	21	4.5%	0.32 [−0.29, 0.93]	2020
Shirazi 2021	−0.26	0.53	46	−0.21	0.51	48	15.4%	−0.05 [−0.26, 0.16]	2021
Flanagan 2022	−0.3	1	29	−0.1	1.08	31	5.7%	−0.20 [−0.73, 0.33]	2022
Heiss 2022	−0.05	0.56	22	0.04	1.53	22	3.8%	−0.09 [−0.77, 0.59]	2022
**Subtotal (95% CI)**			**132**			**137**	**41.8%**	**−0.17 [−0.49, 0.15]**	
Heterogeneity: $Tau^2 = 0.08$; $Chi^2 = 12.72$, df = 4 (P = 0.01); $I^2 = 69\%$									
Test for overall effect: Z = 1.04 (P = 0.30)									
**2.3.2 juice form**									
Duthie 2006	−0.38	0.65	11	−0.2	0.69	9	4.8%	−0.18 [−0.77, 0.41]	2006
Basu 2011	−0.13	0.67	15	0.1	0.68	16	6.6%	−0.23 [−0.71, 0.25]	2011
Dohadwala 2011	0	0.85	22	−0.03	0.89	22	5.9%	0.03 [−0.48, 0.54]	2011
Novotny 2015	0.1	0.51	29	0.03	0.5	27	13.0%	0.07 [−0.19, 0.33]	2015
Javid 2017	0.08	1.39	9	−0.25	0.72	12	1.9%	0.33 [−0.67, 1.33]	2017
Paquette 2017	−0.01	0.76	20	0.17	0.74	21	6.9%	−0.18 [−0.64, 0.28]	2017
Richter 2021	0	0.71	40	−0.08	0.71	40	11.1%	0.08 [−0.23, 0.39]	2021
Rahn 2023	−0.03	0.5	18	−0.08	0.75	18	7.9%	0.05 [−0.37, 0.47]	2023
**Subtotal (95% CI)**			**164**			**165**	**58.2%**	**0.00 [−0.14, 0.15]**	
Heterogeneity: $Tau^2 = 0.00$; $Chi^2 = 2.85$, df = 7 (P = 0.90); $I^2 = 0\%$									
Test for overall effect: Z = 0.05 (P = 0.96)									
**Total (95% CI)**			**296**			**302**	**100.0%**	**−0.09 [−0.23, 0.06]**	
Heterogeneity: $Tau^2 = 0.02$; $Chi^2 = 19.29$, df = 12 (P = 0.08); $I^2 = 38\%$									
Test for overall effect: Z = 1.19 (P = 0.23)									
Test for subgroup differences: $Chi^2 = 0.94$, df = 1 (P = 0.33), $I^2 = 0\%$									

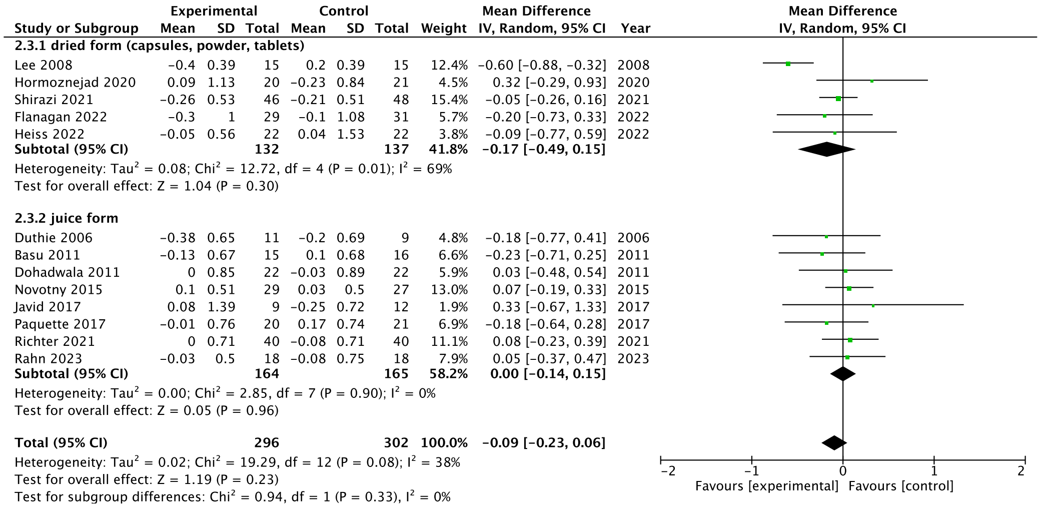

Supplementary Figure 8. Subgroup analysis of the effect of dosage form on LDL-C.

Study or Subgroup	Experimental Mean	SD	Total	Control Mean	SD	Total	Weight	Mean Difference IV, Fixed, 95% CI	Year
**2.4.1 dried form (capsules, powder, tablets)**									
Lee 2008	−0.1	0.77	15	0	0.39	15	1.8%	−0.10 [−0.54, 0.34]	2008
Hormoznejad 2020	−0.17	0.39	20	−0.55	1.37	21	0.9%	0.38 [−0.23, 0.99]	2020
Shirazi 2021	−0.09	0.74	46	0.08	0.71	48	4.1%	−0.17 [−0.46, 0.12]	2021
Flanagan 2022	0.1	0.4	29	−0.1	0.56	31	5.8%	0.20 [−0.05, 0.45]	2022
Heiss 2022	0.13	0.46	22	−0.07	0.46	22	4.7%	0.20 [−0.07, 0.47]	2022
**Subtotal (95% CI)**			**132**			**137**	**17.4%**	**0.09 [−0.05, 0.23]**	
Heterogeneity: $Chi^2 = 6.01$, df = 4 (P = 0.20); $I^2 = 33\%$									
Test for overall effect: Z = 1.27 (P = 0.21)									
**2.4.2 juice form**									
Duthie 2006	0.24	0.37	11	−0.01	0.35	9	3.5%	0.25 [−0.07, 0.57]	2006
Dohadwala 2011	−0.02	0.61	22	0.01	0.69	22	2.4%	−0.03 [−0.41, 0.35]	2011
Basu 2011	0.07	0.16	15	−0.04	0.11	16	37.0%	0.11 [0.01, 0.21]	2011
Flammer 2013	0.07	0.31	32	−0.1	0.33	37	15.3%	0.17 [0.02, 0.32]	2013
Novotny 2015	−0.13	0.47	29	−0.03	0.5	27	5.4%	−0.10 [−0.35, 0.15]	2015
Javid 2017	−0.24	1.01	9	−0.25	0.39	12	0.7%	0.01 [−0.69, 0.71]	2017
Paquette 2017	−0.21	1.01	20	−0.17	1.06	21	0.9%	−0.04 [−0.67, 0.59]	2017
Hsia 2020	−0.16	0.42	18	0.1	0.37	17	5.1%	−0.26 [−0.52, 0.00]	2020
Richter 2021	−0.01	0.43	40	0.06	0.47	40	9.0%	−0.07 [−0.27, 0.13]	2021
Rahn 2023	0.09	0.56	18	0.24	0.42	18	3.3%	−0.15 [−0.47, 0.17]	2023
**Subtotal (95% CI)**			**214**			**219**	**82.6%**	**0.05 [−0.01, 0.12]**	
Heterogeneity: $Chi^2 = 15.27$, df = 9 (P = 0.08); $I^2 = 41\%$									
Test for overall effect: Z = 1.62 (P = 0.10)									
**Total (95% CI)**			**346**			**356**	**100.0%**	**0.06 [0.00, 0.12]**	
Heterogeneity: $Chi^2 = 21.50$, df = 14 (P = 0.09); $I^2 = 35\%$									
Test for overall effect: Z = 2.00 (P = 0.05)									
Test for subgroup differences: $Chi^2 = 0.22$, df = 1 (P = 0.64), $I^2 = 0\%$									

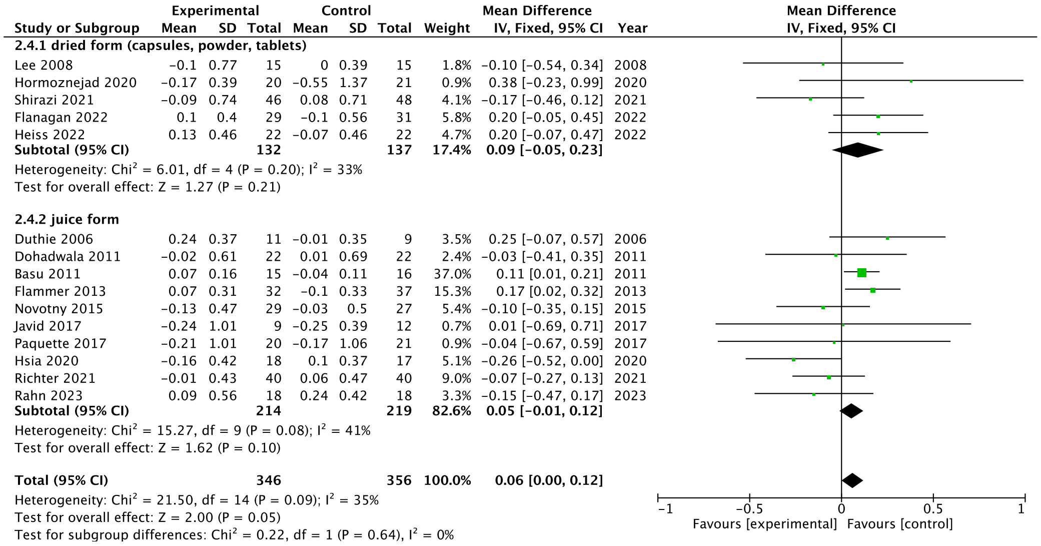

Supplementary Figure 9. Subgroup analysis of the effect of dosage form on TG.

Study or Subgroup	Experimental Mean	Experimental SD	Experimental Total	Control Mean	Control SD	Control Total	Weight	Mean Difference IV, Random, 95% CI	Year
**2.5.1 dried form (capsules, powder, tablets)**									
Lee 2008	−0.6	2.32	15	−0.4	1.55	15	0.8%	−0.20 [−1.61, 1.21]	2008
Hormoznejad 2020	−0.3	0.78	20	0.04	1.07	21	4.1%	−0.34 [−0.91, 0.23]	2020
Shirazi 2021	−0.36	0.49	46	−0.3	0.47	48	12.8%	−0.06 [−0.25, 0.13]	2021
Flanagan 2022	−0.1	0.4	29	−0.1	0.5	31	11.6%	0.00 [−0.23, 0.23]	2022
Heiss 2022	0.01	0.49	22	0.08	0.6	22	8.6%	−0.07 [−0.39, 0.25]	2022
**Subtotal (95% CI)**			**132**			**137**	**37.9%**	**−0.06 [−0.19, 0.07]**	
Heterogeneity: Tau² = 0.00; Chi² = 1.23, df = 4 (P = 0.87); I² = 0%									
Test for overall effect: Z = 0.87 (P = 0.38)									
**2.5.2 juice form**									
Basu 2011	0.32	0.45	15	0.06	0.47	16	8.6%	0.26 [−0.06, 0.58]	2011
Dohadwala 2011	−0.17	2.69	22	0.22	2.69	22	0.7%	−0.39 [−1.98, 1.20]	2011
Shidfar 2011	−1.09	0.73	29	0.09	1.22	29	4.8%	−1.18 [−1.70, −0.66]	2011
Novotny 2015	−0.1	0.38	29	0.06	0.36	27	12.8%	−0.16 [−0.35, 0.03]	2015
Javid 2017	−0.17	3.85	9	−1.74	3.59	12	0.2%	1.57 [−1.66, 4.80]	2017
Paquette 2017	0.1	0.45	20	0.1	0.46	21	9.9%	0.00 [−0.28, 0.28]	2017
Hsia 2020	0.04	0.38	18	−0.02	0.37	17	10.9%	0.06 [−0.19, 0.31]	2020
Richter 2021	0	0.35	40	0.11	0.35	40	14.3%	−0.11 [−0.26, 0.04]	2021
**Subtotal (95% CI)**			**182**			**184**	**62.1%**	**−0.12 [−0.34, 0.10]**	
Heterogeneity: Tau² = 0.06; Chi² = 25.05, df = 7 (P = 0.0007); I² = 72%									
Test for overall effect: Z = 1.08 (P = 0.28)									
**Total (95% CI)**			**314**			**321**	**100.0%**	**−0.09 [−0.23, 0.04]**	
Heterogeneity: Tau² = 0.03; Chi² = 26.41, df = 12 (P = 0.009); I² = 55%									
Test for overall effect: Z = 1.38 (P = 0.17)									
Test for subgroup differences: Chi² = 0.23, df = 1 (P = 0.63), I² = 0%									

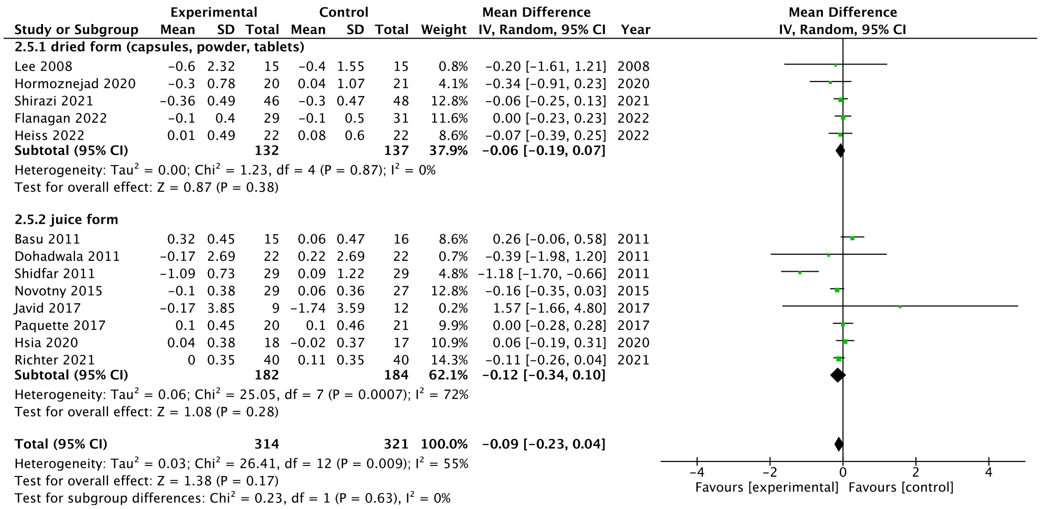

Supplementary Figure 10. Subgroup analysis of the effect of dosage form on FBG.

Study or Subgroup	Experimental Mean	Experimental SD	Experimental Total	Control Mean	Control SD	Control Total	Weight	Mean Difference IV, Random, 95% CI	Year
**2.6.1 dried form (capsules, powder, tablets)**									
Lee 2008	−0.1	3.1	15	0.4	3.1	15	15.5%	−0.50 [−2.72, 1.72]	2008
Hormoznejad 2020	−2.35	1.59	20	−0.85	1.62	21	20.9%	−1.50 [−2.48, −0.52]	2020
Shirazi 2021	−4.7	2.72	46	−0.59	2.98	48	20.2%	−4.11 [−5.26, −2.96]	2021
**Subtotal (95% CI)**			**81**			**84**	**56.6%**	**−2.16 [−4.24, −0.07]**	
Heterogeneity: Tau² = 2.83; Chi² = 14.49, df = 2 (P = 0.0007); I² = 86%									
Test for overall effect: Z = 2.02 (P = 0.04)									
**2.6.2 juice form**									
Dohadwala 2011	−1	8	22	0	7.55	22	7.5%	−1.00 [−5.60, 3.60]	2011
Novotny 2015	−0.03	3.42	29	0.18	3.72	27	17.0%	−0.21 [−2.09, 1.67]	2015
Richter 2021	0.2	3.35	40	0.1	3.29	40	18.9%	0.10 [−1.36, 1.56]	2021
**Subtotal (95% CI)**			**91**			**89**	**43.4%**	**−0.07 [−1.19, 1.04]**	
Heterogeneity: Tau² = 0.00; Chi² = 0.23, df = 2 (P = 0.89); I² = 0%									
Test for overall effect: Z = 0.13 (P = 0.90)									
**Total (95% CI)**			**172**			**173**	**100.0%**	**−1.31 [−2.85, 0.22]**	
Heterogeneity: Tau² = 2.68; Chi² = 26.46, df = 5 (P < 0.0001); I² = 81%									
Test for overall effect: Z = 1.68 (P = 0.09)									
Test for subgroup differences: Chi² = 2.97, df = 1 (P = 0.08), I² = 66.3%									

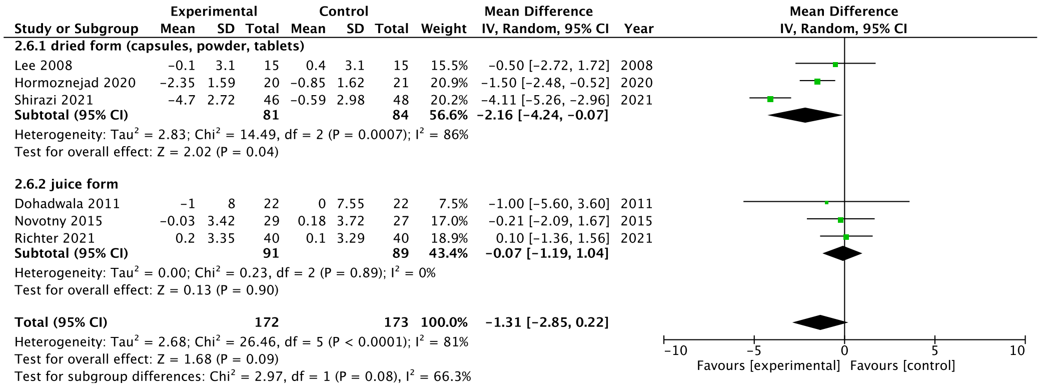

Supplementary Figure 11. Subgroup analysis of the effect of dosage form on fasting insulin.

Study or Subgroup	Experimental Mean	Experimental SD	Experimental Total	Control Mean	Control SD	Control Total	Weight	Mean Difference IV, Random, 95% CI	Year
**3.1.1 intervention duration ≥ 8 weeks**									
Lee 2008	−0.4	0.39	15	0.3	0.39	15	11.5%	−0.70 [−0.98, −0.42]	2008
Basu 2011	−0.14	0.85	15	0.13	0.81	16	4.6%	−0.27 [−0.86, 0.32]	2011
Flammer 2013	0.1	0.16	32	0.14	0.18	37	19.4%	−0.04 [−0.12, 0.04]	2013
Novotny 2015	0.08	0.61	29	0	0.73	27	9.1%	0.08 [−0.27, 0.43]	2015
Javid 2017	−0.01	1.07	9	−0.2	0.83	12	2.5%	0.19 [−0.65, 1.03]	2017
Hormoznejad 2020	−0.04	1.17	20	−0.22	0.74	21	4.4%	0.18 [−0.42, 0.78]	2020
Richter 2021	−0.03	0.85	40	−0.03	0.85	40	8.5%	0.00 [−0.37, 0.37]	2021
Shirazi 2021	−0.54	1.65	46	−0.13	1.32	48	4.3%	−0.41 [−1.02, 0.20]	2021
Flanagan 2022	−0.2	1.15	29	−0.1	1.05	31	4.9%	−0.10 [−0.66, 0.46]	2022
Rahn 2023	−0.01	0.24	18	−0.04	0.39	18	14.2%	0.03 [−0.18, 0.24]	2023
**Subtotal (95% CI)**			**253**			**265**	**83.4%**	**−0.12 [−0.30, 0.06]**	
Heterogeneity: Tau² = 0.04; Chi² = 24.49, df = 9 (P = 0.004); I² = 63%									
Test for overall effect: Z = 1.34 (P = 0.18)									
**3.1.2 intervention duration < 8 weeks**									
Duthie 2006	−0.01	0.65	11	−0.03	0.75	9	4.2%	0.02 [−0.60, 0.64]	2006
Dohadwala 2011	−0.05	1.02	22	0	1.07	22	4.2%	−0.05 [−0.67, 0.57]	2011
Paquette 2017	−0.1	0.81	20	0.08	0.97	21	5.1%	−0.18 [−0.73, 0.37]	2017
Heiss 2022	−0.13	0.51	22	0.01	1.69	22	3.1%	−0.14 [−0.88, 0.60]	2022
**Subtotal (95% CI)**			**75**			**74**	**16.6%**	**−0.09 [−0.40, 0.22]**	
Heterogeneity: Tau² = 0.00; Chi² = 0.26, df = 3 (P = 0.97); I² = 0%									
Test for overall effect: Z = 0.57 (P = 0.57)									
**Total (95% CI)**			**328**			**339**	**100.0%**	**−0.11 [−0.26, 0.03]**	
Heterogeneity: Tau² = 0.03; Chi² = 24.76, df = 13 (P = 0.02); I² = 48%									
Test for overall effect: Z = 1.57 (P = 0.12)									
Test for subgroup differences: Chi² = 0.03, df = 1 (P = 0.87), I² = 0%									

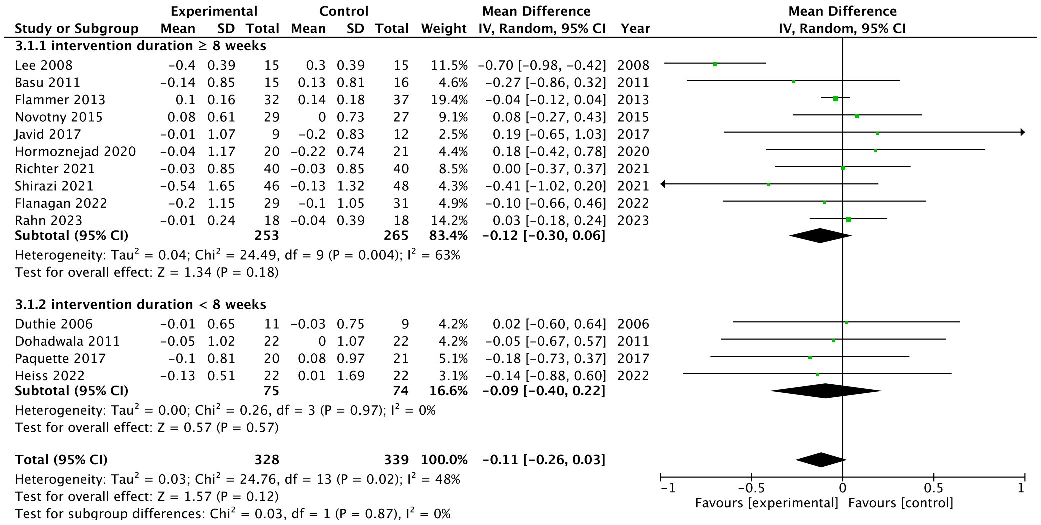

Supplementary Figure 12. Subgroup analysis of the effect of intervention duration on TC.

Study or Subgroup	Experimental Mean	Experimental SD	Experimental Total	Control Mean	Control SD	Control Total	Weight	Mean Difference IV, Fixed, 95% CI
**3.2.1 intervention duration ≥ 8 weeks**								
Basu 2011	−0.04	0.21	15	−0.01	0.25	16	4.2%	−0.03 [−0.19, 0.13]
Flammer 2013	0	0.21	32	0.12	0.4	37	5.0%	−0.12 [−0.27, 0.03]
Flanagan 2022	0	0.36	29	0	0.4	31	3.0%	0.00 [−0.19, 0.19]
Hormoznejad 2020	−0.01	0.17	20	0.09	0.17	21	10.1%	−0.10 [−0.20, 0.00]
Javid 2017	0.03	0.18	9	0.17	0.26	12	3.1%	−0.14 [−0.33, 0.05]
Lee 2008	0	0.39	15	0.1	0.04	15	2.8%	−0.10 [−0.30, 0.10]
Novotny 2015	0.04	0.23	29	0.03	0.32	27	5.1%	0.01 [−0.14, 0.16]
Rahn 2023	−0.12	0.28	18	−0.11	0.33	18	2.7%	−0.01 [−0.21, 0.19]
Richter 2021	0	0.28	40	0	0.28	40	7.3%	0.00 [−0.12, 0.12]
Shirazi 2021	0.12	0.12	46	0.09	0.13	48	42.9%	0.03 [−0.02, 0.08]
**Subtotal (95% CI)**			**253**			**265**	**86.1%**	**−0.01 [−0.05, 0.02]**
Heterogeneity: Chi² = 10.15, df = 9 (P = 0.34); I² = 11%								
Test for overall effect: Z = 0.73 (P = 0.47)								
**3.2.2 intervention duration < 8 weeks**								
Dohadwala 2011	−0.03	0.25	22	0.03	0.23	22	5.4%	−0.06 [−0.20, 0.08]
Duthie 2006	0	0.32	11	−0.02	0.22	9	1.9%	0.02 [−0.22, 0.26]
Heiss 2022	−0.13	0.18	22	0.07	0.53	22	2.0%	−0.20 [−0.43, 0.03]
Paquette 2017	0.01	0.25	20	0.04	0.26	21	4.5%	−0.03 [−0.19, 0.13]
**Subtotal (95% CI)**			**75**			**74**	**13.9%**	**−0.06 [−0.15, 0.03]**
Heterogeneity: Chi² = 1.95, df = 3 (P = 0.58); I² = 0%								
Test for overall effect: Z = 1.31 (P = 0.19)								
**Total (95% CI)**			**328**			**339**	**100.0%**	**−0.02 [−0.05, 0.01]**
Heterogeneity: Chi² = 13.00, df = 13 (P = 0.45); I² = 0%								
Test for overall effect: Z = 1.16 (P = 0.25)								
Test for subgroup differences: Chi² = 0.89, df = 1 (P = 0.35), I² = 0%								

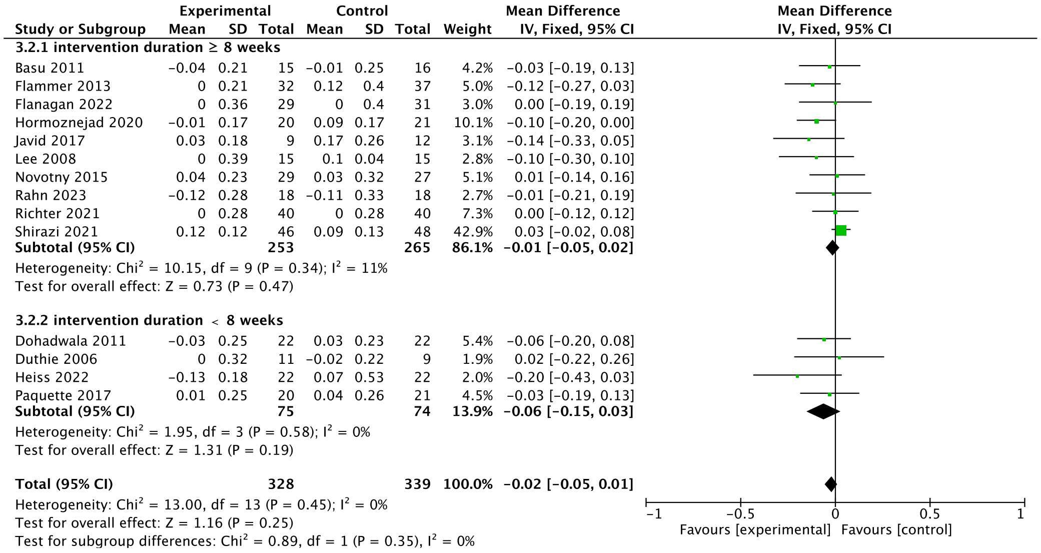

Supplementary Figure 13. Subgroup analysis of the effect of intervention duration on HDL-C.

Study or Subgroup	Experimental Mean	Experimental SD	Experimental Total	Control Mean	Control SD	Control Total	Weight	Mean Difference IV, Random, 95% CI
**3.3.1 intervention duration ≥ 8 weeks**								
Basu 2011	−0.13	0.67	15	0.1	0.68	16	6.6%	−0.23 [−0.71, 0.25]
Flanagan 2022	−0.3	1	29	−0.1	1.08	31	5.7%	−0.20 [−0.73, 0.33]
Hormoznejad 2020	0.09	1.13	20	−0.23	0.84	21	4.5%	0.32 [−0.29, 0.93]
Javid 2017	0.08	1.39	9	−0.25	0.72	12	1.9%	0.33 [−0.67, 1.33]
Lee 2008	−0.4	0.39	15	0.2	0.39	15	12.4%	−0.60 [−0.88, −0.32]
Novotny 2015	0.1	0.51	29	0.03	0.5	27	13.0%	0.07 [−0.19, 0.33]
Rahn 2023	−0.03	0.5	18	−0.08	0.75	18	7.9%	0.05 [−0.37, 0.47]
Richter 2021	0	0.71	40	−0.08	0.71	40	11.1%	0.08 [−0.23, 0.39]
Shirazi 2021	−0.26	0.53	46	−0.21	0.51	48	15.4%	−0.05 [−0.26, 0.16]
**Subtotal (95% CI)**			**221**			**228**	**78.6%**	**−0.08 [−0.27, 0.12]**
Heterogeneity: Tau² = 0.04; Chi² = 18.86, df = 8 (P = 0.02); I² = 58%								
Test for overall effect: Z = 0.78 (P = 0.43)								
**3.3.2 intervention duration < 8 weeks**								
Dohadwala 2011	0	0.85	22	−0.03	0.89	22	5.9%	0.03 [−0.48, 0.54]
Duthie 2006	−0.38	0.65	11	−0.2	0.69	9	4.8%	−0.18 [−0.77, 0.41]
Heiss 2022	−0.05	0.56	22	0.04	1.53	22	3.8%	−0.09 [−0.77, 0.59]
Paquette 2017	−0.01	0.76	20	0.17	0.74	21	6.9%	−0.18 [−0.64, 0.28]
**Subtotal (95% CI)**			**75**			**74**	**21.4%**	**−0.11 [−0.38, 0.16]**
Heterogeneity: Tau² = 0.00; Chi² = 0.43, df = 3 (P = 0.93); I² = 0%								
Test for overall effect: Z = 0.77 (P = 0.44)								
**Total (95% CI)**			**296**			**302**	**100.0%**	**−0.09 [−0.23, 0.06]**
Heterogeneity: Tau² = 0.02; Chi² = 19.29, df = 12 (P = 0.08); I² = 38%								
Test for overall effect: Z = 1.19 (P = 0.23)								
Test for subgroup differences: Chi² = 0.03, df = 1 (P = 0.86), I² = 0%								

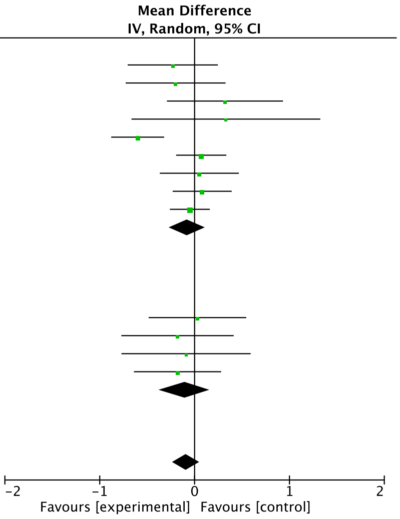

Supplementary Figure 14. Subgroup analysis of the effect of intervention duration on LDL-C.

Study or Subgroup	Experimental Mean	Experimental SD	Experimental Total	Control Mean	Control SD	Control Total	Weight	Mean Difference IV, Random, 95% CI	Year
**3.4.1 intervention duration ≥ 8 weeks**									
Lee 2008	−0.1	0.77	15	0	0.39	15	3.3%	−0.10 [−0.54, 0.34]	2008
Basu 2011	0.07	0.16	15	−0.04	0.11	16	17.5%	0.11 [0.01, 0.21]	2011
Flammer 2013	0.07	0.31	32	−0.1	0.33	37	13.3%	0.17 [0.02, 0.32]	2013
Novotny 2015	−0.13	0.47	29	−0.03	0.5	27	7.5%	−0.10 [−0.35, 0.15]	2015
Javid 2017	−0.24	1.01	9	−0.25	0.39	12	1.4%	0.01 [−0.69, 0.71]	2017
Hormoznejad 2020	−0.17	0.39	20	−0.55	1.37	21	1.8%	0.38 [−0.23, 0.99]	2020
Hsia 2020	−0.16	0.42	18	0.1	0.37	17	7.3%	−0.26 [−0.52, 0.00]	2020
Richter 2021	−0.01	0.43	40	0.06	0.47	40	10.3%	−0.07 [−0.27, 0.13]	2021
Shirazi 2021	−0.09	0.74	46	0.08	0.71	48	6.2%	−0.17 [−0.46, 0.12]	2021
Flanagan 2022	0.1	0.4	29	−0.1	0.56	31	7.9%	0.20 [−0.05, 0.45]	2022
Rahn 2023	0.09	0.56	18	0.24	0.42	18	5.3%	−0.15 [−0.47, 0.17]	2023
**Subtotal (95% CI)**			**271**			**282**	**81.8%**	**0.01 [−0.10, 0.11]**	
Heterogeneity: Tau² = 0.01; Chi² = 18.68, df = 10 (P = 0.04); I² = 46%									
Test for overall effect: Z = 0.12 (P = 0.90)									
**3.4.2 intervention duration < 8 weeks**									
Duthie 2006	0.24	0.37	11	−0.01	0.35	9	5.5%	0.25 [−0.07, 0.57]	2006
Dohadwala 2011	−0.02	0.61	22	0.01	0.69	22	4.1%	−0.03 [−0.41, 0.35]	2011
Paquette 2017	−0.21	1.01	20	−0.17	1.06	21	1.7%	−0.04 [−0.67, 0.59]	2017
Heiss 2022	0.13	0.46	22	−0.07	0.46	22	6.9%	0.20 [−0.07, 0.47]	2022
**Subtotal (95% CI)**			**75**			**74**	**18.2%**	**0.15 [−0.03, 0.32]**	
Heterogeneity: Tau² = 0.00; Chi² = 1.70, df = 3 (P = 0.64); I² = 0%									
Test for overall effect: Z = 1.68 (P = 0.09)									
**Total (95% CI)**			**346**			**356**	**100.0%**	**0.04 [−0.05, 0.12]**	
Heterogeneity: Tau² = 0.01; Chi² = 21.50, df = 14 (P = 0.09); I² = 35%									
Test for overall effect: Z = 0.80 (P = 0.42)									
Test for subgroup differences: Chi² = 1.92, df = 1 (P = 0.17), I² = 48.0%									

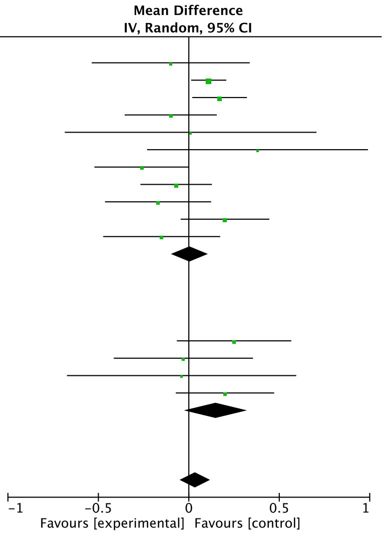

Supplementary Figure 15. Subgroup analysis of the effect of intervention duration on TG.

Study or Subgroup	Experimental Mean	Experimental SD	Experimental Total	Control Mean	Control SD	Control Total	Weight	Mean Difference IV, Random, 95% CI	Year
**3.5.1 intervention duration ≥ 8 weeks**									
Lee 2008	−0.6	2.32	15	−0.4	1.55	15	0.8%	−0.20 [−1.61, 1.21]	2008
Basu 2011	0.32	0.45	15	0.06	0.47	16	8.6%	0.26 [−0.06, 0.58]	2011
Shidfar 2011	−1.09	0.73	29	0.09	1.22	29	4.8%	−1.18 [−1.70, −0.66]	2011
Novotny 2015	−0.1	0.38	29	0.06	0.36	27	12.8%	−0.16 [−0.35, 0.03]	2015
Javid 2017	−0.17	3.85	9	−1.74	3.59	12	0.2%	1.57 [−1.66, 4.80]	2017
Hormoznejad 2020	−0.3	0.78	20	0.04	1.07	21	4.1%	−0.34 [−0.91, 0.23]	2020
Hsia 2020	0.04	0.38	18	−0.02	0.37	17	10.9%	0.06 [−0.19, 0.31]	2020
Richter 2021	0	0.35	40	0.11	0.35	40	14.3%	−0.11 [−0.26, 0.04]	2021
Shirazi 2021	−0.36	0.49	46	−0.3	0.47	48	12.8%	−0.06 [−0.25, 0.13]	2021
Flanagan 2022	−0.1	0.4	29	−0.1	0.5	31	11.6%	0.00 [−0.23, 0.23]	2022
**Subtotal (95% CI)**			**250**			**256**	**80.8%**	**−0.11 [−0.28, 0.05]**	
Heterogeneity: Tau^2 = 0.04; Chi^2 = 25.94, df = 9 (P = 0.002); I^2 = 65%									
Test for overall effect: Z = 1.33 (P = 0.18)									
**3.5.2 intervention duration < 8 weeks**									
Dohadwala 2011	−0.17	2.69	22	0.22	2.69	22	0.7%	−0.39 [−1.98, 1.20]	2011
Paquette 2017	0.1	0.45	20	0.1	0.46	21	9.9%	0.00 [−0.28, 0.28]	2017
Heiss 2022	0.01	0.49	22	0.08	0.6	22	8.6%	−0.07 [−0.39, 0.25]	2022
**Subtotal (95% CI)**			**64**			**65**	**19.2%**	**−0.04 [−0.25, 0.17]**	
Heterogeneity: Tau^2 = 0.00; Chi^2 = 0.30, df = 2 (P = 0.86); I^2 = 0%									
Test for overall effect: Z = 0.34 (P = 0.74)									
**Total (95% CI)**			**314**			**321**	**100.0%**	**−0.09 [−0.23, 0.04]**	
Heterogeneity: Tau^2 = 0.03; Chi^2 = 26.41, df = 12 (P = 0.009); I^2 = 55%									
Test for overall effect: Z = 1.38 (P = 0.17)									
Test for subgroup differences: Chi^2 = 0.31, df = 1 (P = 0.58), I^2 = 0%									

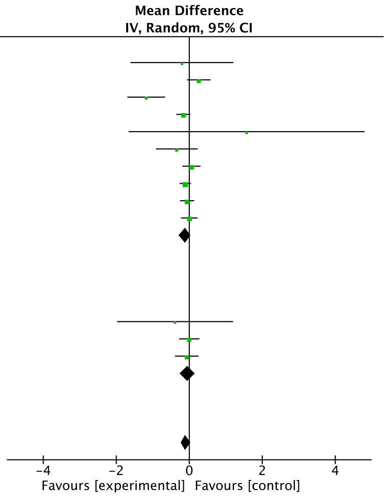

Supplementary Figure 16. Subgroup analysis of the effect of intervention duration on FBG.

Study or Subgroup	Experimental Mean	Experimental SD	Experimental Total	Control Mean	Control SD	Control Total	Weight	Mean Difference IV, Random, 95% CI	Year
**3.6.1 intervention duration ≥ 8 weeks**									
Lee 2008	−0.1	3.1	15	0.4	3.1	15	15.5%	−0.50 [−2.72, 1.72]	2008
Novotny 2015	−0.03	3.42	29	0.18	3.72	27	17.0%	−0.21 [−2.09, 1.67]	2015
Hormoznejad 2020	−2.35	1.59	20	−0.85	1.62	21	20.9%	−1.50 [−2.48, −0.52]	2020
Shirazi 2021	−4.7	2.72	46	−0.59	2.98	48	20.2%	−4.11 [−5.26, −2.96]	2021
Richter 2021	0.2	3.35	40	0.1	3.29	40	18.9%	0.10 [−1.36, 1.56]	2021
**Subtotal (95% CI)**			**150**			**151**	**92.5%**	**−1.33 [−2.97, 0.31]**	
Heterogeneity: Tau^2 = 2.87; Chi^2 = 26.37, df = 4 (P < 0.0001); I^2 = 85%									
Test for overall effect: Z = 1.59 (P = 0.11)									
**3.6.2 intervention duration < 8 weeks**									
Dohadwala 2011	−1	8	22	0	7.55	22	7.5%	−1.00 [−5.60, 3.60]	2011
**Subtotal (95% CI)**			**22**			**22**	**7.5%**	**−1.00 [−5.60, 3.60]**	
Heterogeneity: Not applicable									
Test for overall effect: Z = 0.43 (P = 0.67)									
**Total (95% CI)**			**172**			**173**	**100.0%**	**−1.31 [−2.85, 0.22]**	
Heterogeneity: Tau^2 = 2.68; Chi^2 = 26.46, df = 5 (P < 0.0001); I^2 = 81%									
Test for overall effect: Z = 1.68 (P = 0.09)									
Test for subgroup differences: Chi^2 = 0.02, df = 1 (P = 0.89), I^2 = 0%									

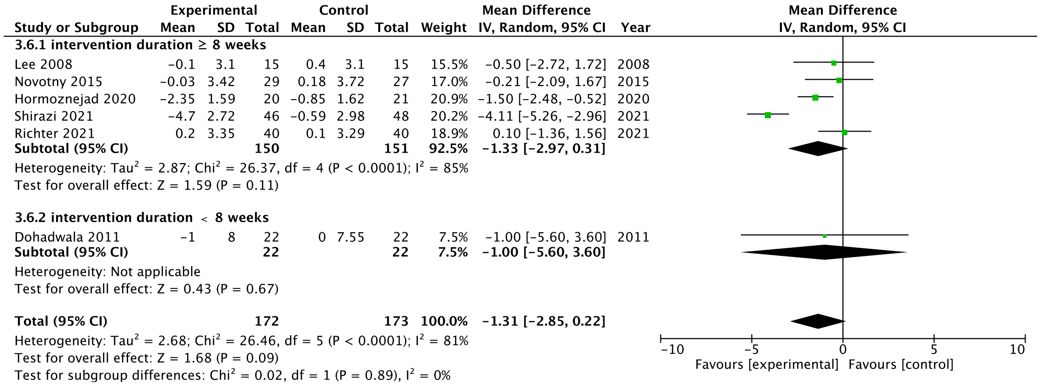

Supplementary Figure 17. Subgroup analysis of the effect of intervention duration on fasting insulin.

Study or Subgroup	Experimental Mean	Experimental SD	Experimental Total	Control Mean	Control SD	Control Total	Weight	Mean Difference IV, Random, 95% CI	Year
**4.1.1 healthy population**									
Duthie 2006	−0.01	0.65	11	−0.03	0.75	9	4.2%	0.02 [−0.60, 0.64]	2006
Novotny 2015	0.08	0.61	29	0	0.73	27	9.1%	0.08 [−0.27, 0.43]	2015
Flanagan 2022	−0.2	1.15	29	−0.1	1.05	31	4.9%	−0.10 [−0.66, 0.46]	2022
Heiss 2022	−0.13	0.51	22	0.01	1.69	22	3.1%	−0.14 [−0.88, 0.60]	2022
Rahn 2023	−0.01	0.24	18	−0.04	0.39	18	14.2%	0.03 [−0.18, 0.24]	2023
**Subtotal (95% CI)**			**109**			**107**	**35.5%**	**0.02 [−0.14, 0.18]**	
Heterogeneity: Tau^2 = 0.00; Chi^2 = 0.48, df = 4 (P = 0.98); I^2 = 0%									
Test for overall effect: Z = 0.25 (P = 0.80)									
**4.1.2 population with metabolic diseases**									
Lee 2008	−0.4	0.39	15	0.3	0.39	15	11.5%	−0.70 [−0.98, −0.42]	2008
Basu 2011	−0.14	0.85	15	0.13	0.81	16	4.6%	−0.27 [−0.86, 0.32]	2011
Javid 2017	−0.01	1.07	9	−0.2	0.83	12	2.5%	0.19 [−0.65, 1.03]	2017
Paquette 2017	−0.1	0.81	20	0.08	0.97	21	5.1%	−0.18 [−0.73, 0.37]	2017
Hormoznejad 2020	−0.04	1.17	20	−0.22	0.74	21	4.4%	0.18 [−0.42, 0.78]	2020
Richter 2021	−0.03	0.85	40	−0.03	0.85	40	8.5%	0.00 [−0.37, 0.37]	2021
Shirazi 2021	−0.54	1.65	46	−0.13	1.32	48	4.3%	−0.41 [−1.02, 0.20]	2021
**Subtotal (95% CI)**			**165**			**173**	**41.0%**	**−0.22 [−0.52, 0.07]**	
Heterogeneity: Tau^2 = 0.09; Chi^2 = 14.37, df = 6 (P = 0.03); I^2 = 58%									
Test for overall effect: Z = 1.48 (P = 0.14)									
**4.1.3 population with coronary artery disease or CVD risk**									
Dohadwala 2011	−0.05	1.02	22	0	1.07	22	4.2%	−0.05 [−0.67, 0.57]	2011
Flammer 2013	0.1	0.16	32	0.14	0.18	37	19.4%	−0.04 [−0.12, 0.04]	2013
**Subtotal (95% CI)**			**54**			**59**	**23.6%**	**−0.04 [−0.12, 0.04]**	
Heterogeneity: Tau^2 = 0.00; Chi^2 = 0.00, df = 1 (P = 0.97); I^2 = 0%									
Test for overall effect: Z = 0.99 (P = 0.32)									
**Total (95% CI)**			**328**			**339**	**100.0%**	**−0.11 [−0.26, 0.03]**	
Heterogeneity: Tau^2 = 0.03; Chi^2 = 24.76, df = 13 (P = 0.02); I^2 = 48%									
Test for overall effect: Z = 1.57 (P = 0.12)									
Test for subgroup differences: Chi^2 = 2.00, df = 2 (P = 0.37), I^2 = 0.2%									

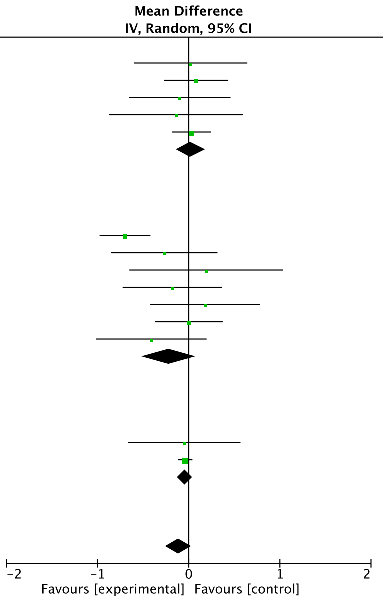

Supplementary Figure 18. Subgroup analysis of the effect of health conditions on TC.

Study or Subgroup	Experimental Mean	Experimental SD	Experimental Total	Control Mean	Control SD	Control Total	Weight	Mean Difference IV, Fixed, 95% CI	Year
**4.2.1 healthy population**									
Duthie 2006	0	0.32	11	−0.02	0.22	9	1.9%	0.02 [−0.22, 0.26]	2006
Novotny 2015	0.04	0.23	29	0.03	0.32	27	5.1%	0.01 [−0.14, 0.16]	2015
Flanagan 2022	0	0.36	29	0	0.4	31	3.0%	0.00 [−0.19, 0.19]	2022
Heiss 2022	−0.13	0.18	22	0.07	0.53	22	2.0%	−0.20 [−0.43, 0.03]	2022
Rahn 2023	−0.12	0.28	18	−0.11	0.33	18	2.7%	−0.01 [−0.21, 0.19]	2023
**Subtotal (95% CI)**			**109**			**107**	**14.7%**	**−0.02 [−0.11, 0.06]**	
Heterogeneity: Chi² = 2.59, df = 4 (P = 0.63); I² = 0%									
Test for overall effect: Z = 0.52 (P = 0.60)									
**4.2.2 population with metabolic diseases**									
Lee 2008	0	0.39	15	0.1	0.04	15	2.8%	−0.10 [−0.30, 0.10]	2008
Basu 2011	−0.04	0.21	15	−0.01	0.25	16	4.2%	−0.03 [−0.19, 0.13]	2011
Paquette 2017	0.01	0.25	20	0.04	0.26	21	4.5%	−0.03 [−0.19, 0.13]	2017
Javid 2017	0.03	0.18	9	0.17	0.26	12	3.1%	−0.14 [−0.33, 0.05]	2017
Hormoznejad 2020	−0.01	0.17	20	0.09	0.17	21	10.1%	−0.10 [−0.20, 0.00]	2020
Richter 2021	0	0.28	40	0	0.28	40	7.3%	0.00 [−0.12, 0.12]	2021
Shirazi 2021	0.12	0.12	46	0.09	0.13	48	42.9%	0.03 [−0.02, 0.08]	2021
**Subtotal (95% CI)**			**165**			**173**	**74.8%**	**−0.01 [−0.05, 0.03]**	
Heterogeneity: Chi² = 8.04, df = 6 (P = 0.23); I² = 25%									
Test for overall effect: Z = 0.48 (P = 0.63)									
**4.2.3 population with coronary artery disease or CVD risk**									
Dohadwala 2011	−0.03	0.25	22	0.03	0.23	22	5.4%	−0.06 [−0.20, 0.08]	2011
Flammer 2013	0	0.21	32	0.12	0.4	37	5.0%	−0.12 [−0.27, 0.03]	2013
**Subtotal (95% CI)**			**54**			**59**	**10.4%**	**−0.09 [−0.19, 0.01]**	
Heterogeneity: Chi² = 0.33, df = 1 (P = 0.57); I² = 0%									
Test for overall effect: Z = 1.70 (P = 0.09)									
**Total (95% CI)**			**328**			**339**	**100.0%**	**−0.02 [−0.05, 0.01]**	
Heterogeneity: Chi² = 13.00, df = 13 (P = 0.45); I² = 0%									
Test for overall effect: Z = 1.16 (P = 0.25)									
Test for subgroup differences: Chi² = 2.03, df = 2 (P = 0.36), I² = 1.7%									

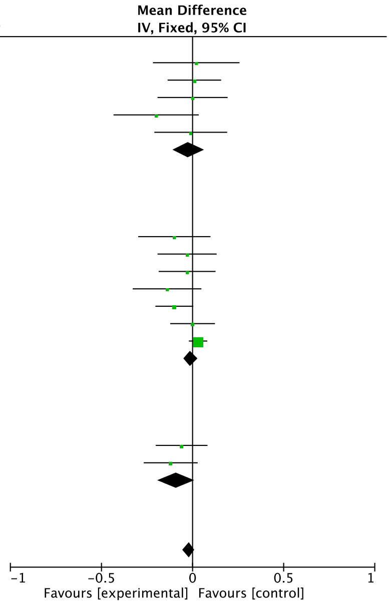

Supplementary Figure 19. Subgroup analysis of the effect of health conditions on HDL-C.

Study or Subgroup	Experimental Mean	Experimental SD	Experimental Total	Control Mean	Control SD	Control Total	Weight	Mean Difference IV, Random, 95% CI	Year
**4.3.1 healthy population**									
Duthie 2006	−0.38	0.65	11	−0.2	0.69	9	4.8%	−0.18 [−0.77, 0.41]	2006
Novotny 2015	0.1	0.51	29	0.03	0.5	27	13.0%	0.07 [−0.19, 0.33]	2015
Flanagan 2022	−0.3	1	29	−0.1	1.08	31	5.7%	−0.20 [−0.73, 0.33]	2022
Heiss 2022	−0.05	0.56	22	0.04	1.53	22	3.8%	−0.09 [−0.77, 0.59]	2022
Rahn 2023	−0.03	0.5	18	−0.08	0.75	18	7.9%	0.05 [−0.37, 0.47]	2023
**Subtotal (95% CI)**			**109**			**107**	**35.2%**	**−0.00 [−0.19, 0.18]**	
Heterogeneity: Tau² = 0.00; Chi² = 1.30, df = 4 (P = 0.86); I² = 0%									
Test for overall effect: Z = 0.05 (P = 0.96)									
**4.3.2 population with metabolic diseases**									
Lee 2008	−0.4	0.39	15	0.2	0.39	15	12.4%	−0.60 [−0.88, −0.32]	2008
Basu 2011	−0.13	0.67	15	0.1	0.68	16	6.6%	−0.23 [−0.71, 0.25]	2011
Javid 2017	0.08	1.39	9	−0.25	0.72	12	1.9%	0.33 [−0.67, 1.33]	2017
Paquette 2017	−0.01	0.76	20	0.17	0.74	21	6.9%	−0.18 [−0.64, 0.28]	2017
Hormoznejad 2020	0.09	1.13	20	−0.23	0.84	21	4.5%	0.32 [−0.29, 0.93]	2020
Richter 2021	0	0.71	40	−0.08	0.71	40	11.1%	0.08 [−0.23, 0.39]	2021
Shirazi 2021	−0.26	0.53	46	−0.21	0.51	48	15.4%	−0.05 [−0.26, 0.16]	2021
**Subtotal (95% CI)**			**165**			**173**	**58.9%**	**−0.12 [−0.36, 0.13]**	
Heterogeneity: Tau² = 0.06; Chi² = 16.24, df = 6 (P = 0.01); I² = 63%									
Test for overall effect: Z = 0.95 (P = 0.34)									
**4.3.3 population with coronary artery disease or CVD risk**									
Dohadwala 2011	0	0.85	22	−0.03	0.89	22	5.9%	0.03 [−0.48, 0.54]	2011
**Subtotal (95% CI)**			**22**			**22**	**5.9%**	**0.03 [−0.48, 0.54]**	
Heterogeneity: Not applicable									
Test for overall effect: Z = 0.11 (P = 0.91)									
**Total (95% CI)**			**296**			**302**	**100.0%**	**−0.09 [−0.23, 0.06]**	
Heterogeneity: Tau² = 0.02; Chi² = 19.29, df = 12 (P = 0.08); I² = 38%									
Test for overall effect: Z = 1.19 (P = 0.23)									
Test for subgroup differences: Chi² = 0.60, df = 2 (P = 0.74), I² = 0%									

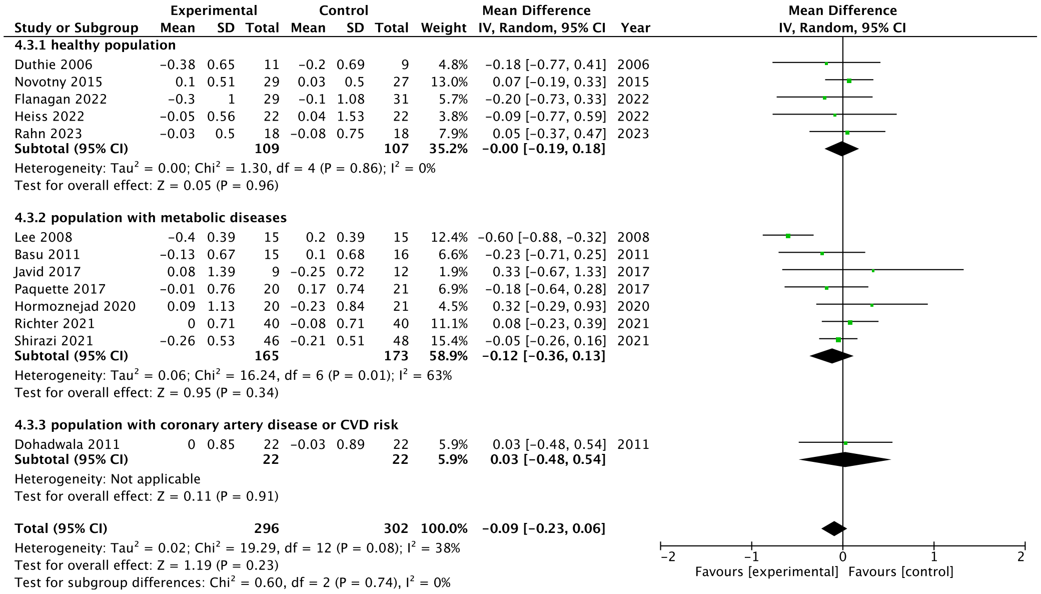

Supplementary Figure 20. Subgroup analysis of the effect of health conditions on LDL-C.

Study or Subgroup	Experimental Mean	Experimental SD	Experimental Total	Control Mean	Control SD	Control Total	Weight	Mean Difference IV, Fixed, 95% CI	Year
**4.4.1 healthy population**									
Duthie 2006	0.24	0.37	11	−0.01	0.35	9	3.5%	0.25 [−0.07, 0.57]	2006
Novotny 2015	−0.13	0.47	29	−0.03	0.5	27	5.4%	−0.10 [−0.35, 0.15]	2015
Flanagan 2022	0.1	0.4	29	−0.1	0.56	31	5.8%	0.20 [−0.05, 0.45]	2022
Heiss 2022	0.13	0.46	22	−0.07	0.46	22	4.7%	0.20 [−0.07, 0.47]	2022
Rahn 2023	0.09	0.56	18	0.24	0.42	18	3.3%	−0.15 [−0.47, 0.17]	2023
**Subtotal (95% CI)**			**109**			**107**	**22.8%**	**0.09 [−0.04, 0.21]**	
Heterogeneity: Chi² = 6.63, df = 4 (P = 0.16); I² = 40%									
Test for overall effect: Z = 1.35 (P = 0.18)									
**4.4.2 population with metabolic diseases**									
Lee 2008	−0.1	0.77	15	0	0.39	15	1.8%	−0.10 [−0.54, 0.34]	2008
Basu 2011	0.07	0.16	15	−0.04	0.11	16	37.0%	0.11 [0.01, 0.21]	2011
Javid 2017	−0.24	1.01	9	−0.25	0.39	12	0.7%	0.01 [−0.69, 0.71]	2017
Paquette 2017	−0.21	1.01	20	−0.17	1.06	21	0.9%	−0.04 [−0.67, 0.59]	2017
Hormoznejad 2020	−0.17	0.39	20	−0.55	1.37	21	0.9%	0.38 [−0.23, 0.99]	2020
Hsia 2020	−0.16	0.42	18	0.1	0.37	17	5.1%	−0.26 [−0.52, 0.00]	2020
Richter 2021	−0.01	0.43	40	0.06	0.47	40	9.0%	−0.07 [−0.27, 0.13]	2021
Shirazi 2021	−0.09	0.74	46	0.08	0.71	48	4.1%	−0.17 [−0.46, 0.12]	2021
**Subtotal (95% CI)**			**183**			**190**	**59.5%**	**0.03 [−0.05, 0.10]**	
Heterogeneity: Chi² = 11.72, df = 7 (P = 0.11); I² = 40%									
Test for overall effect: Z = 0.67 (P = 0.50)									
**4.4.3 population with coronary artery disease or CVD risk**									
Dohadwala 2011	−0.02	0.61	22	0.01	0.69	22	2.4%	−0.03 [−0.41, 0.35]	2011
Flammer 2013	0.07	0.31	32	−0.1	0.33	37	15.3%	0.17 [0.02, 0.32]	2013
**Subtotal (95% CI)**			**54**			**59**	**17.7%**	**0.14 [0.00, 0.28]**	
Heterogeneity: Chi² = 0.90, df = 1 (P = 0.34); I² = 0%									
Test for overall effect: Z = 2.00 (P = 0.05)									
**Total (95% CI)**			**346**			**356**	**100.0%**	**0.06 [0.00, 0.12]**	
Heterogeneity: Chi² = 21.50, df = 14 (P = 0.09); I² = 35%									
Test for overall effect: Z = 2.00 (P = 0.05)									
Test for subgroup differences: Chi² = 2.25, df = 2 (P = 0.33), I² = 10.9%									

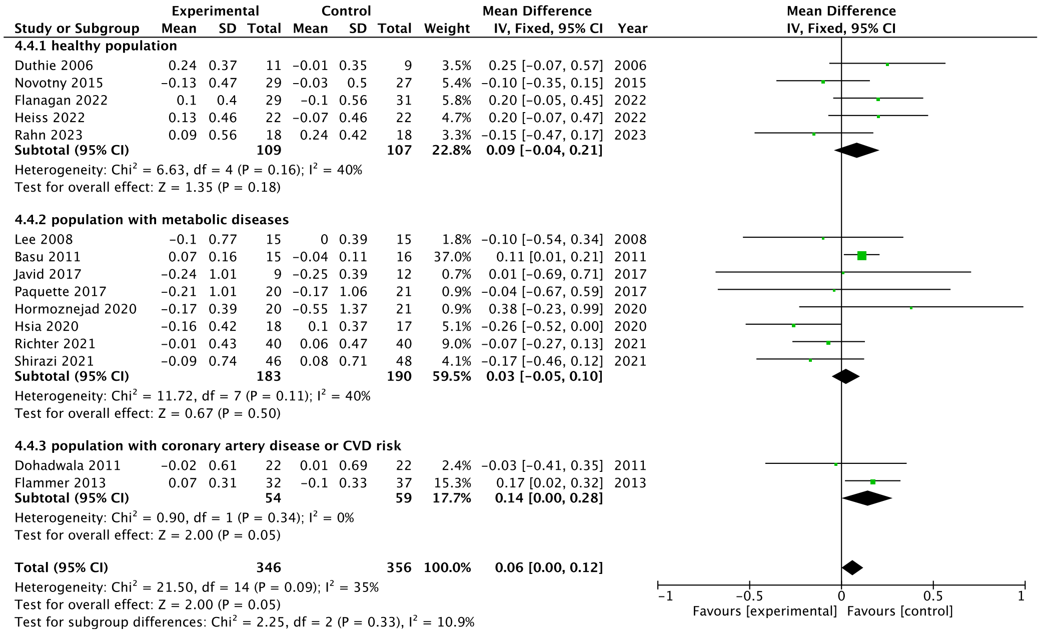

Supplementary Figure 21. Subgroup analysis of the effect of health conditions on TG.

Study or Subgroup	Experimental Mean	Experimental SD	Experimental Total	Control Mean	Control SD	Control Total	Weight	Mean Difference IV, Random, 95% CI	Year
**4.5.1 healthy population**									
Novotny 2015	−0.1	0.38	29	0.06	0.36	27	12.8%	−0.16 [−0.35, 0.03]	2015
Flanagan 2022	−0.1	0.4	29	−0.1	0.5	31	11.6%	0.00 [−0.23, 0.23]	2022
Heiss 2022	0.01	0.49	22	0.08	0.6	22	8.6%	−0.07 [−0.39, 0.25]	2022
**Subtotal (95% CI)**			**80**			**80**	**33.0%**	**−0.09 [−0.22, 0.05]**	
Heterogeneity: Tau² = 0.00; Chi² = 1.11, df = 2 (P = 0.57); I² = 0%									
Test for overall effect: Z = 1.30 (P = 0.19)									
**4.5.2 population with metabolic diseases**									
Lee 2008	−0.6	2.32	15	−0.4	1.55	15	0.8%	−0.20 [−1.61, 1.21]	2008
Shidfar 2011	−1.09	0.73	29	0.09	1.22	29	4.8%	−1.18 [−1.70, −0.66]	2011
Basu 2011	0.32	0.45	15	0.06	0.47	16	8.6%	0.26 [−0.06, 0.58]	2011
Javid 2017	−0.17	3.85	9	−1.74	3.59	12	0.2%	1.57 [−1.66, 4.80]	2017
Paquette 2017	0.1	0.45	20	0.1	0.46	21	9.9%	0.00 [−0.28, 0.28]	2017
Hormoznejad 2020	−0.3	0.78	20	0.04	1.07	21	4.1%	−0.34 [−0.91, 0.23]	2020
Hsia 2020	0.04	0.38	18	−0.02	0.37	17	10.9%	0.06 [−0.19, 0.31]	2020
Shirazi 2021	−0.36	0.49	46	−0.3	0.47	48	12.8%	−0.06 [−0.25, 0.13]	2021
Richter 2021	0	0.35	40	0.11	0.35	40	14.3%	−0.11 [−0.26, 0.04]	2021
**Subtotal (95% CI)**			**212**			**219**	**66.4%**	**−0.11 [−0.32, 0.09]**	
Heterogeneity: Tau² = 0.05; Chi² = 25.11, df = 8 (P = 0.001); I² = 68%									
Test for overall effect: Z = 1.11 (P = 0.27)									
**4.5.3 population with coronary artery disease or CVD risk**									
Dohadwala 2011	−0.17	2.69	22	0.22	2.69	22	0.7%	−0.39 [−1.98, 1.20]	2011
**Subtotal (95% CI)**			**22**			**22**	**0.7%**	**−0.39 [−1.98, 1.20]**	
Heterogeneity: Not applicable									
Test for overall effect: Z = 0.48 (P = 0.63)									
**Total (95% CI)**			**314**			**321**	**100.0%**	**−0.09 [−0.23, 0.04]**	
Heterogeneity: Tau² = 0.03; Chi² = 26.41, df = 12 (P = 0.009); I² = 55%									
Test for overall effect: Z = 1.38 (P = 0.17)									
Test for subgroup differences: Chi² = 0.17, df = 2 (P = 0.92), I² = 0%									

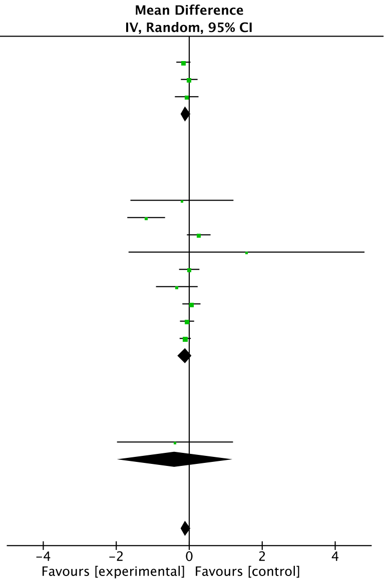

Supplementary Figure 22. Subgroup analysis of the effect of health conditions on FBG.

Study or Subgroup	Experimental Mean	Experimental SD	Experimental Total	Control Mean	Control SD	Control Total	Weight	Mean Difference IV, Random, 95% CI	Year
**4.6.1 healthy population**									
Novotny 2015	−0.03	3.42	29	0.18	3.72	27	17.0%	−0.21 [−2.09, 1.67]	2015
**Subtotal (95% CI)**			**29**			**27**	**17.0%**	**−0.21 [−2.09, 1.67]**	
Heterogeneity: Not applicable									
Test for overall effect: Z = 0.22 (P = 0.83)									
**4.6.2 population with metabolic diseases**									
Lee 2008	−0.1	3.1	15	0.4	3.1	15	15.5%	−0.50 [−2.72, 1.72]	2008
Hormoznejad 2020	−2.35	1.59	20	−0.85	1.62	21	20.9%	−1.50 [−2.48, −0.52]	2020
Richter 2021	0.2	3.35	40	0.1	3.29	40	18.9%	0.10 [−1.36, 1.56]	2021
Shirazi 2021	−4.7	2.72	46	−0.59	2.98	48	20.2%	−4.11 [−5.26, −2.96]	2021
**Subtotal (95% CI)**			**121**			**124**	**75.5%**	**−1.58 [−3.47, 0.31]**	
Heterogeneity: Tau² = 3.16; Chi² = 23.55, df = 3 (P < 0.0001); I² = 87%									
Test for overall effect: Z = 1.64 (P = 0.10)									
**4.6.3 population with coronary artery disease or CVD risk**									
Dohadwala 2011	−1	8	22	0	7.55	22	7.5%	−1.00 [−5.60, 3.60]	2011
**Subtotal (95% CI)**			**22**			**22**	**7.5%**	**−1.00 [−5.60, 3.60]**	
Heterogeneity: Not applicable									
Test for overall effect: Z = 0.43 (P = 0.67)									
**Total (95% CI)**			**172**			**173**	**100.0%**	**−1.31 [−2.85, 0.22]**	
Heterogeneity: Tau² = 2.68; Chi² = 26.46, df = 5 (P < 0.0001); I² = 81%									
Test for overall effect: Z = 1.68 (P = 0.09)									
Test for subgroup differences: Chi² = 1.02, df = 2 (P = 0.60), I² = 0%									

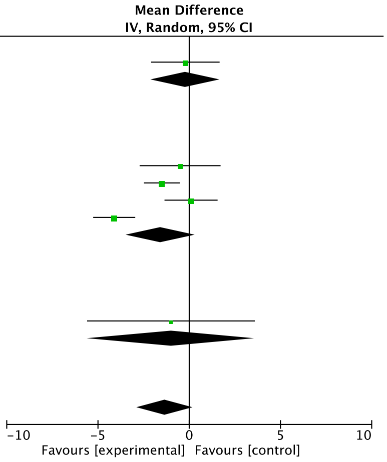

Supplementary Figure 23. Subgroup analysis of the effect of health conditions on fasting insulin.

Study or Subgroup	Experimental Mean	Experimental SD	Experimental Total	Control Mean	Control SD	Control Total	Weight	Mean Difference IV, Random, 95% CI	Year
**5.1.1 dosage of polyphenols ≥ 800 mg**									
Duthie 2006	−0.01	0.65	11	−0.03	0.75	9	4.2%	0.02 [−0.60, 0.64]	2006
Dohadwala 2011	−0.05	1.02	22	0	1.07	22	4.2%	−0.05 [−0.67, 0.57]	2011
Flammer 2013	0.1	0.16	32	0.14	0.18	37	19.4%	−0.04 [−0.12, 0.04]	2013
Rahn 2023	−0.01	0.24	18	−0.04	0.39	18	14.2%	0.03 [−0.18, 0.24]	2023
**Subtotal (95% CI)**			**83**			**86**	**41.9%**	**−0.03 [−0.10, 0.04]**	
Heterogeneity: $Tau^2 = 0.00$; $Chi^2 = 0.40$, df = 3 (P = 0.94); $I^2 = 0\%$									
Test for overall effect: Z = 0.81 (P = 0.42)									
**5.1.2 dosage of polyphenols < 800 mg**									
Lee 2008	−0.4	0.39	15	0.3	0.39	15	11.5%	−0.70 [−0.98, −0.42]	2008
Basu 2011	−0.14	0.85	15	0.13	0.81	16	4.6%	−0.27 [−0.86, 0.32]	2011
Novotny 2015	0.08	0.61	29	0	0.73	27	9.1%	0.08 [−0.27, 0.43]	2015
Javid 2017	−0.01	1.07	9	−0.2	0.83	12	2.5%	0.19 [−0.65, 1.03]	2017
Paquette 2017	−0.1	0.81	20	0.08	0.97	21	5.1%	−0.18 [−0.73, 0.37]	2017
Richter 2021	−0.03	0.85	40	−0.03	0.85	40	8.5%	0.00 [−0.37, 0.37]	2021
Heiss 2022	−0.13	0.51	22	0.01	1.69	22	3.1%	−0.14 [−0.88, 0.60]	2022
Flanagan 2022	−0.2	1.15	29	−0.1	1.05	31	4.9%	−0.10 [−0.66, 0.46]	2022
**Subtotal (95% CI)**			**179**			**184**	**49.4%**	**−0.18 [−0.44, 0.08]**	
Heterogeneity: $Tau^2 = 0.08$; $Chi^2 = 16.54$, df = 7 (P = 0.02); $I^2 = 58\%$									
Test for overall effect: Z = 1.33 (P = 0.18)									
**5.1.3 unavailable**									
Hormoznejad 2020	−0.04	1.17	20	−0.22	0.74	21	4.4%	0.18 [−0.42, 0.78]	2020
Shirazi 2021	−0.54	1.65	46	−0.13	1.32	48	4.3%	−0.41 [−1.02, 0.20]	2021
**Subtotal (95% CI)**			**66**			**69**	**8.7%**	**−0.11 [−0.69, 0.46]**	
Heterogeneity: $Tau^2 = 0.08$; $Chi^2 = 1.83$, df = 1 (P = 0.18); $I^2 = 45\%$									
Test for overall effect: Z = 0.39 (P = 0.70)									
**Total (95% CI)**			**328**			**339**	**100.0%**	**−0.11 [−0.26, 0.03]**	
Heterogeneity: $Tau^2 = 0.03$; $Chi^2 = 24.76$, df = 13 (P = 0.02); $I^2 = 48\%$									
Test for overall effect: Z = 1.57 (P = 0.12)									
Test for subgroup differences: $Chi^2 = 1.18$, df = 2 (P = 0.56), $I^2 = 0\%$									

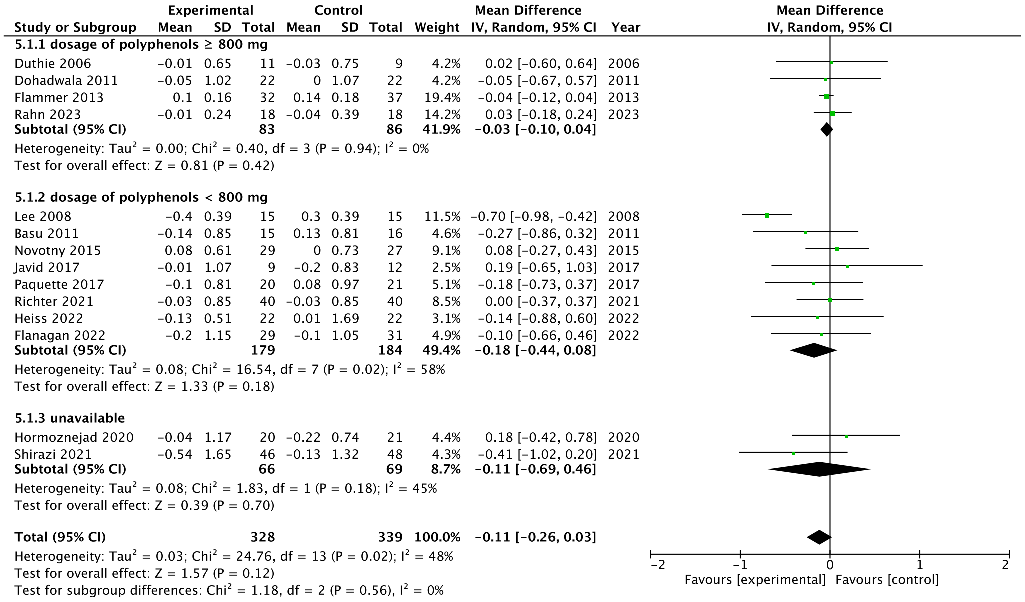

Supplementary Figure 24. Subgroup analysis of the effect of the dosage of total polyphenols on TC.

Study or Subgroup	Experimental Mean	Experimental SD	Experimental Total	Control Mean	Control SD	Control Total	Weight	Mean Difference IV, Random, 95% CI	Year
**5.2.1 dosage of polyphenols ≥ 800 mg**									
Duthie 2006	0	0.32	11	−0.02	0.22	9	1.9%	0.02 [−0.22, 0.26]	2006
Dohadwala 2011	−0.03	0.25	22	0.03	0.23	22	5.4%	−0.06 [−0.20, 0.08]	2011
Flammer 2013	0	0.21	32	0.12	0.4	37	5.0%	−0.12 [−0.27, 0.03]	2013
Rahn 2023	−0.12	0.28	18	−0.11	0.33	18	2.7%	−0.01 [−0.21, 0.19]	2023
**Subtotal (95% CI)**			**83**			**86**	**15.1%**	**−0.06 [−0.15, 0.02]**	
Heterogeneity: $Tau^2 = 0.00$; $Chi^2 = 1.31$, df = 3 (P = 0.73); $I^2 = 0\%$									
Test for overall effect: Z = 1.39 (P = 0.16)									
**5.2.2 dosage of polyphenols < 800 mg**									
Lee 2008	0	0.39	15	0.1	0.04	15	2.8%	−0.10 [−0.30, 0.10]	2008
Basu 2011	−0.04	0.21	15	−0.01	0.25	16	4.2%	−0.03 [−0.19, 0.13]	2011
Novotny 2015	0.04	0.23	29	0.03	0.32	27	5.1%	0.01 [−0.14, 0.16]	2015
Javid 2017	0.03	0.18	9	0.17	0.26	12	3.1%	−0.14 [−0.33, 0.05]	2017
Paquette 2017	0.01	0.25	20	0.04	0.26	21	4.5%	−0.03 [−0.19, 0.13]	2017
Richter 2021	0	0.28	40	0	0.28	40	7.3%	0.00 [−0.12, 0.12]	2021
Flanagan 2022	0	0.36	29	0	0.4	31	3.0%	0.00 [−0.19, 0.19]	2022
Heiss 2022	−0.13	0.18	22	0.07	0.53	22	2.0%	−0.20 [−0.43, 0.03]	2022
**Subtotal (95% CI)**			**179**			**184**	**31.9%**	**−0.04 [−0.10, 0.02]**	
Heterogeneity: $Tau^2 = 0.00$; $Chi^2 = 4.28$, df = 7 (P = 0.75); $I^2 = 0\%$									
Test for overall effect: Z = 1.39 (P = 0.17)									
**5.2.3 unavailable**									
Hormoznejad 2020	−0.01	0.17	20	0.09	0.17	21	10.1%	−0.10 [−0.20, 0.00]	2020
Shirazi 2021	0.12	0.12	46	0.09	0.13	48	42.9%	0.03 [−0.02, 0.08]	2021
**Subtotal (95% CI)**			**66**			**69**	**53.0%**	**−0.03 [−0.15, 0.10]**	
Heterogeneity: $Tau^2 = 0.01$; $Chi^2 = 4.85$, df = 1 (P = 0.03); $I^2 = 79\%$									
Test for overall effect: Z = 0.41 (P = 0.68)									
**Total (95% CI)**			**328**			**339**	**100.0%**	**−0.02 [−0.05, 0.01]**	
Heterogeneity: $Tau^2 = 0.00$; $Chi^2 = 13.00$, df = 13 (P = 0.45); $I^2 = 0\%$									
Test for overall effect: Z = 1.16 (P = 0.25)									
Test for subgroup differences: $Chi^2 = 0.22$, df = 2 (P = 0.89), $I^2 = 0\%$									

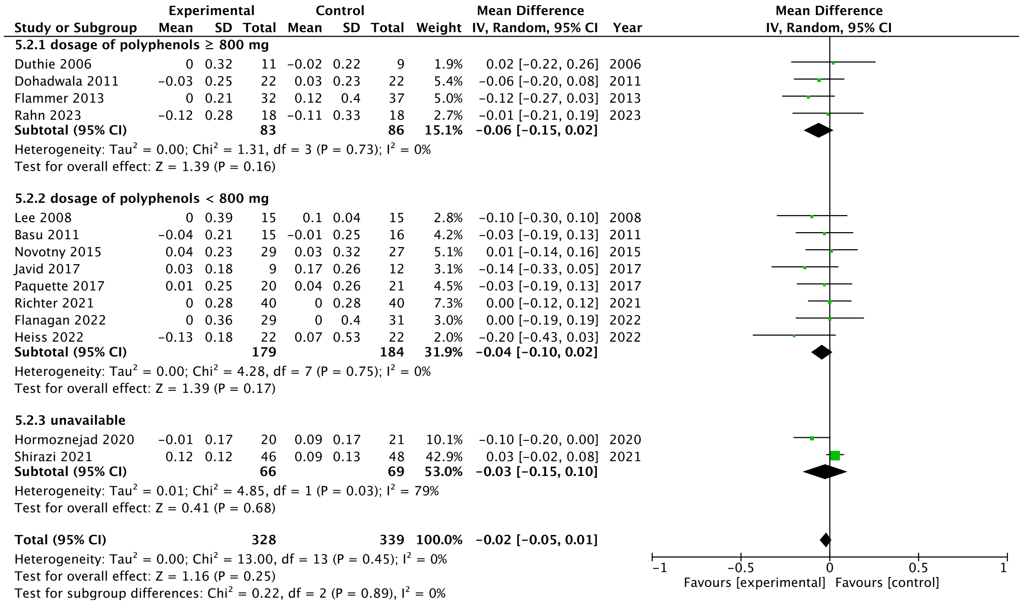

Supplementary Figure 25. Subgroup analysis of the effect of the dosage of total polyphenols on HDL-C.

Study or Subgroup	Experimental Mean	Experimental SD	Experimental Total	Control Mean	Control SD	Control Total	Weight	Mean Difference IV, Random, 95% CI	Year
**5.3.1 dosage of polyphenols ≥ 800 mg**									
Duthie 2006	−0.38	0.65	11	−0.2	0.69	9	4.8%	−0.18 [−0.77, 0.41]	2006
Dohadwala 2011	0	0.85	22	−0.03	0.89	22	5.9%	0.03 [−0.48, 0.54]	2011
Rahn 2023	−0.03	0.5	18	−0.08	0.75	18	7.9%	0.05 [−0.37, 0.47]	2023
**Subtotal (95% CI)**			**51**			**49**	**18.6%**	**−0.01 [−0.29, 0.28]**	
Heterogeneity: $Tau^2 = 0.00$; $Chi^2 = 0.42$, df = 2 (P = 0.81); $I^2 = 0\%$									
Test for overall effect: Z = 0.06 (P = 0.95)									
**5.3.2 dosage of polyphenols < 800 mg**									
Lee 2008	−0.4	0.39	15	0.2	0.39	15	12.4%	−0.60 [−0.88, −0.32]	2008
Basu 2011	−0.13	0.67	15	0.1	0.68	16	6.6%	−0.23 [−0.71, 0.25]	2011
Novotny 2015	0.1	0.51	29	0.03	0.5	27	13.0%	0.07 [−0.19, 0.33]	2015
Javid 2017	0.08	1.39	9	−0.25	0.72	12	1.9%	0.33 [−0.67, 1.33]	2017
Paquette 2017	−0.01	0.76	20	0.17	0.74	21	6.9%	−0.18 [−0.64, 0.28]	2017
Richter 2021	0	0.71	40	−0.08	0.71	40	11.1%	0.08 [−0.23, 0.39]	2021
Flanagan 2022	−0.3	1	29	−0.1	1.08	31	5.7%	−0.20 [−0.73, 0.33]	2022
Heiss 2022	−0.05	0.56	22	0.04	1.53	22	3.8%	−0.09 [−0.77, 0.59]	2022
**Subtotal (95% CI)**			**179**			**184**	**61.5%**	**−0.14 [−0.37, 0.08]**	
Heterogeneity: $Tau^2 = 0.05$; $Chi^2 = 15.81$, df = 7 (P = 0.03); $I^2 = 56\%$									
Test for overall effect: Z = 1.26 (P = 0.21)									
**5.3.3 unavailable**									
Hormoznejad 2020	0.09	1.13	20	−0.23	0.84	21	4.5%	0.32 [−0.29, 0.93]	2020
Shirazi 2021	−0.26	0.53	46	−0.21	0.51	48	15.4%	−0.05 [−0.26, 0.16]	2021
**Subtotal (95% CI)**			**66**			**69**	**19.9%**	**0.02 [−0.26, 0.30]**	
Heterogeneity: $Tau^2 = 0.01$; $Chi^2 = 1.26$, df = 1 (P = 0.26); $I^2 = 20\%$									
Test for overall effect: Z = 0.13 (P = 0.90)									
**Total (95% CI)**			**296**			**302**	**100.0%**	**−0.09 [−0.23, 0.06]**	
Heterogeneity: $Tau^2 = 0.02$; $Chi^2 = 19.29$, df = 12 (P = 0.08); $I^2 = 38\%$									
Test for overall effect: Z = 1.19 (P = 0.23)									
Test for subgroup differences: $Chi^2 = 0.97$, df = 2 (P = 0.62), $I^2 = 0\%$									

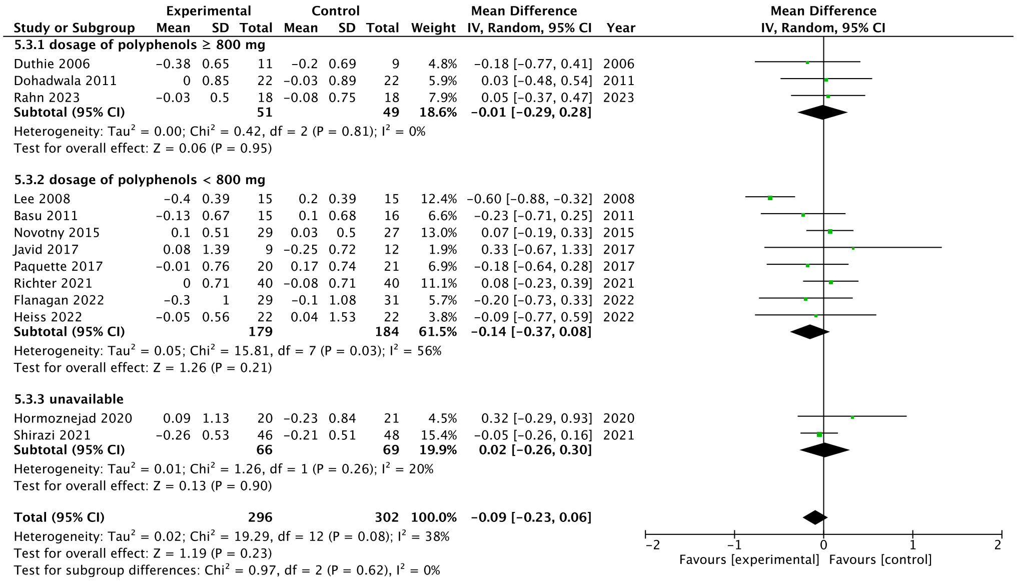

Supplementary Figure 26. Subgroup analysis of the effect of the dosage of total polyphenols on LDL-C.

Study or Subgroup	Experimental Mean	Experimental SD	Experimental Total	Control Mean	Control SD	Control Total	Weight	Mean Difference IV, Random, 95% CI	Year
**5.4.1 dosage of polyphenols ≥ 800 mg**									
Duthie 2006	0.24	0.37	11	−0.01	0.35	9	5.5%	0.25 [−0.07, 0.57]	2006
Dohadwala 2011	−0.02	0.61	22	0.01	0.69	22	4.1%	−0.03 [−0.41, 0.35]	2011
Flammer 2013	0.07	0.31	32	−0.1	0.33	37	13.3%	0.17 [0.02, 0.32]	2013
Rahn 2023	0.09	0.56	18	0.24	0.42	18	5.3%	−0.15 [−0.47, 0.17]	2023
**Subtotal (95% CI)**			**83**			**86**	**28.2%**	**0.10 [−0.07, 0.26]**	
Heterogeneity: $Tau^2 = 0.01$; $Chi^2 = 4.33$, df = 3 (P = 0.23); $I^2 = 31\%$									
Test for overall effect: Z = 1.15 (P = 0.25)									
**5.4.2 dosage of polyphenols < 800 mg**									
Lee 2008	−0.1	0.77	15	0	0.39	15	3.3%	−0.10 [−0.54, 0.34]	2008
Basu 2011	0.07	0.16	15	−0.04	0.11	16	17.5%	0.11 [0.01, 0.21]	2011
Novotny 2015	−0.13	0.47	29	−0.03	0.5	27	7.5%	−0.10 [−0.35, 0.15]	2015
Javid 2017	−0.24	1.01	9	−0.25	0.39	12	1.4%	0.01 [−0.69, 0.71]	2017
Paquette 2017	−0.21	1.01	20	−0.17	1.06	21	1.7%	−0.04 [−0.67, 0.59]	2017
Hsia 2020	−0.16	0.42	18	0.1	0.37	17	7.3%	−0.26 [−0.52, 0.00]	2020
Richter 2021	−0.01	0.43	40	0.06	0.47	40	10.3%	−0.07 [−0.27, 0.13]	2021
Flanagan 2022	0.1	0.4	29	−0.1	0.56	31	7.9%	0.20 [−0.05, 0.45]	2022
Heiss 2022	0.13	0.46	22	−0.07	0.46	22	6.9%	0.20 [−0.07, 0.47]	2022
**Subtotal (95% CI)**			**197**			**201**	**63.8%**	**0.02 [−0.09, 0.13]**	
Heterogeneity: $Tau^2 = 0.01$; $Chi^2 = 12.75$, df = 8 (P = 0.12); $I^2 = 37\%$									
Test for overall effect: Z = 0.31 (P = 0.75)									
**5.4.3 unavailable**									
Hormoznejad 2020	−0.17	0.39	20	−0.55	1.37	21	1.8%	0.38 [−0.23, 0.99]	2020
Shirazi 2021	−0.09	0.74	46	0.08	0.71	48	6.2%	−0.17 [−0.46, 0.12]	2021
**Subtotal (95% CI)**			**66**			**69**	**8.0%**	**0.04 [−0.49, 0.56]**	
Heterogeneity: $Tau^2 = 0.09$; $Chi^2 = 2.53$, df = 1 (P = 0.11); $I^2 = 61\%$									
Test for overall effect: Z = 0.14 (P = 0.89)									
**Total (95% CI)**			**346**			**356**	**100.0%**	**0.04 [−0.05, 0.12]**	
Heterogeneity: $Tau^2 = 0.01$; $Chi^2 = 21.50$, df = 14 (P = 0.09); $I^2 = 35\%$									
Test for overall effect: Z = 0.80 (P = 0.42)									
Test for subgroup differences: $Chi^2 = 0.62$, df = 2 (P = 0.73), $I^2 = 0\%$									

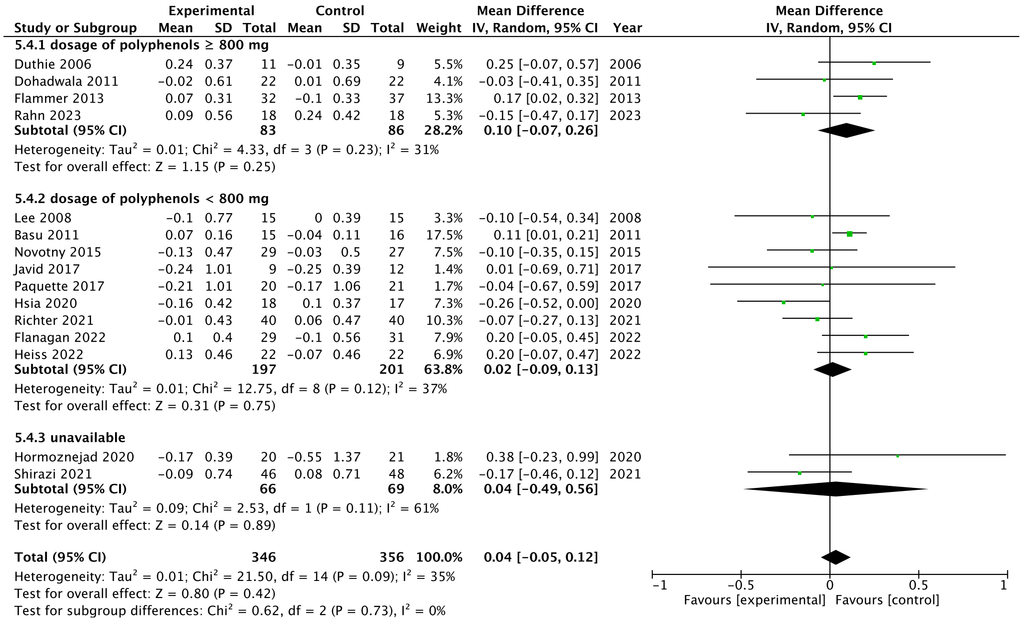

Supplementary Figure 27. Subgroup analysis of the effect of the dosage of total polyphenols on TG.

Study or Subgroup	Experimental Mean	Experimental SD	Experimental Total	Control Mean	Control SD	Control Total	Weight	Mean Difference IV, Random, 95% CI
**5.5.1 dosage of polyphenols ≥ 800 mg**								
Dohadwala 2011	−0.17	2.69	22	0.22	2.69	22	0.7%	−0.39 [−1.98, 1.20]
**Subtotal (95% CI)**			**22**			**22**	**0.7%**	**−0.39 [−1.98, 1.20]**
Heterogeneity: Not applicable								
Test for overall effect: Z = 0.48 (P = 0.63)								
**5.5.2 dosage of polyphenols < 800 mg**								
Basu 2011	0.32	0.45	15	0.06	0.47	16	8.6%	0.26 [−0.06, 0.58]
Flanagan 2022	−0.1	0.4	29	−0.1	0.5	31	11.6%	0.00 [−0.23, 0.23]
Heiss 2022	0.01	0.49	22	0.08	0.6	22	8.6%	−0.07 [−0.39, 0.25]
Hormoznejad 2020	−0.3	0.78	20	0.04	1.07	21		−0.34 [−0.91, 0.23]
Hsia 2020	0.04	0.38	18	−0.02	0.37	17	10.9%	0.06 [−0.19, 0.31]
Javid 2017	−0.17	3.85	9	−1.74	3.59	12	0.2%	1.57 [−1.66, 4.80]
Lee 2008	−0.6	2.32	15	−0.4	1.55	15	0.8%	−0.20 [−1.61, 1.21]
Novotny 2015	−0.1	0.38	29	0.06	0.36	27	12.8%	−0.16 [−0.35, 0.03]
Paquette 2017	0.1	0.45	20	0.1	0.46	21	9.9%	0.00 [−0.28, 0.28]
Richter 2021	0	0.35	40	0.11	0.35	40	14.3%	−0.11 [−0.26, 0.04]
Shirazi 2021	−0.36	0.49	46	−0.3	0.47	48		−0.06 [−0.25, 0.13]
**Subtotal (95% CI)**			**197**			**201**	**77.7%**	**−0.04 [−0.13, 0.04]**
Heterogeneity: $Tau^2 = 0.00$; $Chi^2 = 7.41$, df = 8 (P = 0.49); $I^2 = 0\%$								
Test for overall effect: Z = 1.02 (P = 0.31)								
**5.5.3 unavailable**								
Shidfar 2011	−1.09	0.73	29	0.09	1.22	29	4.8%	−1.18 [−1.70, −0.66]
**Subtotal (95% CI)**			**95**			**98**	**21.7%**	**−0.50 [−1.19, 0.18]**
Heterogeneity: $Tau^2 = 0.32$; $Chi^2 = 15.99$, df = 2 (P = 0.0003); $I^2 = 87\%$								
Test for overall effect: Z = 1.43 (P = 0.15)								
**Total (95% CI)**			**314**			**321**	**100.0%**	**−0.09 [−0.23, 0.04]**
Heterogeneity: $Tau^2 = 0.03$; $Chi^2 = 26.41$, df = 12 (P = 0.009); $I^2 = 55\%$								
Test for overall effect: Z = 1.38 (P = 0.17)								
Test for subgroup differences: $Chi^2 = 1.85$, df = 2 (P = 0.40), $I^2 = 0\%$								

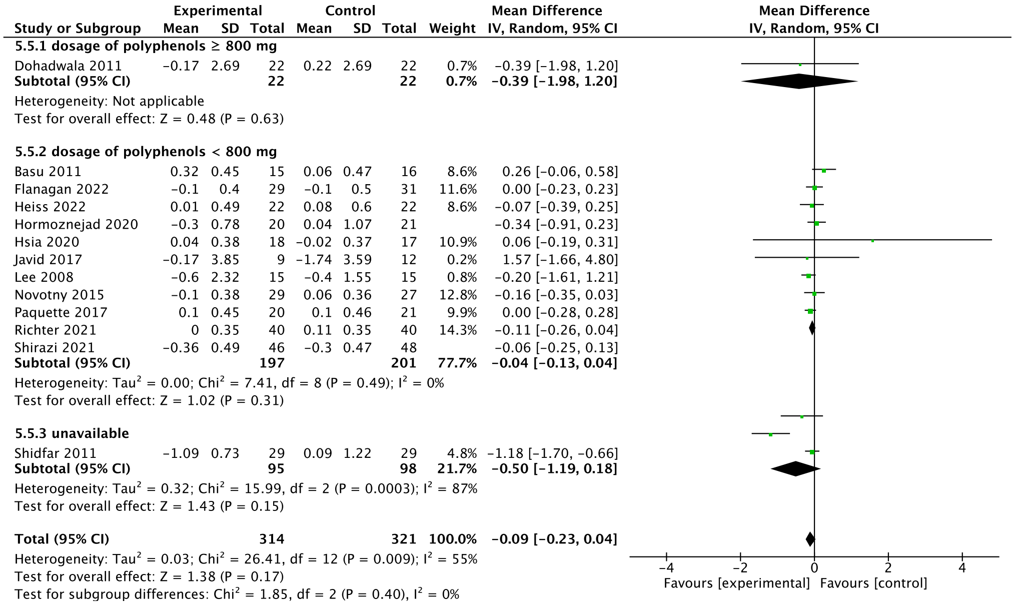

Supplementary Figure 28. Subgroup analysis of the effect of the dosage of total polyphenols on FBG.

Study or Subgroup	Experimental Mean	Experimental SD	Experimental Total	Control Mean	Control SD	Control Total	Weight	Mean Difference IV, Random, 95% CI	Year
**5.6.1 dosage of polyphenols ≥ 800 mg**									
Dohadwala 2011	−1	8	22	0	7.55	22	7.5%	−1.00 [−5.60, 3.60]	2011
**Subtotal (95% CI)**			**22**			**22**	**7.5%**	**−1.00 [−5.60, 3.60]**	
Heterogeneity: Not applicable									
Test for overall effect: Z = 0.43 (P = 0.67)									
**5.6.2 dosage of polyphenols < 800 mg**									
Lee 2008	−0.1	3.1	15	0.4	3.1	15	15.5%	−0.50 [−2.72, 1.72]	2008
Novotny 2015	−0.03	3.42	29	0.18	3.72	27	17.0%	−0.21 [−2.09, 1.67]	2015
Richter 2021	0.2	3.35	40	0.1	3.29	40	18.9%	0.10 [−1.36, 1.56]	2021
**Subtotal (95% CI)**			**84**			**82**	**51.4%**	**−0.12 [−1.14, 0.90]**	
Heterogeneity: $Tau^2 = 0.00$; $Chi^2 = 0.21$, df = 2 (P = 0.90); $I^2 = 0\%$									
Test for overall effect: Z = 0.23 (P = 0.82)									
**5.6.3 unavailable**									
Hormoznejad 2020	−2.35	1.59	20	−0.85	1.62	21	20.9%	−1.50 [−2.48, −0.52]	2020
Shirazi 2021	−4.7	2.72	46	−0.59	2.98	48	20.2%	−4.11 [−5.26, −2.96]	2021
**Subtotal (95% CI)**			**66**			**69**	**41.1%**	**−2.79 [−5.34, −0.23]**	
Heterogeneity: $Tau^2 = 3.11$; $Chi^2 = 11.41$, df = 1 (P = 0.0007); $I^2 = 91\%$									
Test for overall effect: Z = 2.14 (P = 0.03)									
**Total (95% CI)**			**172**			**173**	**100.0%**	**−1.31 [−2.85, 0.22]**	
Heterogeneity: $Tau^2 = 2.68$; $Chi^2 = 26.46$, df = 5 (P < 0.0001); $I^2 = 81\%$									
Test for overall effect: Z = 1.68 (P = 0.09)									
Test for subgroup differences: $Chi^2 = 3.65$, df = 2 (P = 0.16), $I^2 = 45.2\%$									

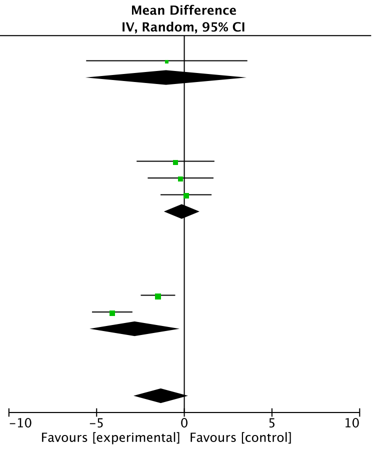

Supplementary Figure 29. Subgroup analysis of the effect of the dosage of total polyphenols on fasting insulin.

Study or Subgroup	Experimental Mean	Experimental SD	Experimental Total	Control Mean	Control SD	Control Total	Weight	Mean Difference IV, Random, 95% CI	Year
**6.1.1 dosage of anthocyanins ≥80 mg**									
Dohadwala 2011	−0.05	1.02	22	0	1.07	22	4.2%	−0.05 [−0.67, 0.57]	2011
Rahn 2023	−0.01	0.24	18	−0.04	0.39	18	14.2%	0.03 [−0.18, 0.24]	2023
**Subtotal (95% CI)**			**40**			**40**	**18.4%**	**0.02 [−0.18, 0.22]**	
Heterogeneity: Tau² = 0.00; Chi² = 0.06, df = 1 (P = 0.81); I² = 0%									
Test for overall effect: Z = 0.21 (P = 0.83)									
**6.1.2 dosage of anthocyanins < 80 mg**									
Duthie 2006	−0.01	0.65	11	−0.03	0.75	9	4.2%	0.02 [−0.60, 0.64]	2006
Lee 2008	−0.4	0.39	15	0.3	0.39	15	11.5%	−0.70 [−0.98, −0.42]	2008
Basu 2011	−0.14	0.85	15	0.13	0.81	16	4.6%	−0.27 [−0.86, 0.32]	2011
Flammer 2013	0.1	0.16	32	0.14	0.18	37	19.4%	−0.04 [−0.12, 0.04]	2013
Novotny 2015	0.08	0.61	29	0	0.73	27	9.1%	0.08 [−0.27, 0.43]	2015
Javid 2017	−0.01	1.07	9	−0.2	0.83	12	2.5%	0.19 [−0.65, 1.03]	2017
Richter 2021	−0.03	0.85	40	−0.03	0.85	40	8.5%	0.00 [−0.37, 0.37]	2021
Flanagan 2022	−0.2	1.15	29	−0.1	1.05	31	4.9%	−0.10 [−0.66, 0.46]	2022
Heiss 2022	−0.13	0.51	22	0.01	1.69	22	3.1%	−0.14 [−0.88, 0.60]	2022
**Subtotal (95% CI)**			**202**			**209**	**67.8%**	**−0.14 [−0.35, 0.07]**	
Heterogeneity: Tau² = 0.05; Chi² = 21.80, df = 8 (P = 0.005); I² = 63%									
Test for overall effect: Z = 1.29 (P = 0.20)									
**6.1.3 unavailable**									
Paquette 2017	−0.1	0.81	20	0.08	0.97	21	5.1%	−0.18 [−0.73, 0.37]	2017
Hormoznejad 2020	−0.04	1.17	20	−0.22	0.74	21	4.4%	0.18 [−0.42, 0.78]	2020
Shirazi 2021	−0.54	1.65	46	−0.13	1.32	48	4.3%	−0.41 [−1.02, 0.20]	2021
**Subtotal (95% CI)**			**86**			**90**	**13.8%**	**−0.14 [−0.48, 0.20]**	
Heterogeneity: Tau² = 0.00; Chi² = 1.87, df = 2 (P = 0.39); I² = 0%									
Test for overall effect: Z = 0.81 (P = 0.42)									
**Total (95% CI)**			**328**			**339**	**100.0%**	**−0.11 [−0.26, 0.03]**	
Heterogeneity: Tau² = 0.03; Chi² = 24.76, df = 13 (P = 0.02); I² = 48%									
Test for overall effect: Z = 1.57 (P = 0.12)									
Test for subgroup differences: Chi² = 1.37, df = 2 (P = 0.50), I² = 0%									

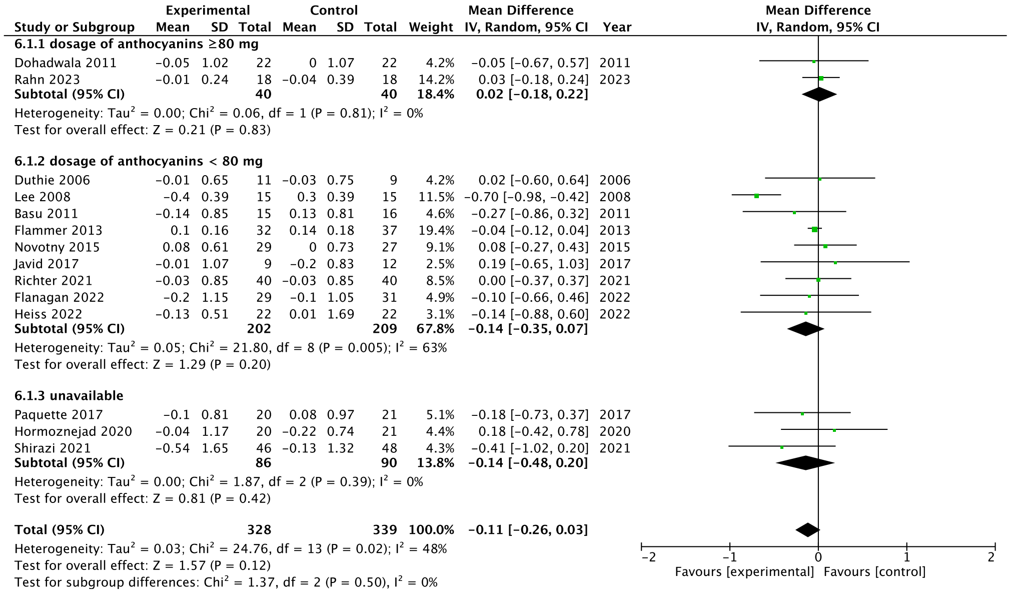

Supplementary Figure 30. Subgroup analysis of the effect of the dosage of anthocyanins on TC.

Study or Subgroup	Experimental Mean	Experimental SD	Experimental Total	Control Mean	Control SD	Control Total	Weight	Mean Difference IV, Random, 95% CI	Year
**6.2.1 dosage of anthocyanins ≥80 mg**									
Dohadwala 2011	−0.03	0.25	22	0.03	0.23	22	5.4%	−0.06 [−0.20, 0.08]	2011
Rahn 2023	−0.12	0.28	18	−0.11	0.33	18	2.7%	−0.01 [−0.21, 0.19]	2023
**Subtotal (95% CI)**			**40**			**40**	**8.2%**	**−0.04 [−0.16, 0.07]**	
Heterogeneity: Tau² = 0.00; Chi² = 0.16, df = 1 (P = 0.69); I² = 0%									
Test for overall effect: Z = 0.73 (P = 0.46)									
**6.2.2 dosage of anthocyanins < 80 mg**									
Duthie 2006	0	0.32	11	−0.02	0.22	9	1.9%	0.02 [−0.22, 0.26]	2006
Lee 2008	0	0.39	15	0.1	0.04	15	2.8%	−0.10 [−0.30, 0.10]	2008
Basu 2011	−0.04	0.21	15	−0.01	0.25	16	4.2%	−0.03 [−0.19, 0.13]	2011
Flammer 2013	0	0.21	32	0.12	0.4	37	5.0%	−0.12 [−0.27, 0.03]	2013
Novotny 2015	0.04	0.23	29	0.03	0.32	27	5.1%	0.01 [−0.14, 0.16]	2015
Javid 2017	0.03	0.18	9	0.17	0.26	12	3.1%	−0.14 [−0.33, 0.05]	2017
Richter 2021	0	0.28	40	0	0.28	40	7.3%	0.00 [−0.12, 0.12]	2021
Flanagan 2022	0	0.36	29	0	0.4	31	3.0%	0.00 [−0.19, 0.19]	2022
Heiss 2022	−0.13	0.18	22	0.07	0.53	22	2.0%	−0.20 [−0.43, 0.03]	2022
**Subtotal (95% CI)**			**202**			**209**	**34.3%**	**−0.05 [−0.11, 0.01]**	
Heterogeneity: Tau² = 0.00; Chi² = 5.49, df = 8 (P = 0.70); I² = 0%									
Test for overall effect: Z = 1.77 (P = 0.08)									
**6.2.3 unavailable**									
Paquette 2017	0.01	0.25	20	0.04	0.26	21	4.5%	−0.03 [−0.19, 0.13]	2017
Hormoznejad 2020	−0.01	0.17	20	0.09	0.17	21	10.1%	−0.10 [−0.20, 0.00]	2020
Shirazi 2021	0.12	0.12	46	0.09	0.13	48	42.9%	0.03 [−0.02, 0.08]	2021
**Subtotal (95% CI)**			**86**			**90**	**57.5%**	**−0.02 [−0.11, 0.07]**	
Heterogeneity: Tau² = 0.00; Chi² = 5.03, df = 2 (P = 0.08); I² = 60%									
Test for overall effect: Z = 0.52 (P = 0.60)									
**Total (95% CI)**			**328**			**339**	**100.0%**	**−0.02 [−0.05, 0.01]**	
Heterogeneity: Tau² = 0.00; Chi² = 13.00, df = 13 (P = 0.45); I² = 0%									
Test for overall effect: Z = 1.16 (P = 0.25)									
Test for subgroup differences: Chi² = 0.25, df = 2 (P = 0.88), I² = 0%									

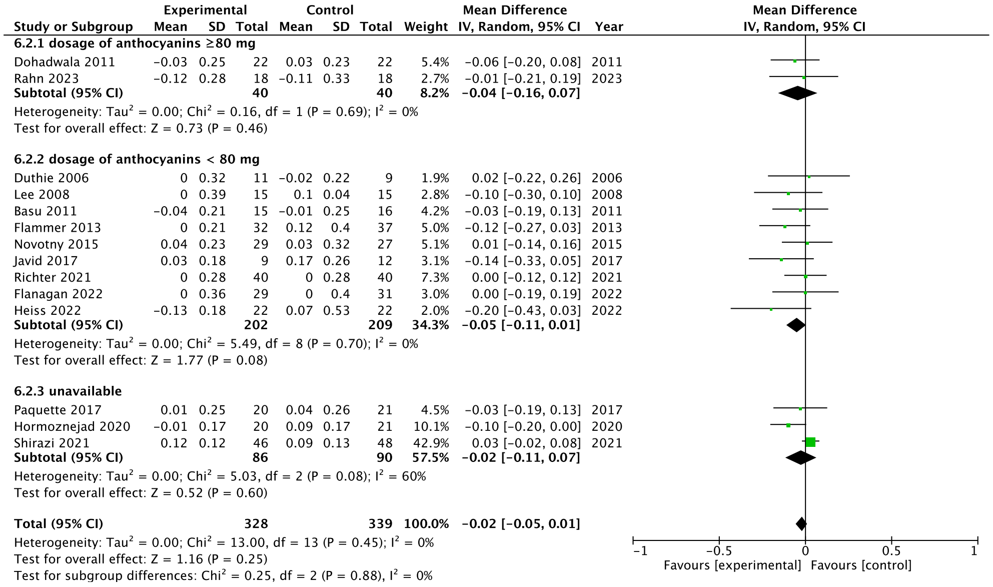

Supplementary Figure 31. Subgroup analysis of the effect of the dosage of anthocyanins on HDL-C.

Study or Subgroup	Experimental Mean	Experimental SD	Experimental Total	Control Mean	Control SD	Control Total	Weight	Mean Difference IV, Random, 95% CI	Year
**6.3.1 dosage of anthocyanins ≥80 mg**									
Dohadwala 2011	0	0.85	22	−0.03	0.89	22	5.9%	0.03 [−0.48, 0.54]	2011
Rahn 2023	−0.03	0.5	18	−0.08	0.75	18	7.9%	0.05 [−0.37, 0.47]	2023
**Subtotal (95% CI)**			**40**			**40**	**13.8%**	**0.04 [−0.28, 0.37]**	
Heterogeneity: Tau² = 0.00; Chi² = 0.00, df = 1 (P = 0.95); I² = 0%									
Test for overall effect: Z = 0.25 (P = 0.80)									
**6.3.2 dosage of anthocyanins < 80 mg**									
Duthie 2006	−0.38	0.65	11	−0.2	0.69	9	4.8%	−0.18 [−0.77, 0.41]	2006
Lee 2008	−0.4	0.39	15	0.2	0.39	15	12.4%	−0.60 [−0.88, −0.32]	2008
Basu 2011	−0.13	0.67	15	0.1	0.68	16	6.6%	−0.23 [−0.71, 0.25]	2011
Novotny 2015	0.1	0.51	29	0.03	0.5	27	13.0%	0.07 [−0.19, 0.33]	2015
Javid 2017	0.08	1.39	9	−0.25	0.72	12	1.9%	0.33 [−0.67, 1.33]	2017
Richter 2021	0	0.71	40	−0.08	0.71	40	11.1%	0.08 [−0.23, 0.39]	2021
Flanagan 2022	−0.3	1	29	−0.1	1.08	31	5.7%	−0.20 [−0.73, 0.33]	2022
Heiss 2022	−0.05	0.56	22	0.04	1.53	22	3.8%	−0.09 [−0.77, 0.59]	2022
**Subtotal (95% CI)**			**170**			**172**	**59.3%**	**−0.14 [−0.38, 0.09]**	
Heterogeneity: Tau² = 0.06; Chi² = 15.81, df = 7 (P = 0.03); I² = 56%									
Test for overall effect: Z = 1.21 (P = 0.23)									
**6.3.3 unavailable**									
Paquette 2017	−0.01	0.76	20	0.17	0.74	21	6.9%	−0.18 [−0.64, 0.28]	2017
Hormoznejad 2020	0.09	1.13	20	−0.23	0.84	21	4.5%	0.32 [−0.29, 0.93]	2020
Shirazi 2021	−0.26	0.53	46	−0.21	0.51	48	15.4%	−0.05 [−0.26, 0.16]	2021
**Subtotal (95% CI)**			**86**			**90**	**26.9%**	**−0.04 [−0.22, 0.15]**	
Heterogeneity: Tau² = 0.00; Chi² = 1.69, df = 2 (P = 0.43); I² = 0%									
Test for overall effect: Z = 0.40 (P = 0.69)									
**Total (95% CI)**			**296**			**302**	**100.0%**	**−0.09 [−0.23, 0.06]**	
Heterogeneity: Tau² = 0.02; Chi² = 19.29, df = 12 (P = 0.08); I² = 38%									
Test for overall effect: Z = 1.19 (P = 0.23)									
Test for subgroup differences: Chi² = 0.94, df = 2 (P = 0.63), I² = 0%									

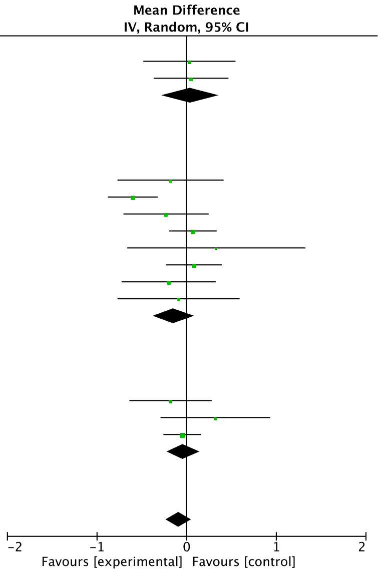

Supplementary Figure 32. Subgroup analysis of the effect of the dosage of anthocyanins on LDL-C.

Study or Subgroup	Experimental Mean	SD	Total	Control Mean	SD	Total	Weight	Mean Difference IV, Random, 95% CI	Year
**6.4.1 dosage of anthocyanins ≥80 mg**									
Dohadwala 2011	−0.02	0.61	22	0.01	0.69	22	4.1%	−0.03 [−0.41, 0.35]	2011
Rahn 2023	0.09	0.56	18	0.24	0.42	18	5.3%	−0.15 [−0.47, 0.17]	2023
**Subtotal (95% CI)**			**40**			**40**	**9.4%**	**−0.10 [−0.35, 0.15]**	
Heterogeneity: Tau^2 = 0.00; Chi^2 = 0.22, df = 1 (P = 0.64); I^2 = 0%									
Test for overall effect: Z = 0.79 (P = 0.43)									
**6.4.2 dosage of anthocyanins < 80 mg**									
Duthie 2006	0.24	0.37	11	−0.01	0.35	9	5.5%	0.25 [−0.07, 0.57]	2006
Lee 2008	−0.1	0.77	15	0	0.39	15	3.3%	−0.10 [−0.54, 0.34]	2008
Basu 2011	0.07	0.16	15	−0.04	0.11	16	17.5%	0.11 [0.01, 0.21]	2011
Flammer 2013	0.07	0.31	32	−0.1	0.33	37	13.3%	0.17 [0.02, 0.32]	2013
Novotny 2015	−0.13	0.47	29	−0.03	0.5	27	7.5%	−0.10 [−0.35, 0.15]	2015
Javid 2017	−0.24	1.01	9	−0.25	0.39	12	1.4%	0.01 [−0.69, 0.71]	2017
Hsia 2020	−0.16	0.42	18	0.1	0.37	17	7.3%	−0.26 [−0.52, 0.00]	2020
Richter 2021	−0.01	0.43	40	0.06	0.47	40	10.3%	−0.07 [−0.27, 0.13]	2021
Heiss 2022	0.13	0.46	22	−0.07	0.46	22	6.9%	0.20 [−0.07, 0.47]	2022
Flanagan 2022	0.1	0.4	29	−0.1	0.56	31	7.9%	0.20 [−0.05, 0.45]	2022
**Subtotal (95% CI)**			**220**			**226**	**80.9%**	**0.06 [−0.04, 0.16]**	
Heterogeneity: Tau^2 = 0.01; Chi^2 = 15.81, df = 9 (P = 0.07); I^2 = 43%									
Test for overall effect: Z = 1.20 (P = 0.23)									
**6.4.3 unavailable**									
Paquette 2017	−0.21	1.01	20	−0.17	1.06	21	1.7%	−0.04 [−0.67, 0.59]	2017
Hormoznejad 2020	−0.17	0.39	20	−0.55	1.37	21	1.8%	0.38 [−0.23, 0.99]	2020
Shirazi 2021	−0.09	0.74	46	0.08	0.71	48	6.2%	−0.17 [−0.46, 0.12]	2021
**Subtotal (95% CI)**			**86**			**90**	**9.7%**	**−0.03 [−0.33, 0.27]**	
Heterogeneity: Tau^2 = 0.02; Chi^2 = 2.54, df = 2 (P = 0.28); I^2 = 21%									
Test for overall effect: Z = 0.19 (P = 0.85)									
**Total (95% CI)**			**346**			**356**	**100.0%**	**0.04 [−0.05, 0.12]**	
Heterogeneity: Tau^2 = 0.01; Chi^2 = 21.50, df = 14 (P = 0.09); I^2 = 35%									
Test for overall effect: Z = 0.80 (P = 0.42)									
Test for subgroup differences: Chi^2 = 1.56, df = 2 (P = 0.46), I^2 = 0%									

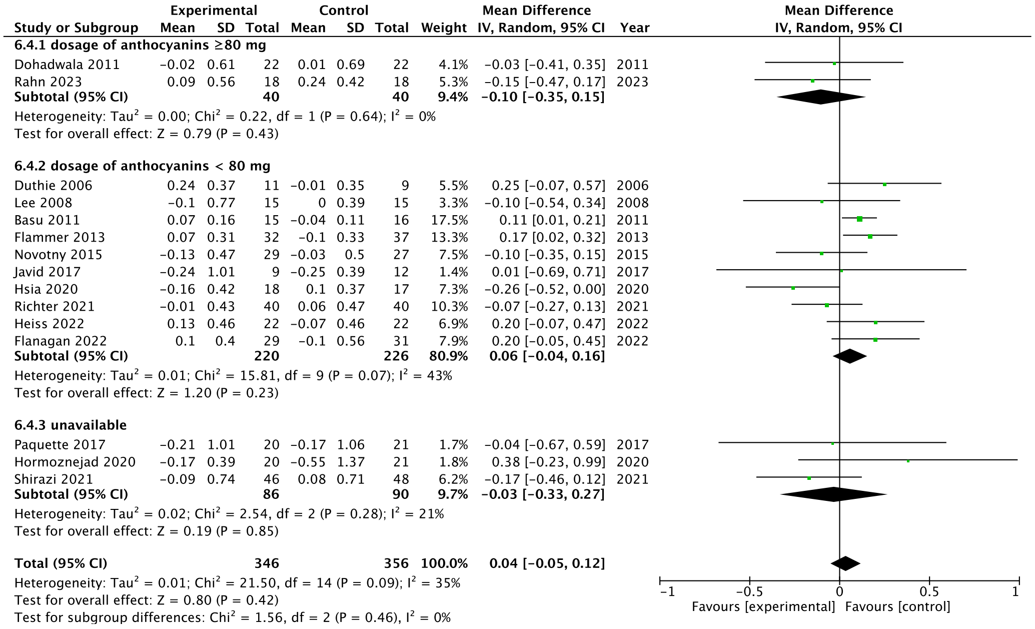

Supplementary Figure 33. Subgroup analysis of the effect of the dosage of anthocyanins on TG.

Study or Subgroup	Experimental Mean	SD	Total	Control Mean	SD	Total	Weight	Mean Difference IV, Random, 95% CI	Year
**6.5.1 dosage of anthocyanins ≥80 mg**									
Dohadwala 2011	−0.17	2.69	22	0.22	2.69	22	0.7%	−0.39 [−1.98, 1.20]	2011
**Subtotal (95% CI)**			**22**			**22**	**0.7%**	**−0.39 [−1.98, 1.20]**	
Heterogeneity: Not applicable									
Test for overall effect: Z = 0.48 (P = 0.63)									
**6.5.2 dosage of anthocyanins < 80 mg**									
Lee 2008	−0.6	2.32	15	−0.4	1.55	15	0.8%	−0.20 [−1.61, 1.21]	2008
Basu 2011	0.32	0.45	15	0.06	0.47	16	8.6%	0.26 [−0.06, 0.58]	2011
Novotny 2015	−0.1	0.38	29	0.06	0.36	27	12.8%	−0.16 [−0.35, 0.03]	2015
Javid 2017	−0.17	3.85	9	−1.74	3.59	12	0.2%	1.57 [−1.66, 4.80]	2017
Hsia 2020	0.04	0.38	18	−0.02	0.37	17	10.9%	0.06 [−0.19, 0.31]	2020
Richter 2021	0	0.35	40	0.11	0.35	40	14.3%	−0.11 [−0.26, 0.04]	2021
Flanagan 2022	−0.1	0.4	29	−0.1	0.5	31	11.6%	0.00 [−0.23, 0.23]	2022
Heiss 2022	0.01	0.49	22	0.08	0.6	22	8.6%	−0.07 [−0.39, 0.25]	2022
**Subtotal (95% CI)**			**177**			**180**	**67.7%**	**−0.05 [−0.14, 0.05]**	
Heterogeneity: Tau^2 = 0.00; Chi^2 = 7.30, df = 7 (P = 0.40); I^2 = 4%									
Test for overall effect: Z = 0.97 (P = 0.33)									
**6.5.3 unavailable**									
Shidfar 2011	−1.09	0.73	29	0.09	1.22	29	4.8%	−1.18 [−1.70, −0.66]	2011
Paquette 2017	0.1	0.45	20	0.1	0.46	21	9.9%	0.00 [−0.28, 0.28]	2017
Hormoznejad 2020	−0.3	0.78	20	0.04	1.07	21	4.1%	−0.34 [−0.91, 0.23]	2020
Shirazi 2021	−0.36	0.49	46	−0.3	0.47	48	12.8%	−0.06 [−0.25, 0.13]	2021
**Subtotal (95% CI)**			**115**			**119**	**31.6%**	**−0.34 [−0.76, 0.08]**	
Heterogeneity: Tau^2 = 0.14; Chi^2 = 17.58, df = 3 (P = 0.0005); I^2 = 83%									
Test for overall effect: Z = 1.60 (P = 0.11)									
**Total (95% CI)**			**314**			**321**	**100.0%**	**−0.09 [−0.23, 0.04]**	
Heterogeneity: Tau^2 = 0.03; Chi^2 = 26.41, df = 12 (P = 0.009); I^2 = 55%									
Test for overall effect: Z = 1.38 (P = 0.17)									
Test for subgroup differences: Chi^2 = 1.98, df = 2 (P = 0.37), I^2 = 0%									

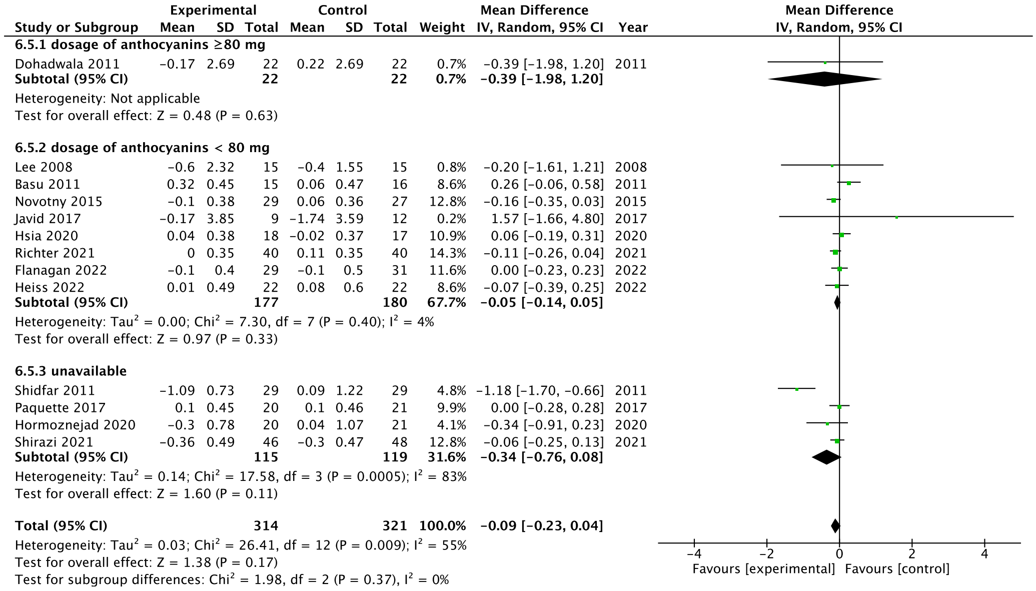

Supplementary Figure 34. Subgroup analysis of the effect of the dosage of anthocyanins on FBG.

Study or Subgroup	Experimental Mean	SD	Total	Control Mean	SD	Total	Weight	Mean Difference IV, Random, 95% CI	Year
**6.6.1 dosage of anthocyanins ≥80 mg**									
Dohadwala 2011	−1	8	22	0	7.55	22	7.5%	−1.00 [−5.60, 3.60]	2011
**Subtotal (95% CI)**			**22**			**22**	**7.5%**	**−1.00 [−5.60, 3.60]**	
Heterogeneity: Not applicable									
Test for overall effect: Z = 0.43 (P = 0.67)									
**6.6.2 dosage of anthocyanins < 80 mg**									
Lee 2008	−0.1	3.1	15	0.4	3.1	15	15.5%	−0.50 [−2.72, 1.72]	2008
Novotny 2015	−0.03	3.42	29	0.18	3.72	27	17.0%	−0.21 [−2.09, 1.67]	2015
Richter 2021	0.2	3.35	40	0.1	3.29	40	18.9%	0.10 [−1.36, 1.56]	2021
**Subtotal (95% CI)**			**84**			**82**	**51.4%**	**−0.12 [−1.14, 0.90]**	
Heterogeneity: Tau^2 = 0.00; Chi^2 = 0.21, df = 2 (P = 0.90); I^2 = 0%									
Test for overall effect: Z = 0.23 (P = 0.82)									
**6.6.3 unavailable**									
Hormoznejad 2020	−2.35	1.59	20	−0.85	1.62	21	20.9%	−1.50 [−2.48, −0.52]	2020
Shirazi 2021	−4.7	2.72	46	−0.59	2.98	48	20.2%	−4.11 [−5.26, −2.96]	2021
**Subtotal (95% CI)**			**66**			**69**	**41.1%**	**−2.79 [−5.34, −0.23]**	
Heterogeneity: Tau^2 = 3.11; Chi^2 = 11.41, df = 1 (P = 0.0007); I^2 = 91%									
Test for overall effect: Z = 2.14 (P = 0.03)									
**Total (95% CI)**			**172**			**173**	**100.0%**	**−1.31 [−2.85, 0.22]**	
Heterogeneity: Tau^2 = 2.68; Chi^2 = 26.46, df = 5 (P < 0.0001); I^2 = 81%									
Test for overall effect: Z = 1.68 (P = 0.09)									
Test for subgroup differences: Chi^2 = 3.65, df = 2 (P = 0.16), I^2 = 45.2%									

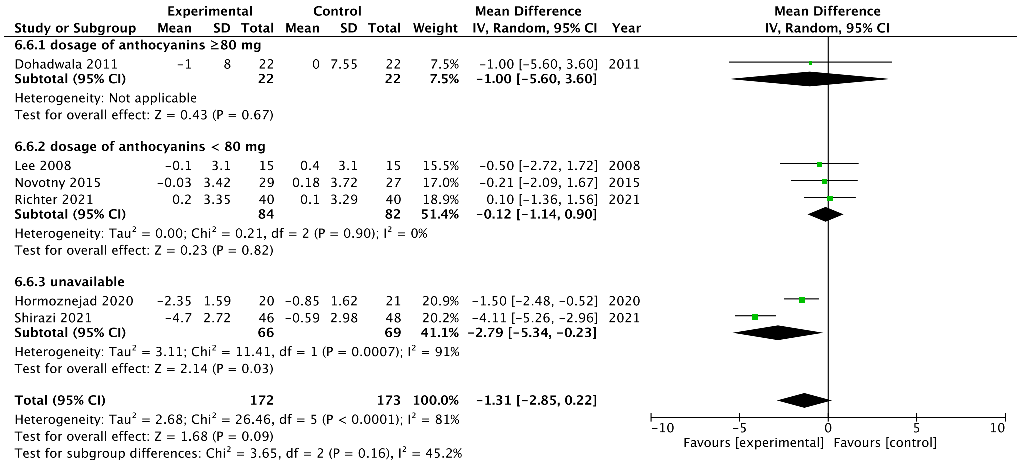

Supplementary Figure 35. Subgroup analysis of the effect of the dosage of anthocyanins on fasting insulin.
