# Supplementary figures and images for: The Effects of Cranberry Consumption on Glycemic and Lipid Profiles in Humans: A Systematic Review and Meta-Analysis of Randomized Controlled Trials
Source: Nutrients. 2024 Mar 9;16(6):782. doi: 10.3390/nu16060782 (PMC10974925; doi:10.3390/nu16060782)

Funnel plot with pseudo 95% confidence limits

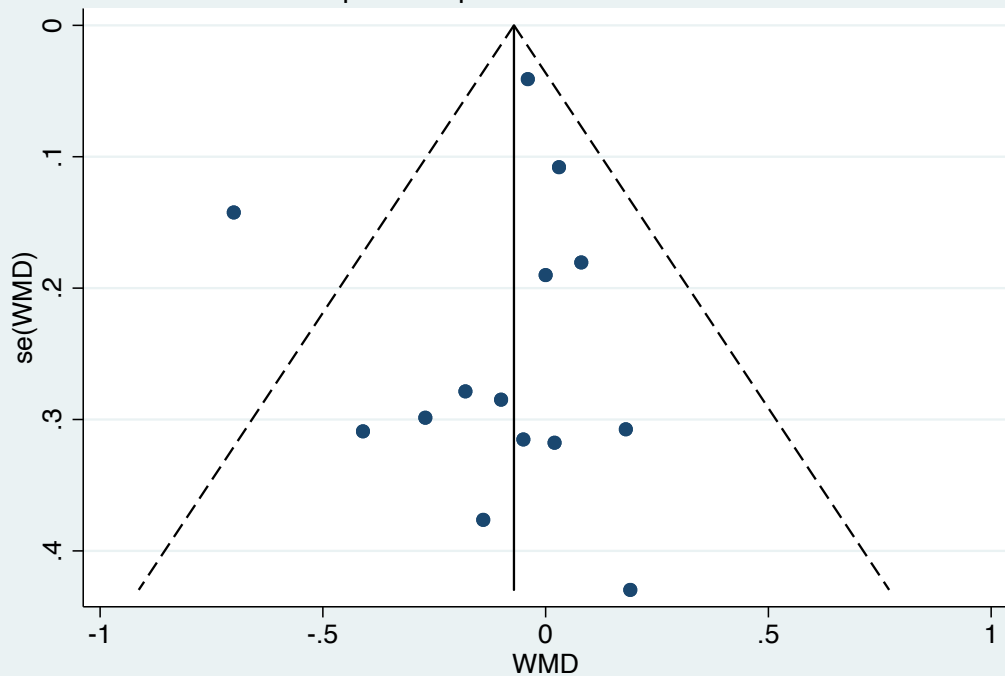

Supplement: Supplementary file 1 [file nutrients-16-00782-s001.zip › Supplementary Figure 1. Funnel plot to evaluate the publication bias for TC.pdf]

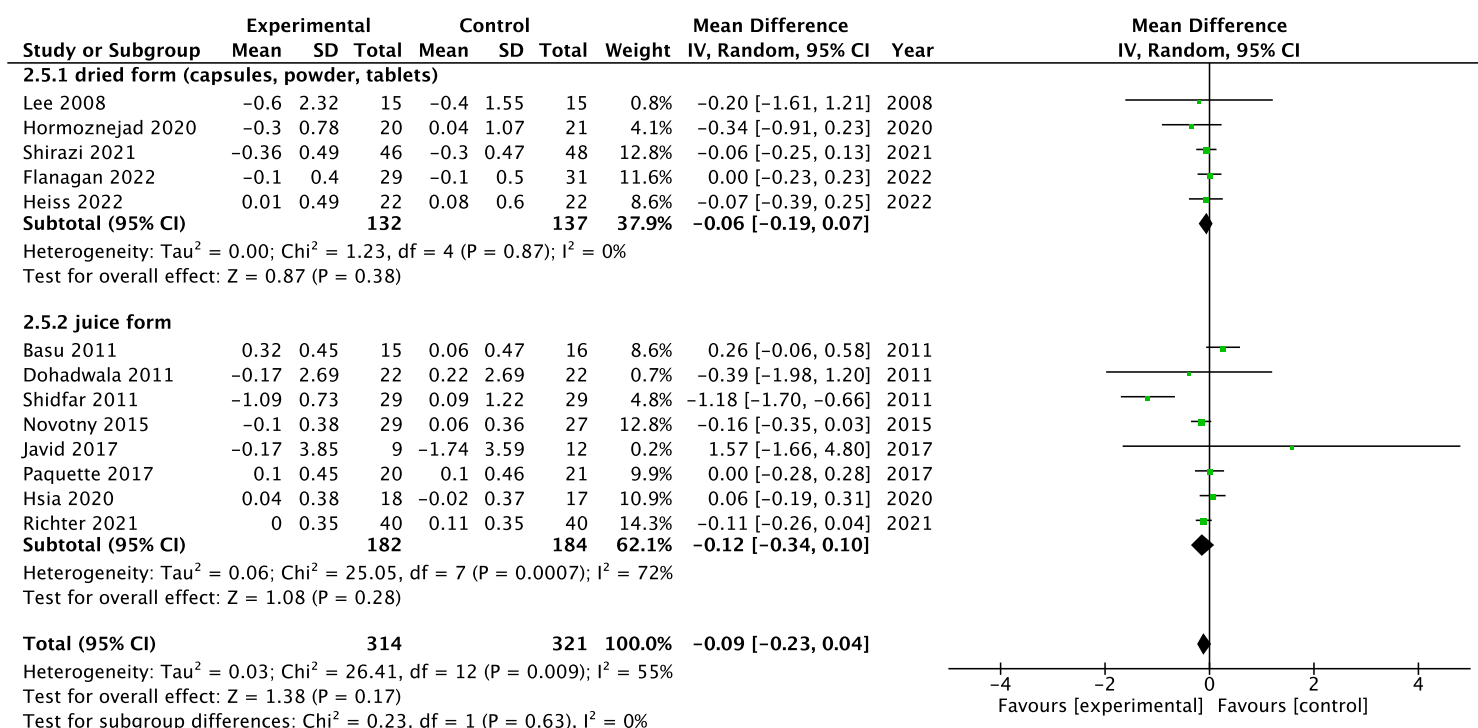

Supplement: Supplementary file 1 [file nutrients-16-00782-s001.zip › Supplementary Figure 10. Subgroup analysis of the effect of dosage form on FBG.pdf]

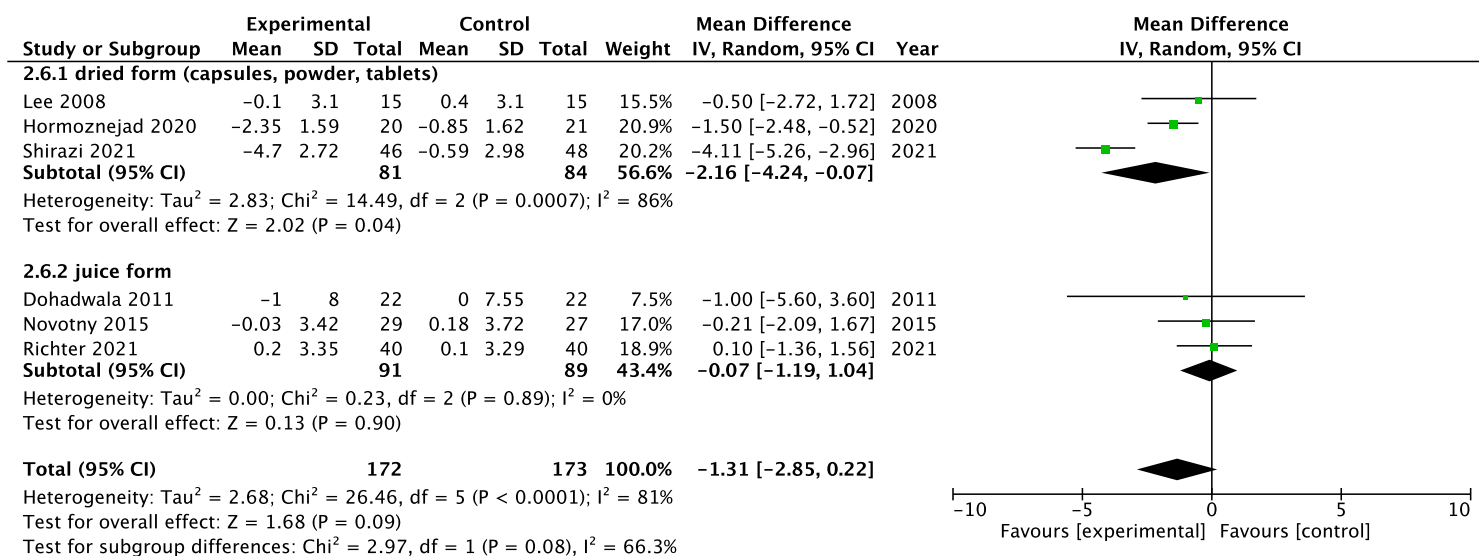

Supplement: Supplementary file 1 [file nutrients-16-00782-s001.zip › Supplementary Figure 11. Subgroup analysis of the effect of dosage form on fasting insulin.pdf]

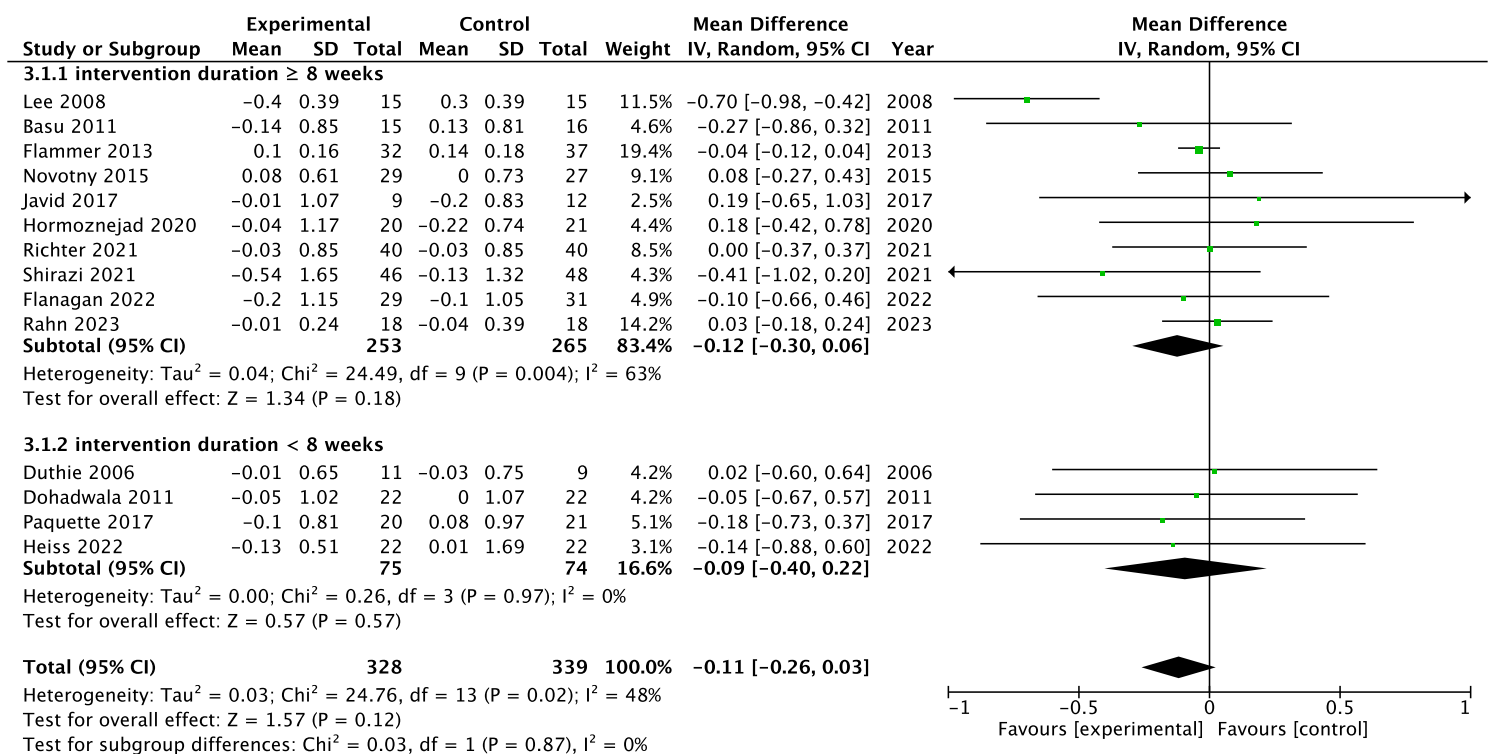

Supplement: Supplementary file 1 [file nutrients-16-00782-s001.zip › Supplementary Figure 12. Subgroup analysis of the effect of intervention duration on TC.pdf]

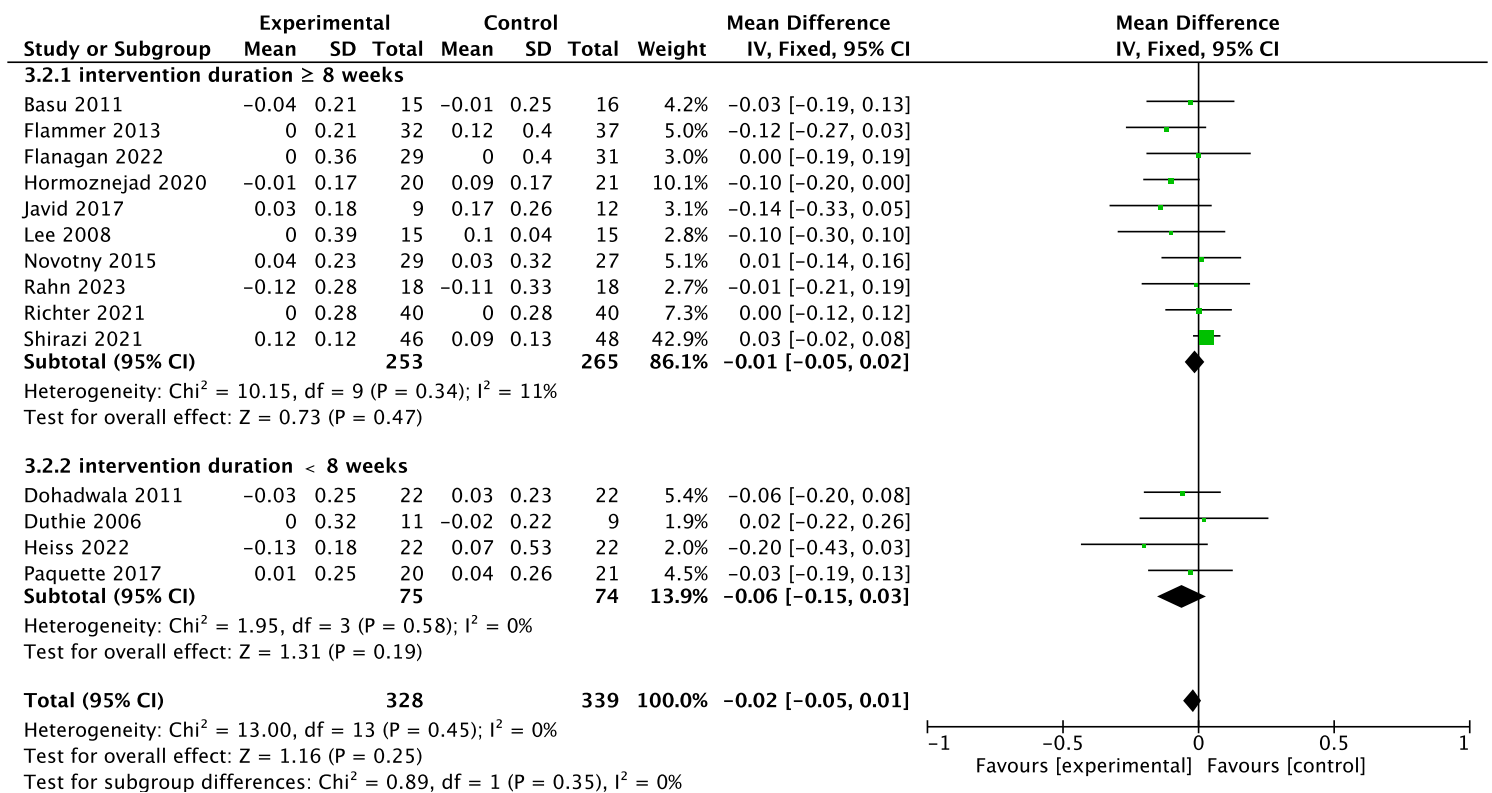

Supplement: Supplementary file 1 [file nutrients-16-00782-s001.zip › Supplementary Figure 13. Subgroup analysis of the effect of intervention duration on HDL-C.pdf]

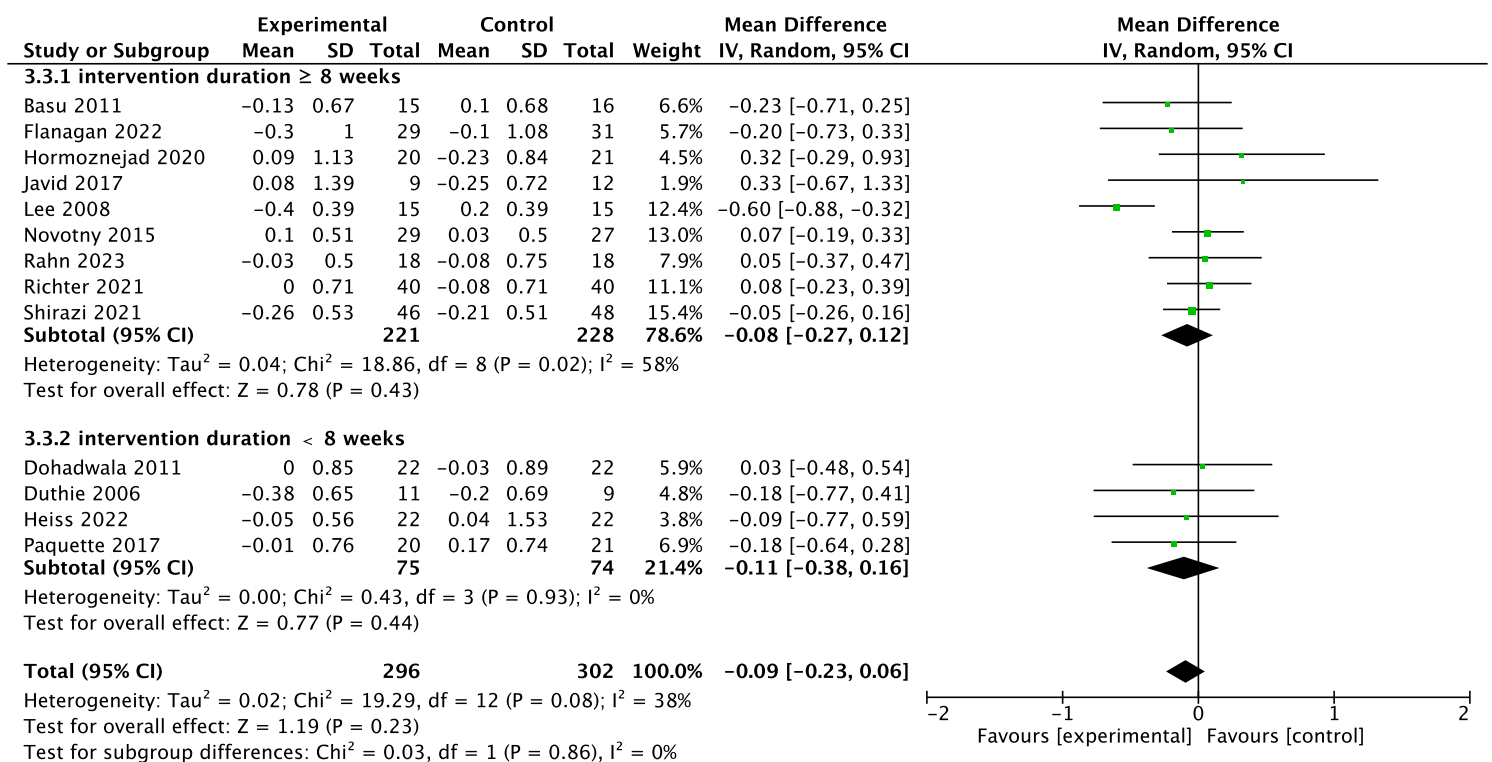

Supplement: Supplementary file 1 [file nutrients-16-00782-s001.zip › Supplementary Figure 14. Subgroup analysis of the effect of intervention duration on LDL-C.pdf]

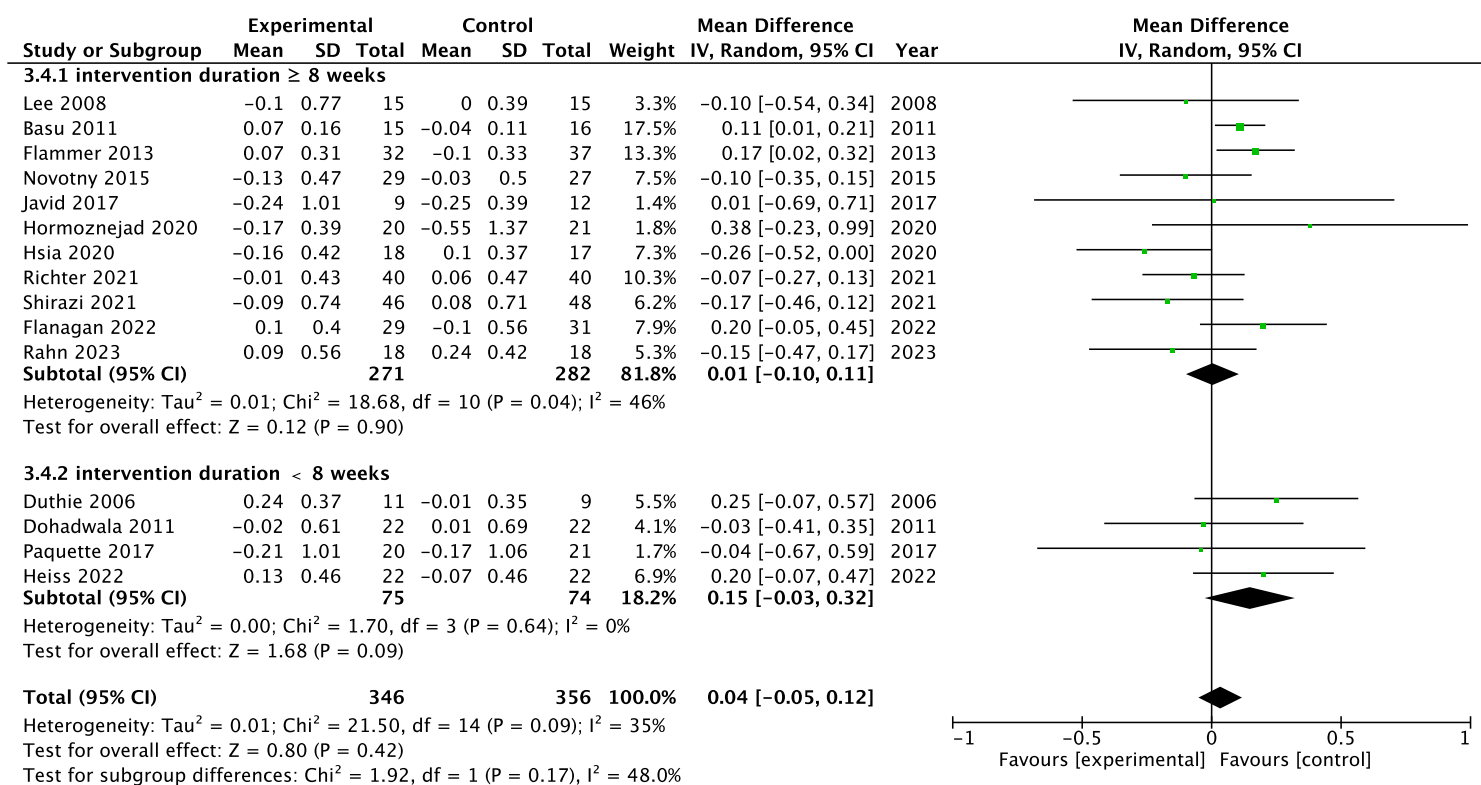

Supplement: Supplementary file 1 [file nutrients-16-00782-s001.zip › Supplementary Figure 15. Subgroup analysis of the effect of intervention duration on TG.pdf]

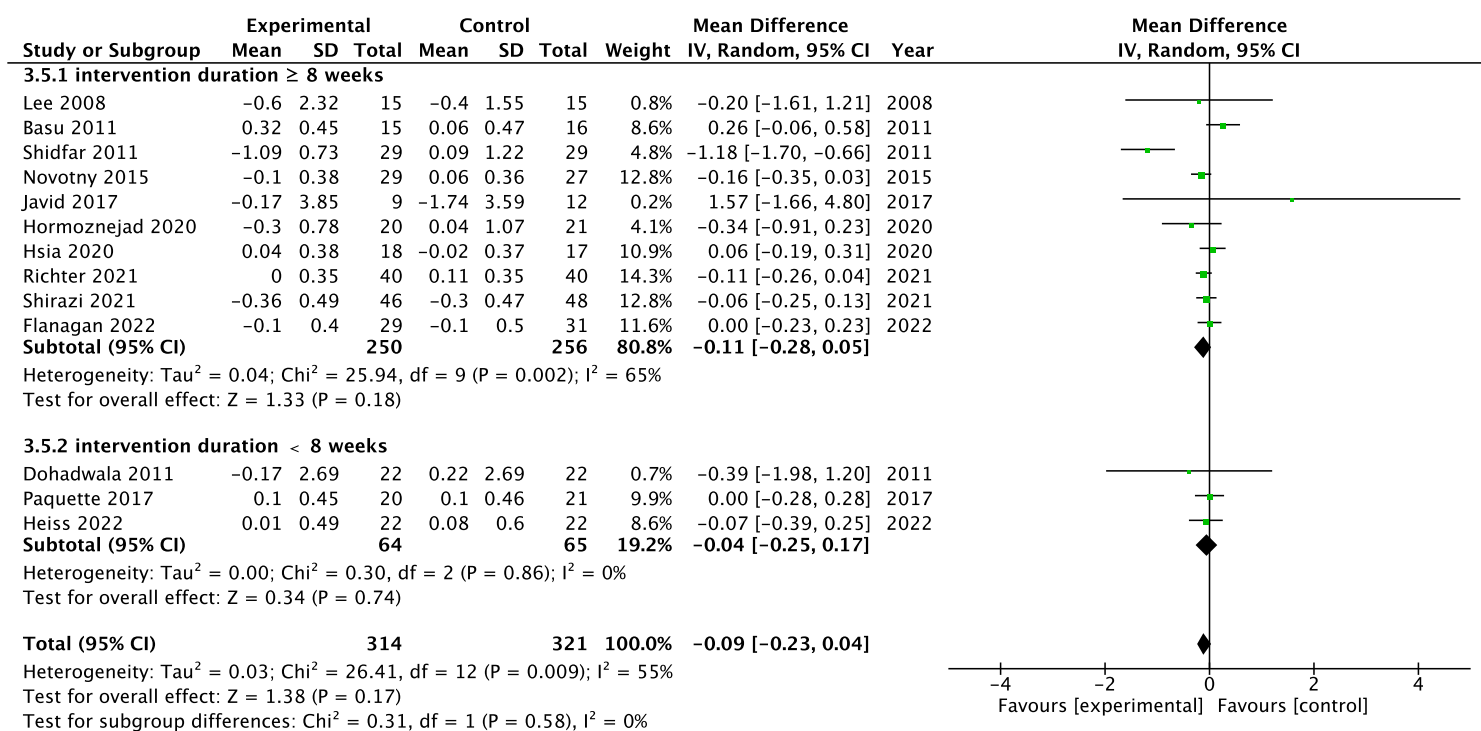

Supplement: Supplementary file 1 [file nutrients-16-00782-s001.zip › Supplementary Figure 16. Subgroup analysis of the effect of intervention duration on FBG.pdf]

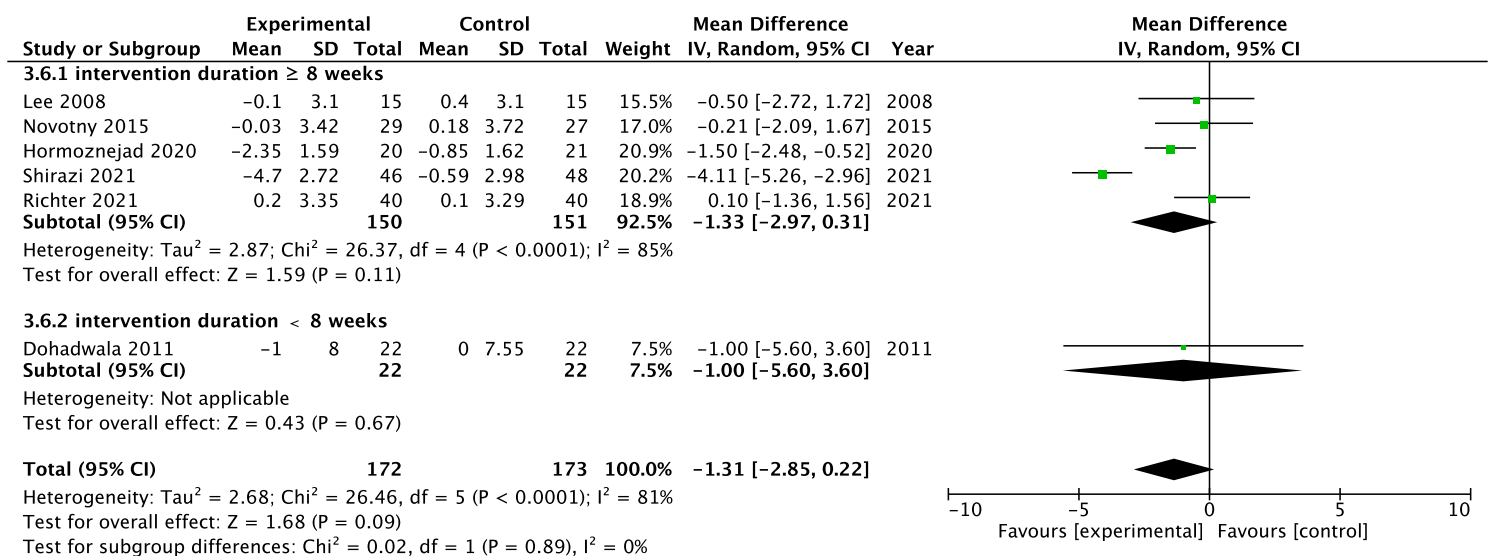

Supplement: Supplementary file 1 [file nutrients-16-00782-s001.zip › Supplementary Figure 17. Subgroup analysis of the effect of intervention duration on fasting insulin.pdf]

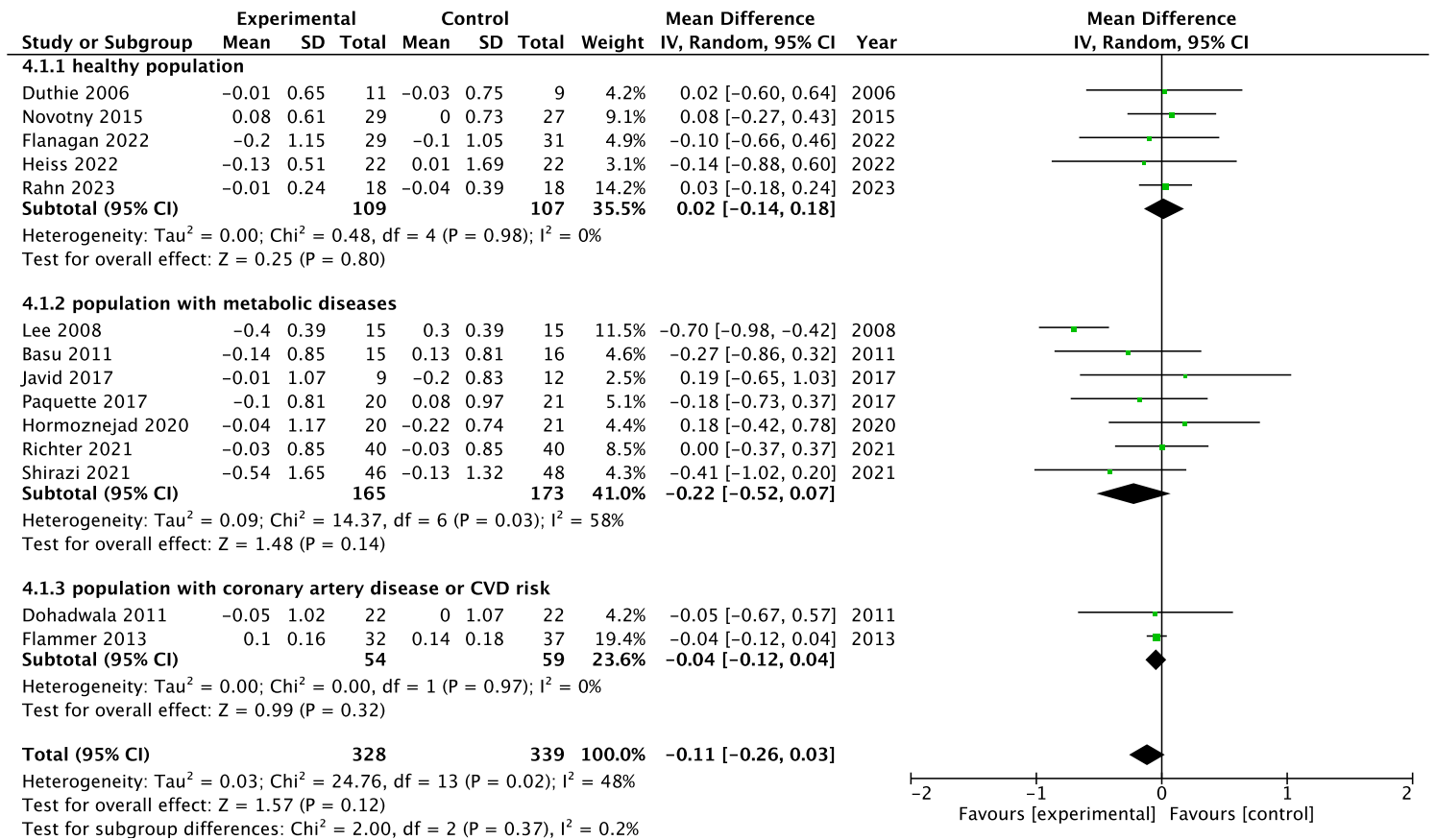

Supplement: Supplementary file 1 [file nutrients-16-00782-s001.zip › Supplementary Figure 18. Subgroup analysis of the effect of health conditions on TC.pdf]

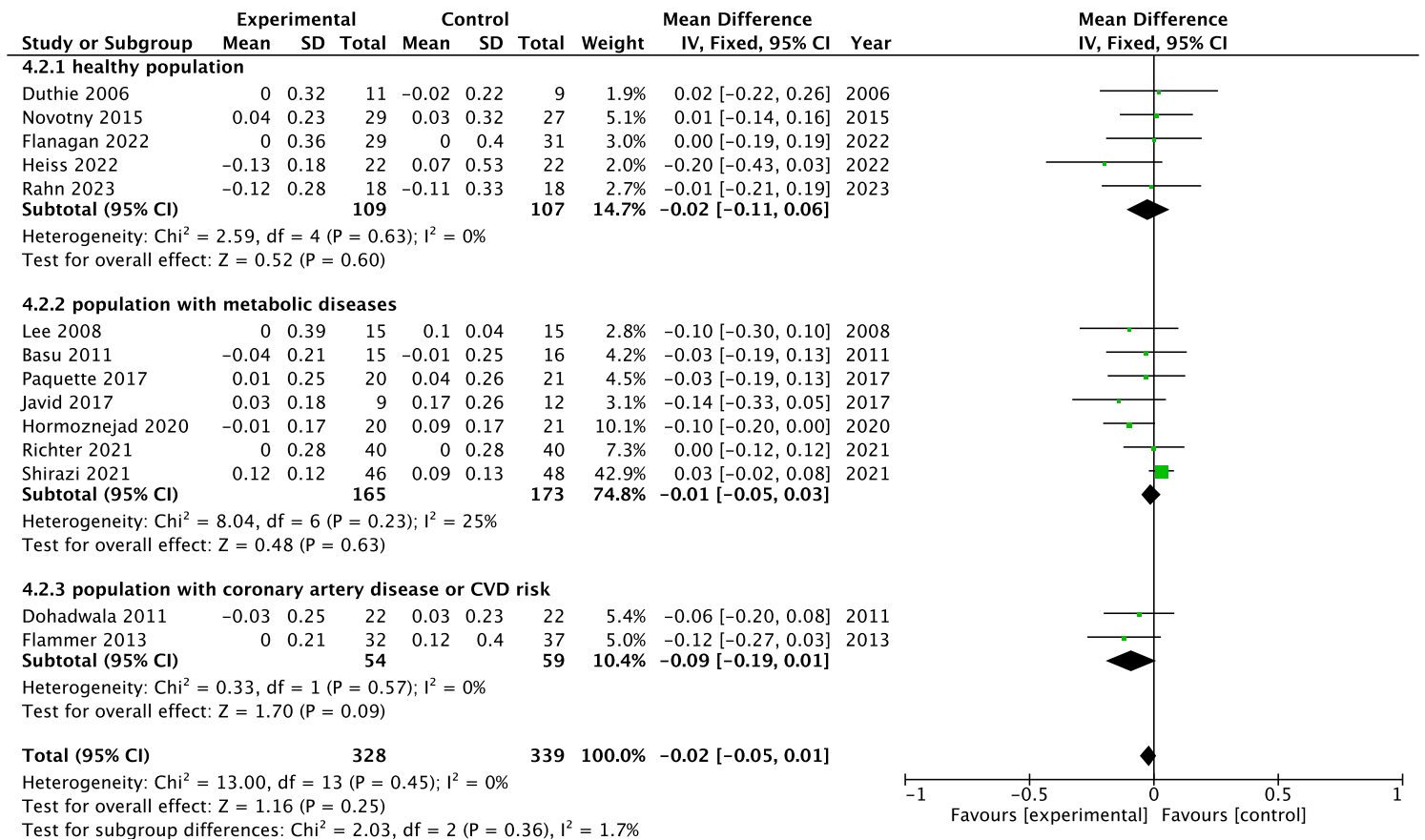

Supplement: Supplementary file 1 [file nutrients-16-00782-s001.zip › Supplementary Figure 19. Subgroup analysis of the effect of health conditions on HDL-C.pdf]

Funnel plot with pseudo 95% confidence limits

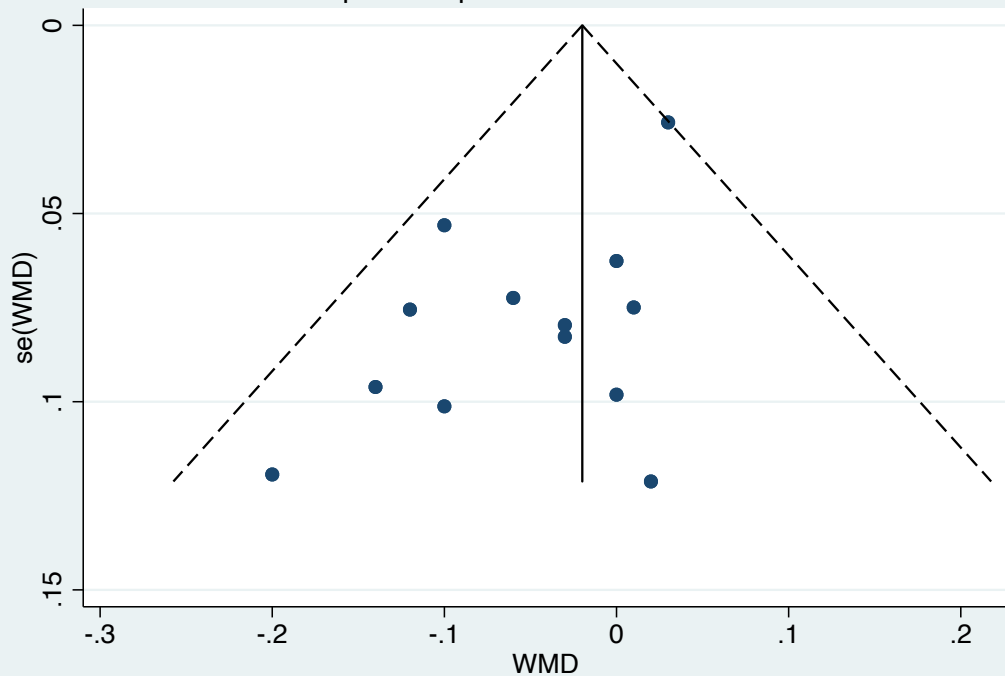

Supplement: Supplementary file 1 [file nutrients-16-00782-s001.zip › Supplementary Figure 2. Funnel plot to evaluate the publication bias for HDL-C.pdf]

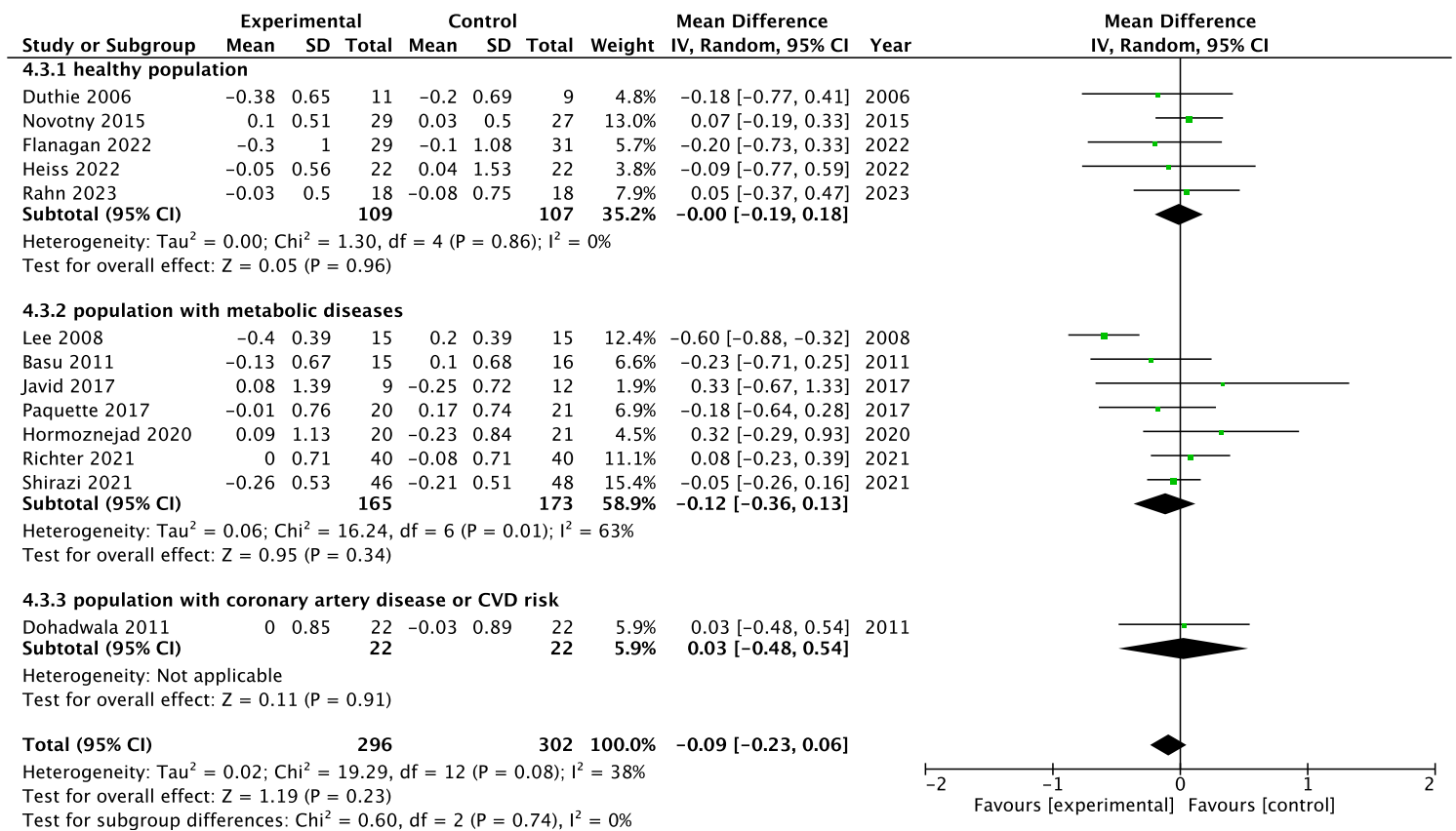

Supplement: Supplementary file 1 [file nutrients-16-00782-s001.zip › Supplementary Figure 20. Subgroup analysis of the effect of health conditions on LDL-C.pdf]

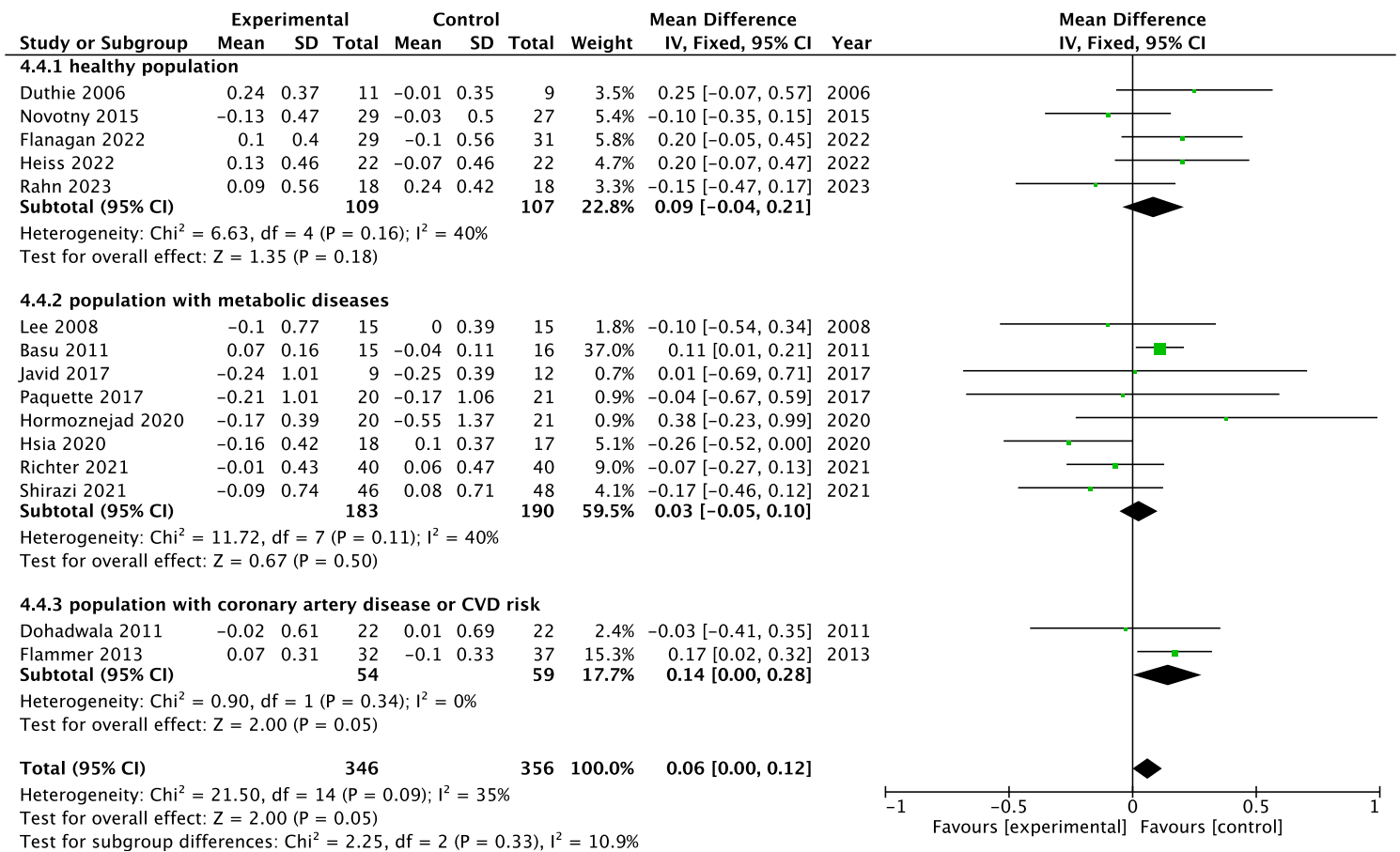

Supplement: Supplementary file 1 [file nutrients-16-00782-s001.zip › Supplementary Figure 21. Subgroup analysis of the effect of health conditions on TG.pdf]

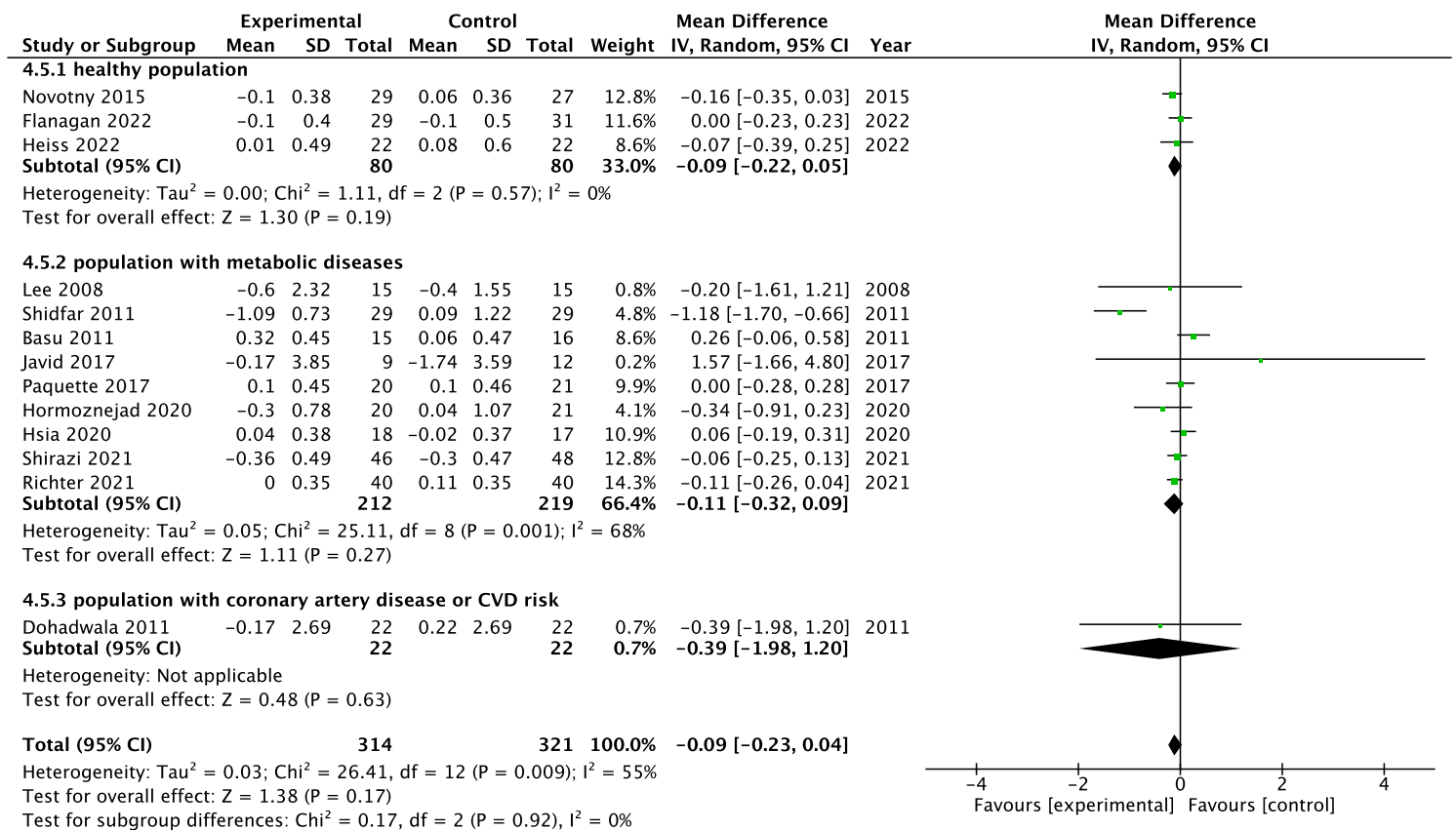

Supplement: Supplementary file 1 [file nutrients-16-00782-s001.zip › Supplementary Figure 22. Subgroup analysis of the effect of health conditions on FBG.pdf]

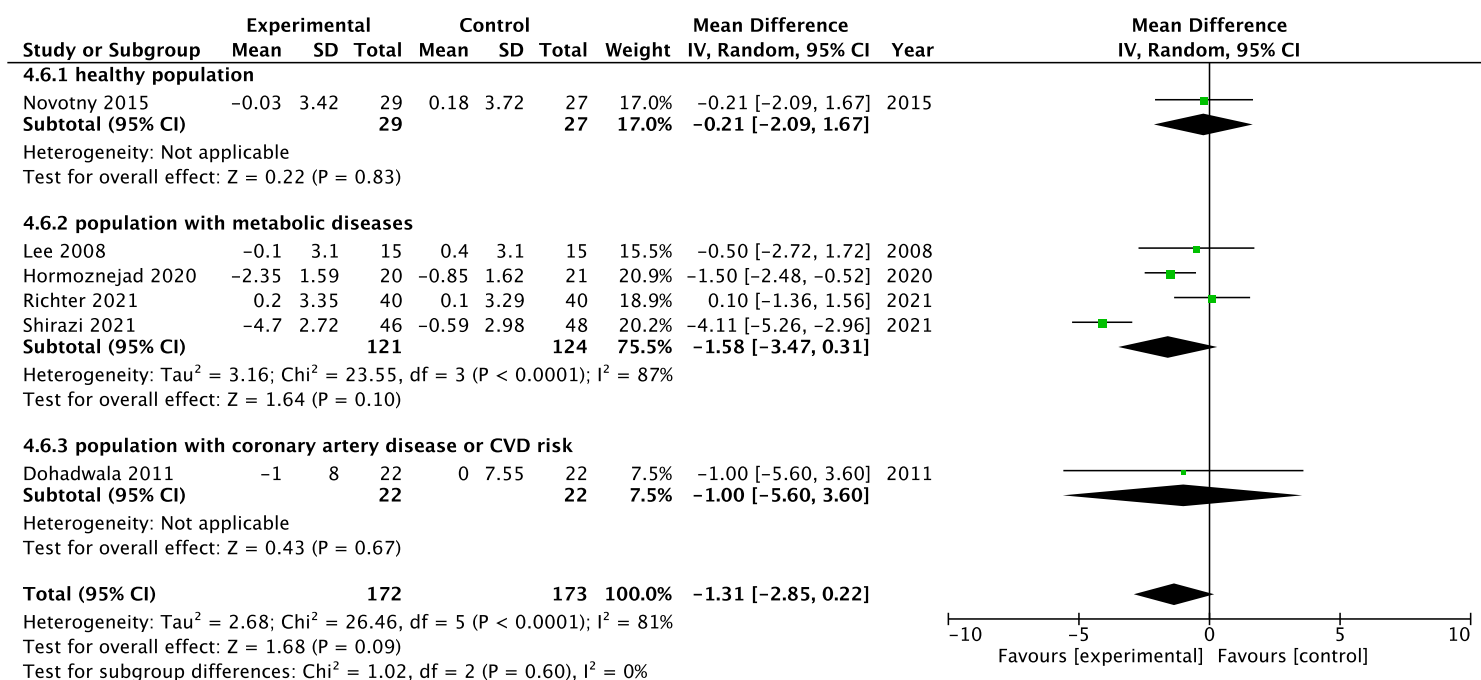

Supplement: Supplementary file 1 [file nutrients-16-00782-s001.zip › Supplementary Figure 23. Subgroup analysis of the effect of health conditions on fasting insulin.pdf]

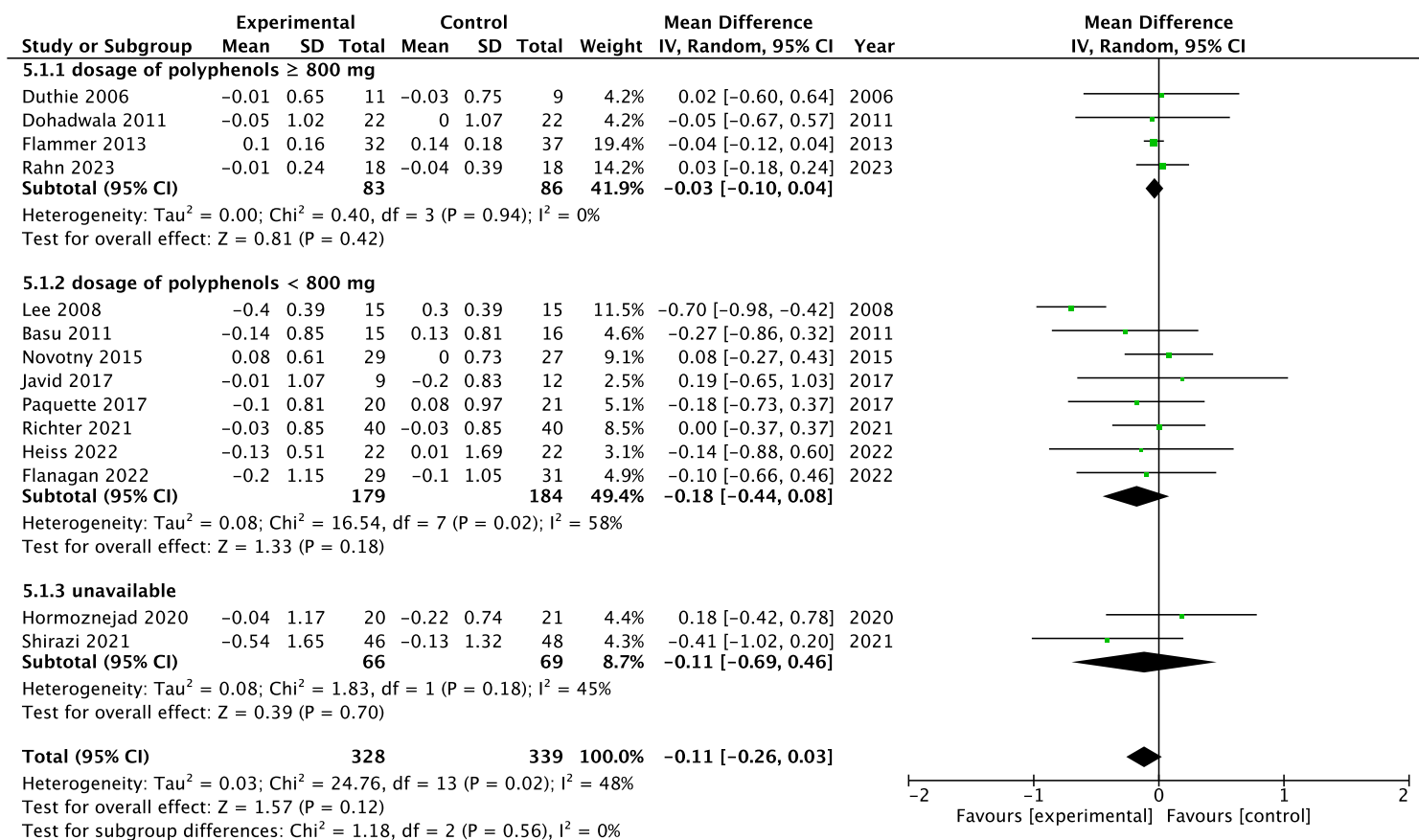

Supplement: Supplementary file 1 [file nutrients-16-00782-s001.zip › Supplementary Figure 24. Subgroup analysis of the effect of the dosage of total polyphenols on TC.pdf]

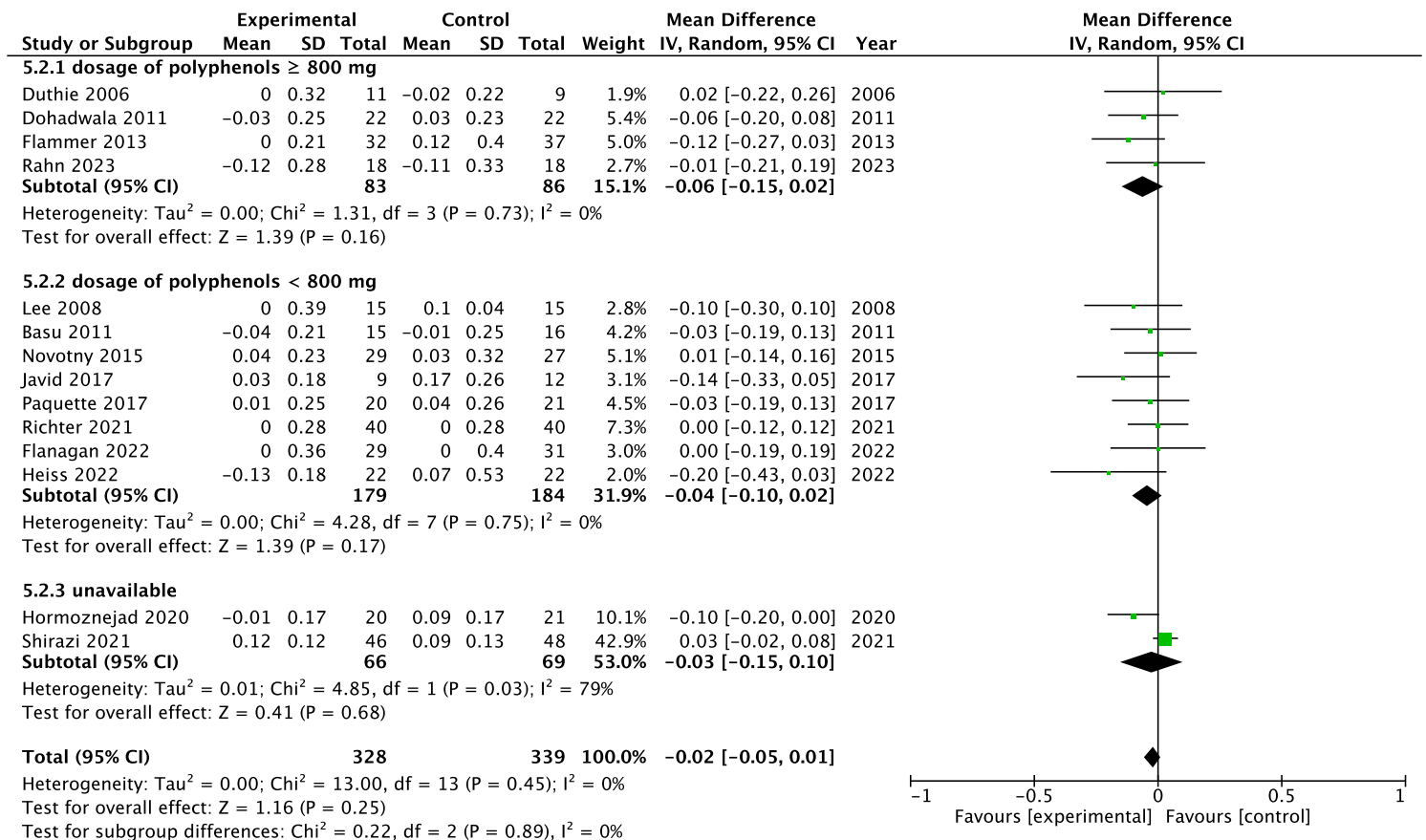

Supplement: Supplementary file 1 [file nutrients-16-00782-s001.zip › Supplementary Figure 25. Subgroup analysis of the effect of the dosage of total polyphenols on HDL-C.pdf]

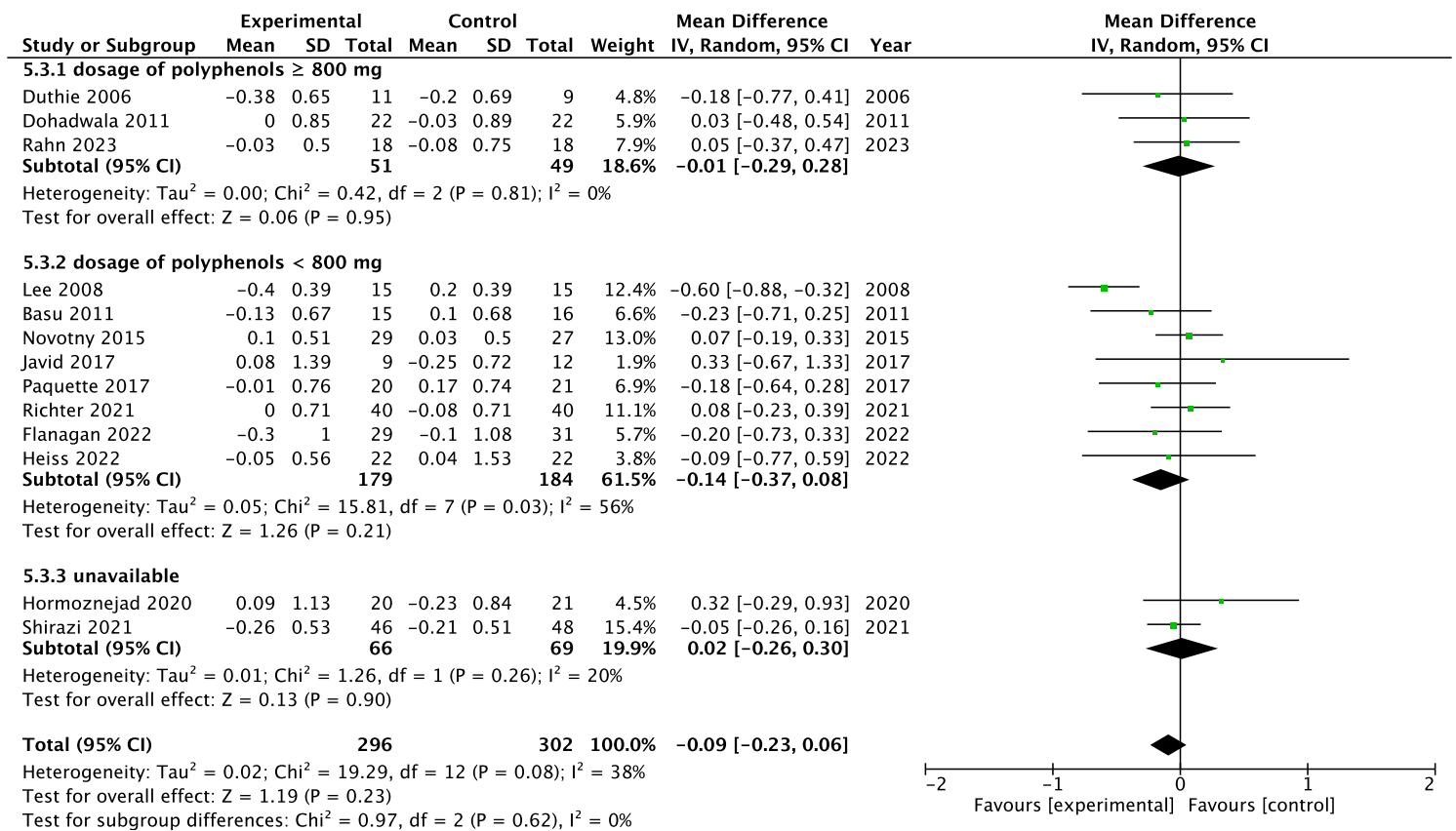

Supplement: Supplementary file 1 [file nutrients-16-00782-s001.zip › Supplementary Figure 26. Subgroup analysis of the effect of the dosage of total polyphenols on LDL-C.pdf]

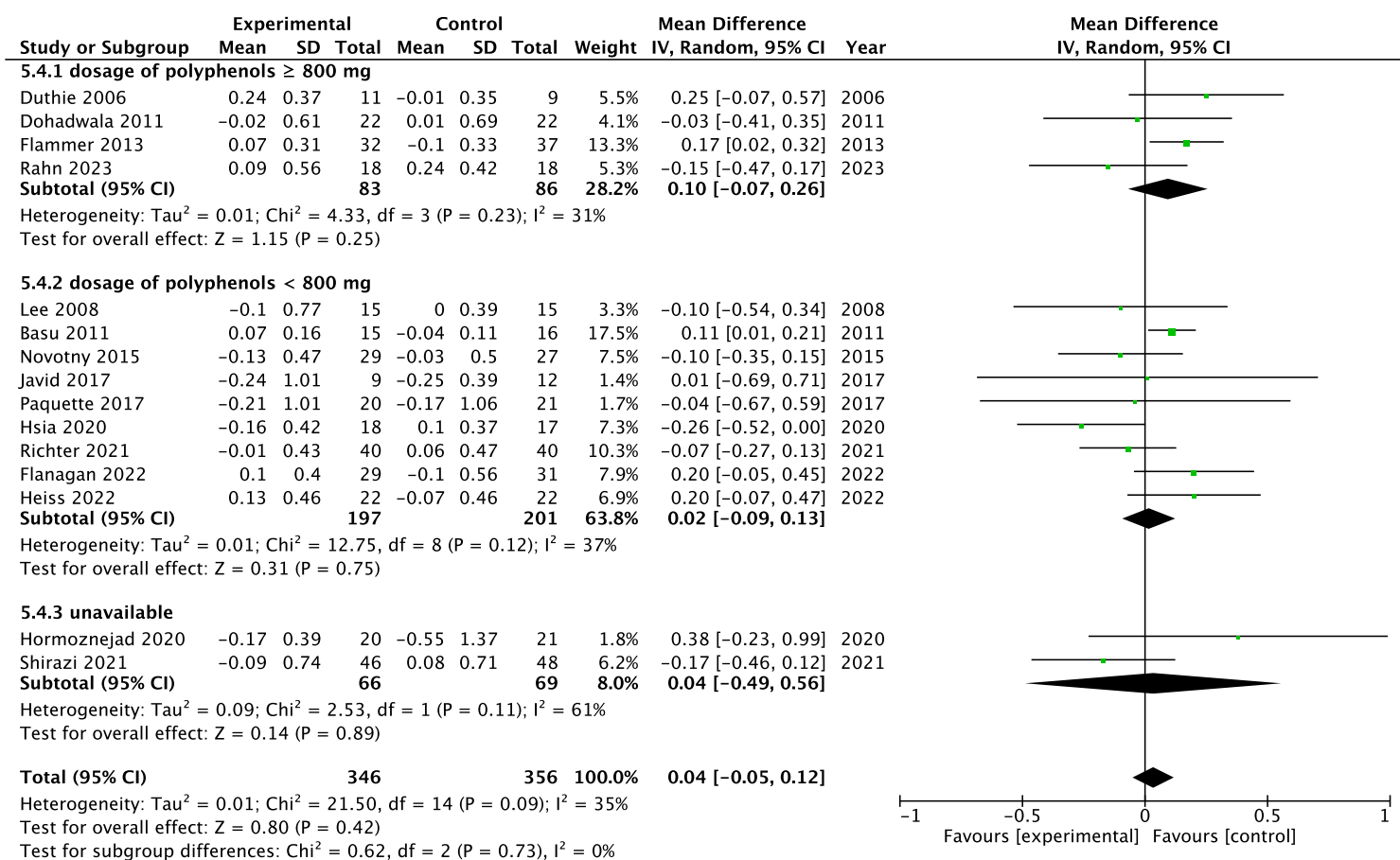

Supplement: Supplementary file 1 [file nutrients-16-00782-s001.zip › Supplementary Figure 27. Subgroup analysis of the effect of the dosage of total polyphenols on TG.pdf]

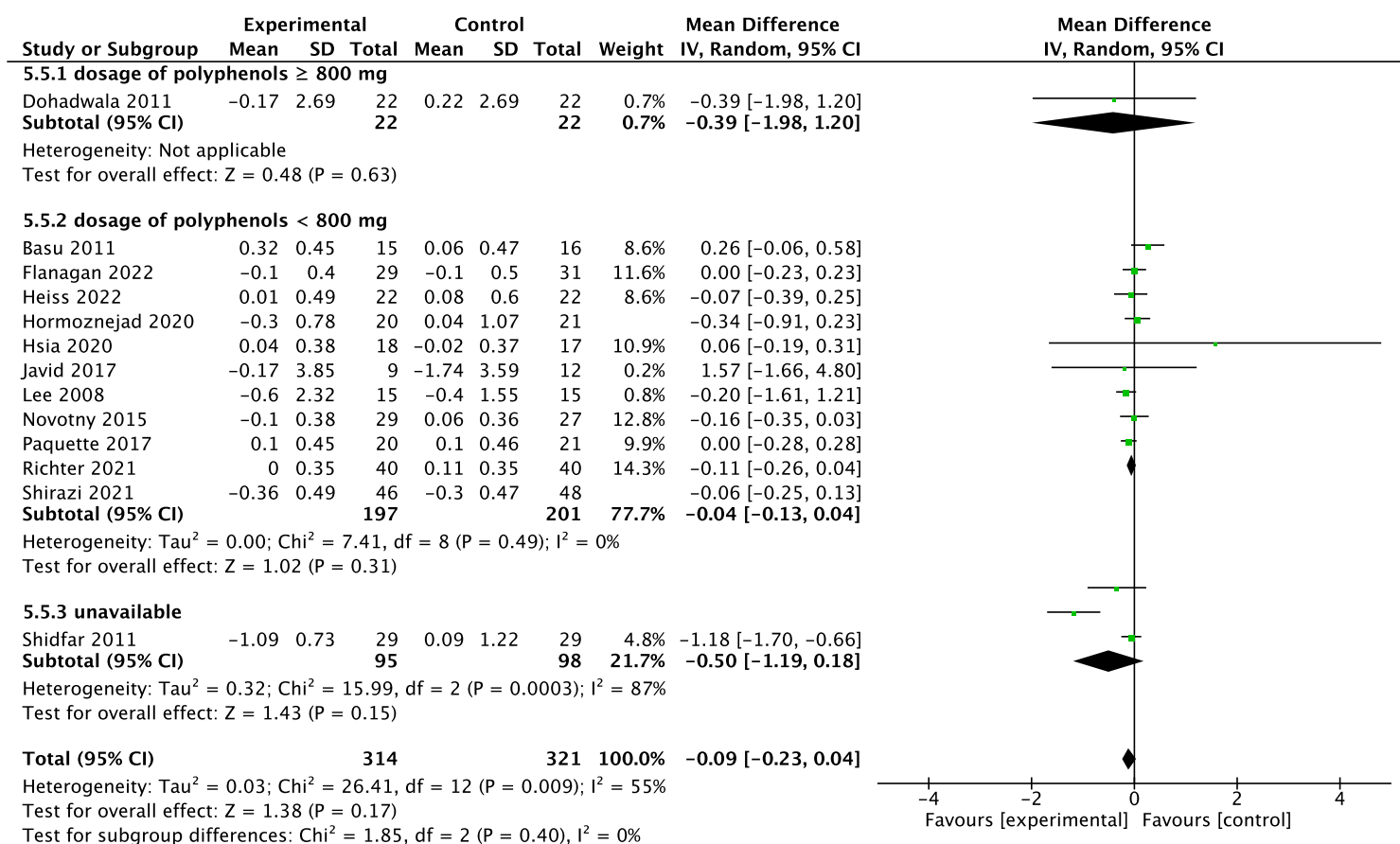

Supplement: Supplementary file 1 [file nutrients-16-00782-s001.zip › Supplementary Figure 28. Subgroup analysis of the effect of the dosage of total polyphenols on FBG.pdf]

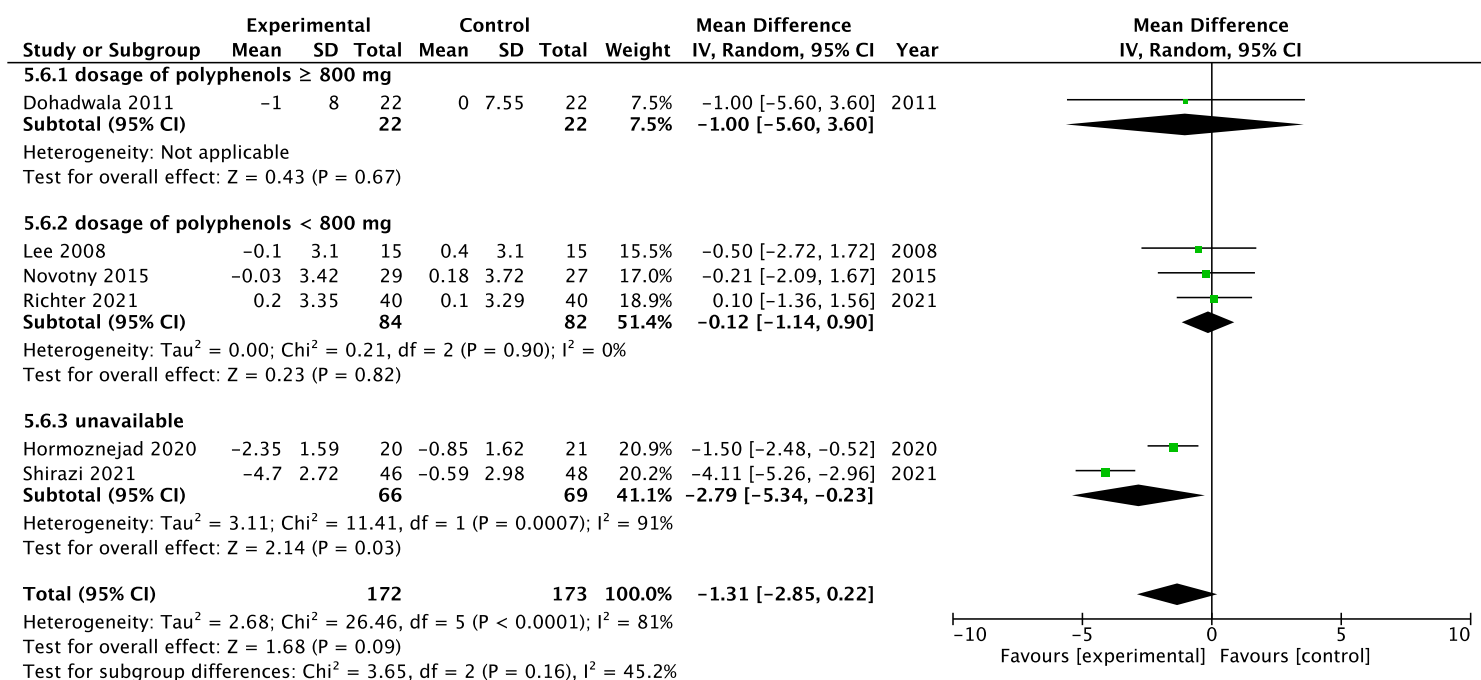

Supplement: Supplementary file 1 [file nutrients-16-00782-s001.zip › Supplementary Figure 29. Subgroup analysis of the effect of the dosage of total polyphenols on fasting insulin.pdf]

Funnel plot with pseudo 95% confidence limits

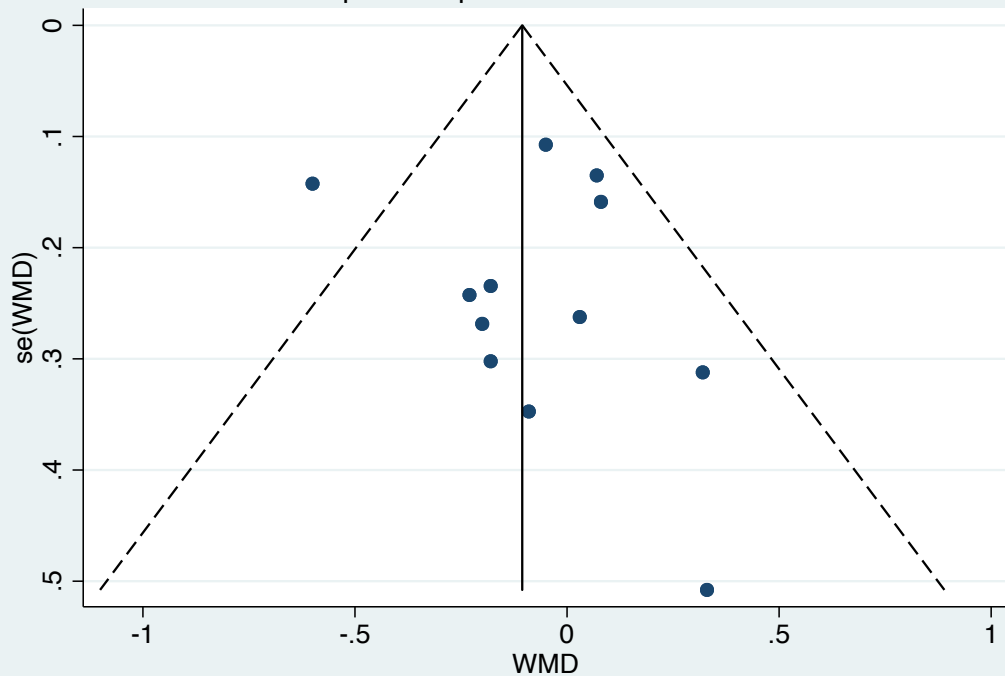

Supplement: Supplementary file 1 [file nutrients-16-00782-s001.zip › Supplementary Figure 3. Funnel plot to evaluate the publication bias for LDL-C.pdf]

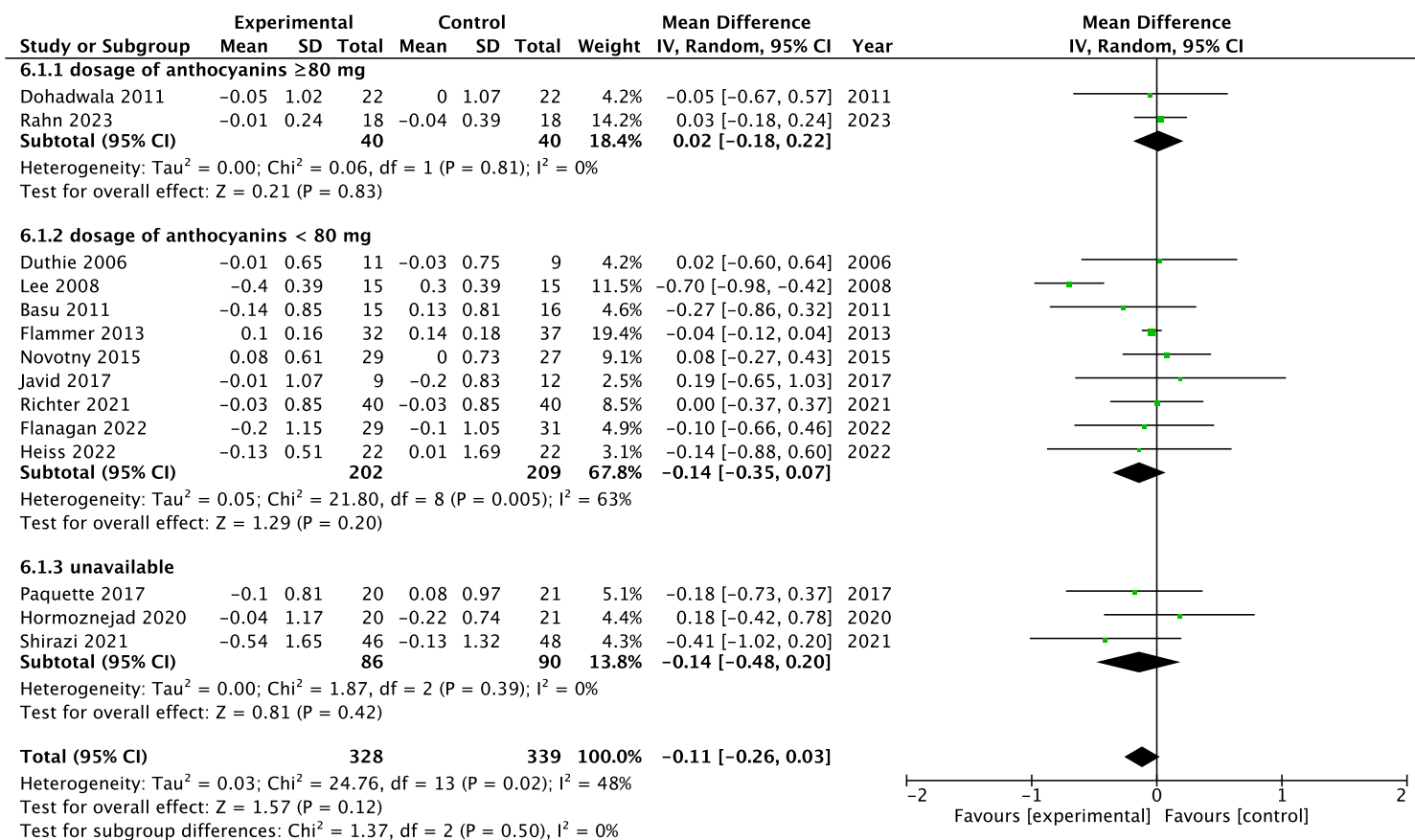

Supplement: Supplementary file 1 [file nutrients-16-00782-s001.zip › Supplementary Figure 30. Subgroup analysis of the effect of the dosage of anthocyanins on TC.pdf]

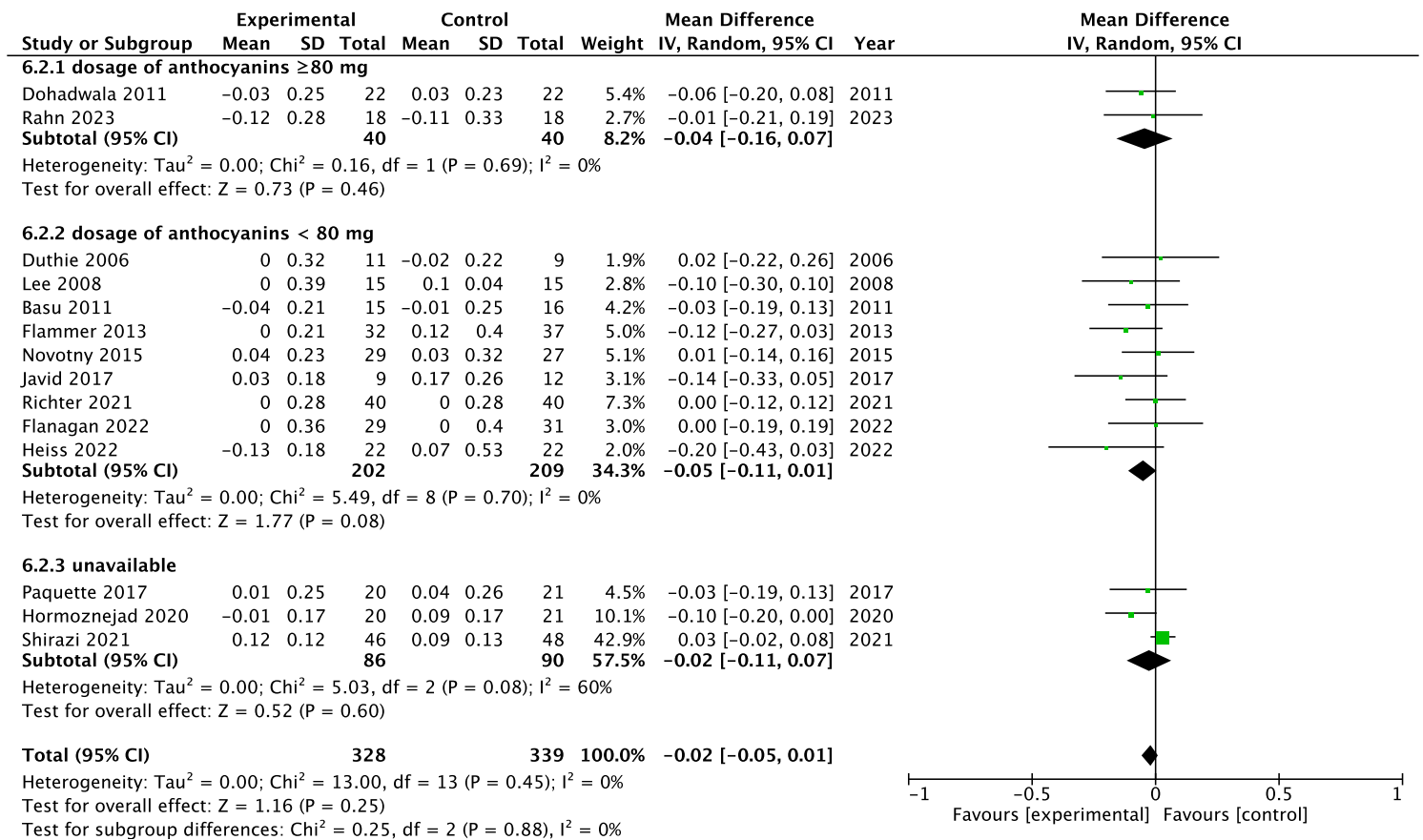

Supplement: Supplementary file 1 [file nutrients-16-00782-s001.zip › Supplementary Figure 31. Subgroup analysis of the effect of the dosage of anthocyanins on HDL-C.pdf]

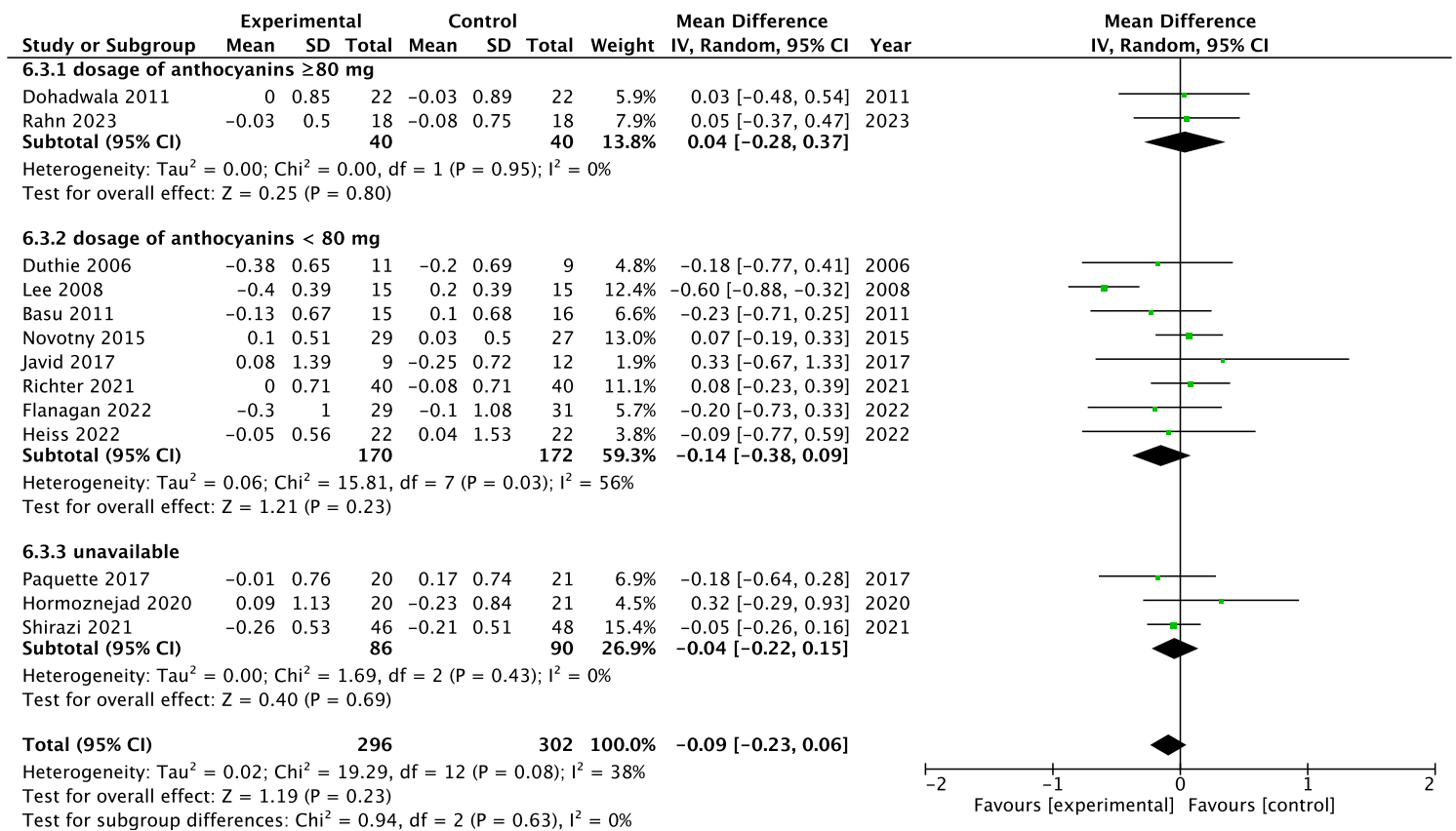

Supplement: Supplementary file 1 [file nutrients-16-00782-s001.zip › Supplementary Figure 32. Subgroup analysis of the effect of the dosage of anthocyanins on LDL-C.pdf]

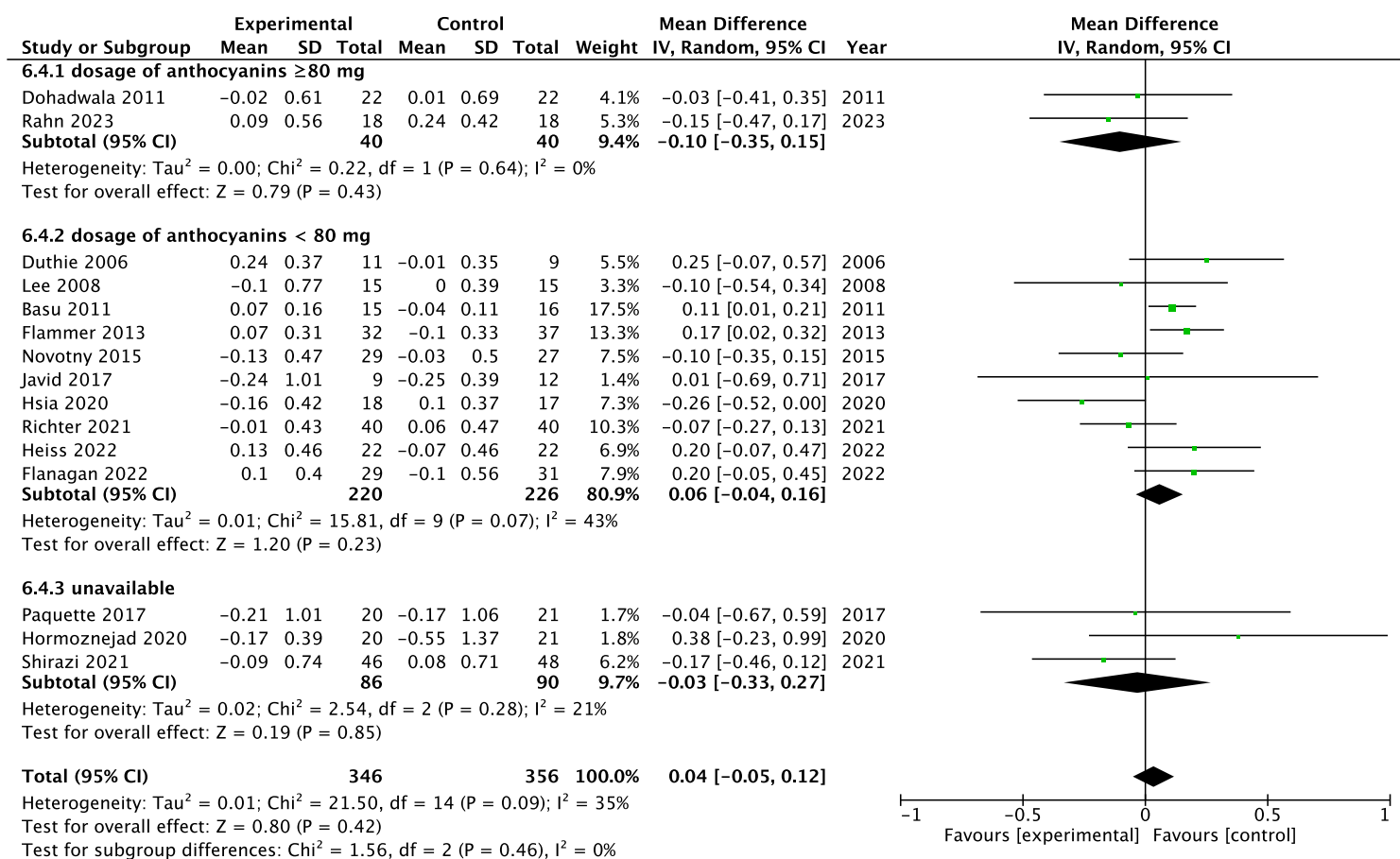

Supplement: Supplementary file 1 [file nutrients-16-00782-s001.zip › Supplementary Figure 33. Subgroup analysis of the effect of the dosage of anthocyanins on TG.pdf]

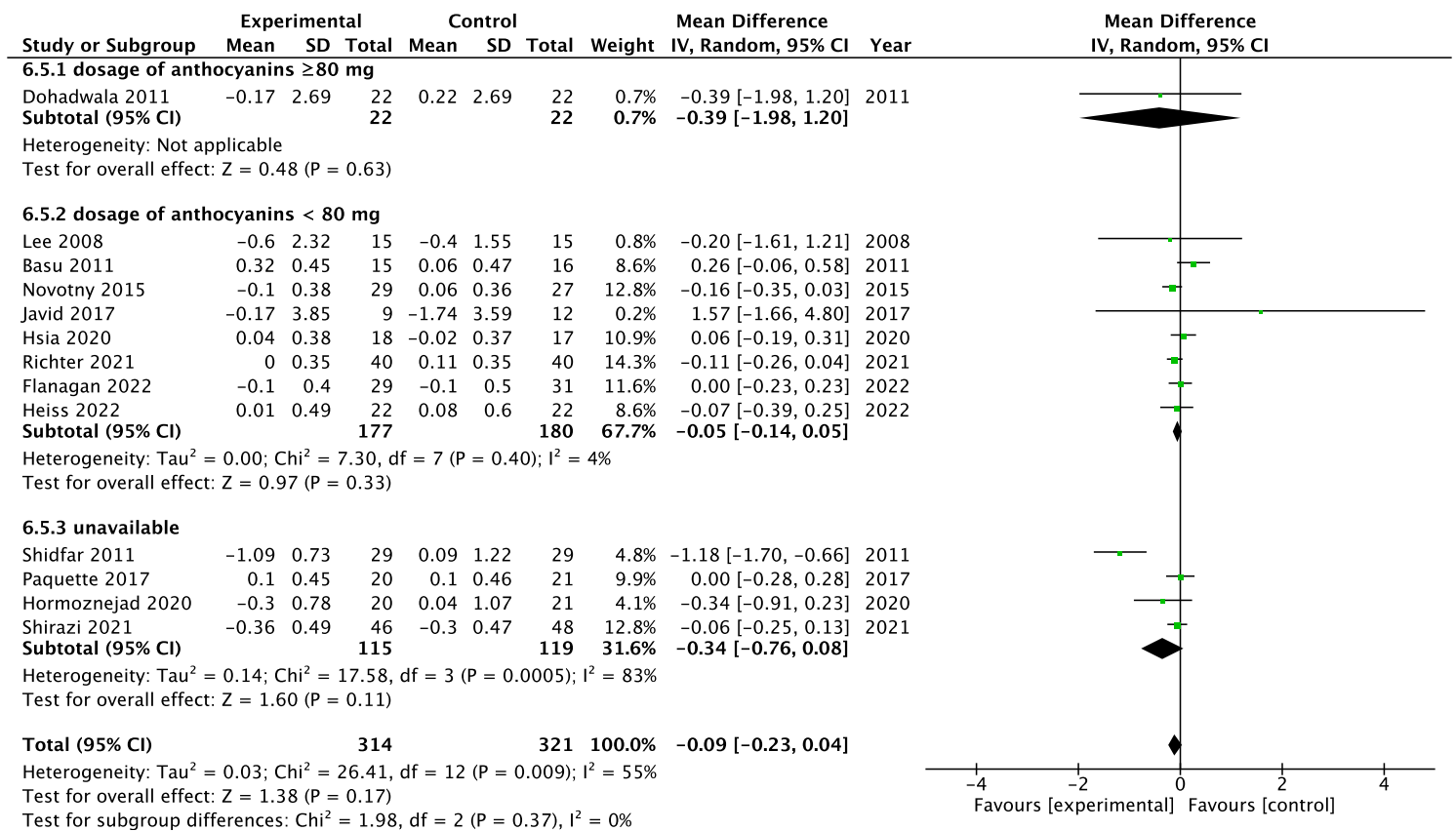

Supplement: Supplementary file 1 [file nutrients-16-00782-s001.zip › Supplementary Figure 34. Subgroup analysis of the effect of the dosage of anthocyanins on FBG.pdf]

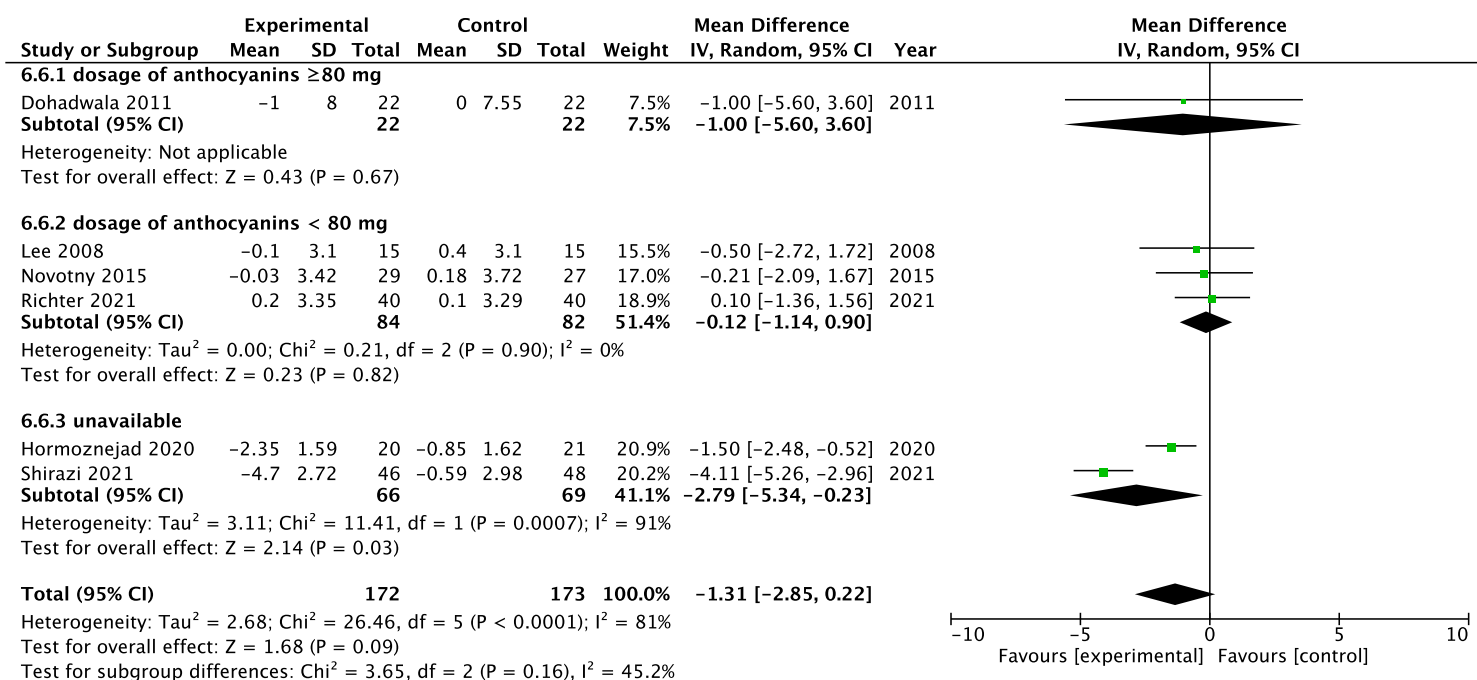

Supplement: Supplementary file 1 [file nutrients-16-00782-s001.zip › Supplementary Figure 35. Subgroup analysis of the effect of the dosage of anthocyanins on fasting insulin.pdf]

Funnel plot with pseudo 95% confidence limits

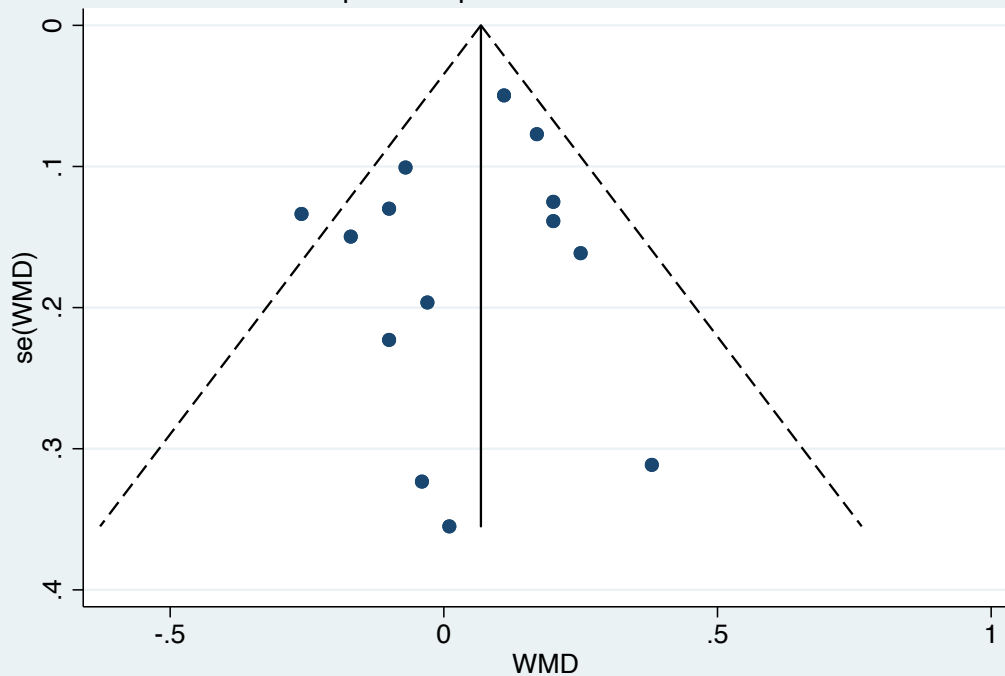

Supplement: Supplementary file 1 [file nutrients-16-00782-s001.zip › Supplementary Figure 4. Funnel plot to evaluate the publication bias for TG.pdf]

Funnel plot with pseudo 95% confidence limits

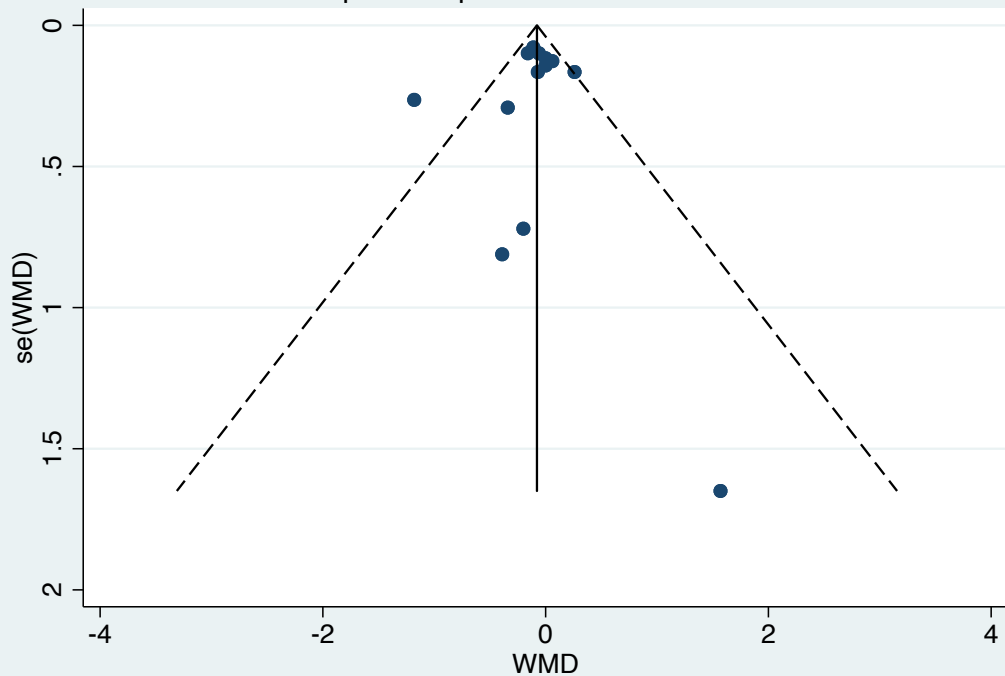

Supplement: Supplementary file 1 [file nutrients-16-00782-s001.zip › Supplementary Figure 5. Funnel plot to evaluate the publication bias for FBG.pdf]

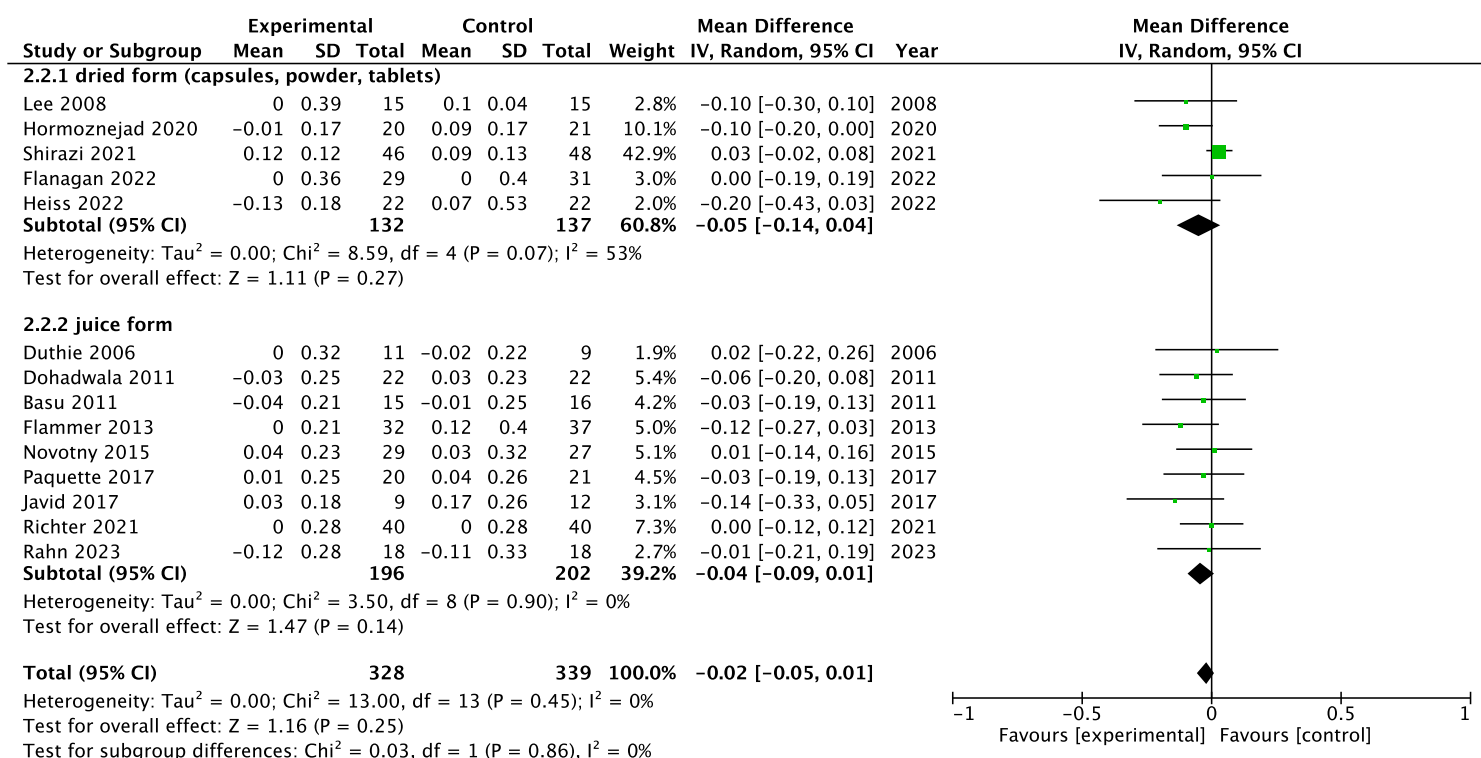

Supplement: Supplementary file 1 [file nutrients-16-00782-s001.zip › Supplementary Figure 7. Subgroup analysis of the effect of dosage form on HDL-C.pdf]

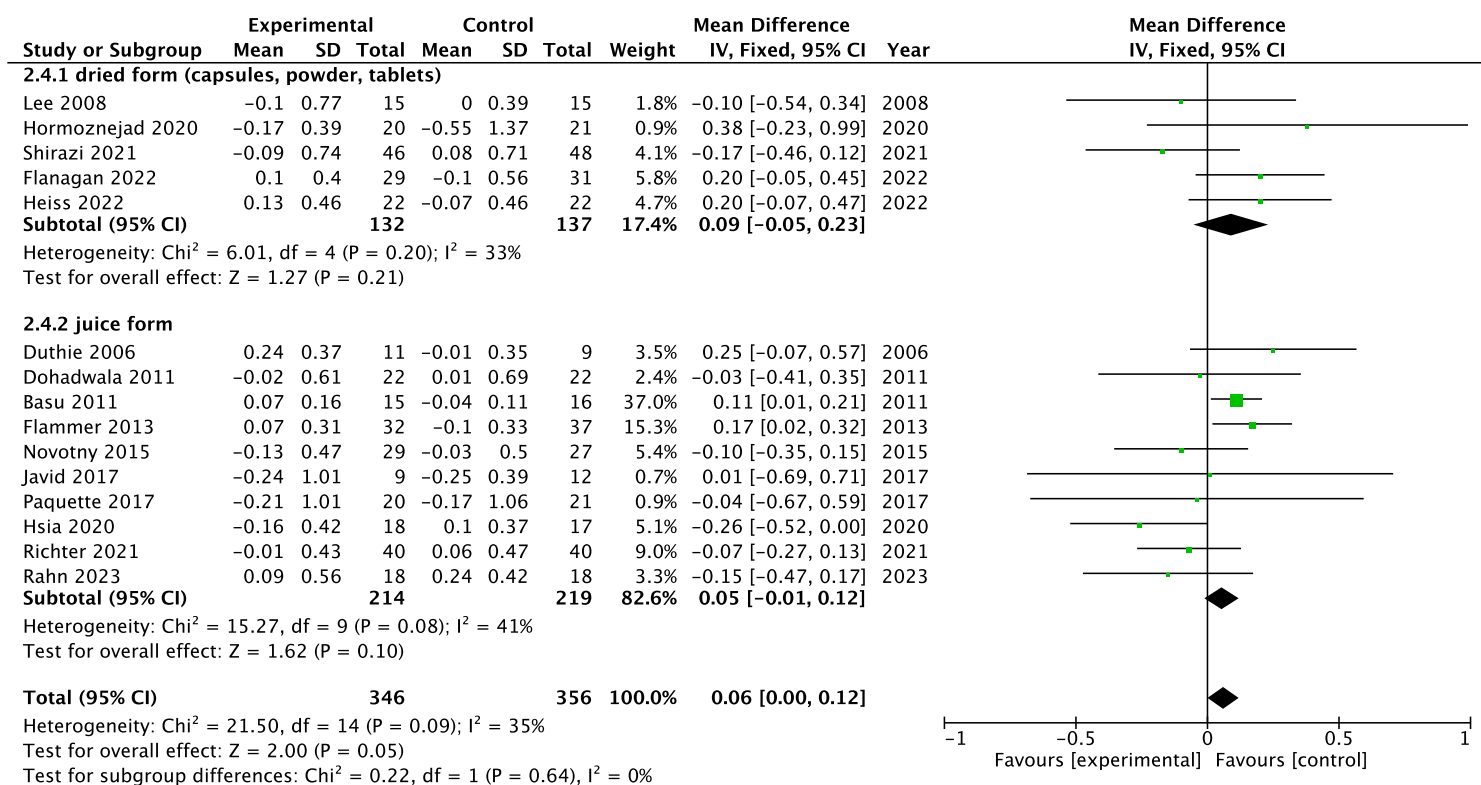

Supplement: Supplementary file 1 [file nutrients-16-00782-s001.zip › Supplementary Figure 9. Subgroup analysis of the effect of dosage form on TG.pdf]
